# Emulsion and Emulgel-Based Ophthalmic Drug Delivery Systems

**DOI:** 10.3390/pharmaceutics17121504

**Published:** 2025-11-21

**Authors:** Debadatta Mohapatra, Eleen Yang, Timothy W. Corson

**Affiliations:** 1 Leslie Dan Faculty of Pharmacy, University of Toronto, Toronto, ON M5S 3M2, Canada; 2 Temerty Faculty of Medicine, University of Toronto, Toronto, ON M5S 3M2, Canada; 3 Department of Ophthalmology and Vision Sciences, University of Toronto, Toronto, ON M5S 3M2, Canada

**Keywords:** ocular delivery, nanoemulsions, microemulsions, self-emulsifying drug delivery system (SEDDS), SNEDDS, SMEDDS, emulgels, in situ gels

## Abstract

Ophthalmic drug delivery encounters unique challenges due to the anatomical and physiological ocular barriers, necessitating the development of novel drug delivery systems (NDDSs). This review focuses on emerging therapeutic platforms, including nanoemulsions (NEs), microemulsions (MEs), self-emulsifying drug delivery systems (SEDDSs) such as self-nano emulsifying drug delivery systems (SNEDDSs) and self-micro emulsifying drug delivery systems (SMEDDSs), emulgels, and in situ-forming emulgels, as novel strategies for enhancing ocular drug delivery. NEs and MEs, due to their small globule size, excellent drug solubility, stability, and bioavailability, offer promising solutions for effective ocular therapy. SEDDSs further enhance the stability and bioavailability of hydrophobic drugs through self-emulsification in aqueous environments. Emulgels, combining the benefits of emulsions and gels, provide sustained and controlled release of therapeutic agents, improving the ocular retention time and therapeutic efficacy. Additionally, in situ-forming emulgels offer the advantage of liquid-to-gel transition upon contact with ocular surfaces, optimizing drug delivery. The review discusses various ocular diseases, challenges for ocular delivery of conventional formulations, updates on emulsion-based novel drug delivery systems for ophthalmic drug delivery, mechanisms of enhanced ocular permeation, formulation strategies, advantages, and challenges, design-of-experiment considerations for optimization, characterizations, and recent advancements in these systems including patents and clinical trials, highlighting their potential for improving the treatment of various ocular diseases. Furthermore, this review explores marketed ophthalmic emulsions and future prospects for integrating these NDDSs into clinical ophthalmology, emphasizing their ability to overcome ocular barriers and enhance therapeutic efficacy.

## 1. Introduction

Topical ophthalmic drug delivery faces numerous challenges, such as poor bioavailability, limited drug penetration across ocular barriers, and the need for frequent administration of therapeutic agents. Traditional ocular formulations, including eye drops and ointments, often fail to offer a controlled or sustained release or targeted drug delivery, leading to suboptimal therapeutic outcomes. The development of novel drug delivery systems (NDDSs) has become increasingly vital in advancing the treatment of ocular diseases. Emerging therapeutic platforms, including microemulsions, nanoemulsions, micelles, nanoparticles, nanosuspensions, liposomes, niosomes, cubosomes, dendrimers, and nanoflowers, have been widely exploited for topical ocular delivery and treatment of ocular diseases [1,2]. Among them, ophthalmic nanoemulsions, microemulsions, self-emulsifying drug delivery systems (SEDDSs), emulgels, and in situ-forming emulgels have achieved significant consideration due to their ability to increase drug solubility, stability, and bioavailability in ocular tissues.

Nanoemulsions (NEs) and microemulsions (MEs) are two distinct emulsions that have shown significant promise for improving the ocular bioavailability of multiple drugs. NEs, typically 20 to 500 nm in size, offer improved drug dissolution and stability over simple drug solutions [3,4,5], while MEs are thermodynamically stable colloidal systems that can increase drug solubility and facilitate enhanced ocular permeation due to their smaller globule size (5–200 nm) and ability to interact with ocular membranes [6].

Self-emulsifying drug delivery systems (SEDDSs) use a self-assembly mechanism that spontaneously forms emulsions upon contact with the aqueous atmosphere of the eye. Under gentle agitation, the SEDDSs produce self-microemulsifying drug delivery systems (SMEDDSs) [7] or self-nanoemulsifying drug delivery systems (SNEDDSs) [8,9]. Due to the high concentration of surfactants (30–60%) in SEDDSs, the required free energy for the formation of emulsions is extremely low [8,10,11]. These systems enhance the solubility of lipophilic drugs and enable controlled, sustained release, decreasing the dosing frequency and increasing patient compliance.

Emulgel formulations, which integrate the advantages of emulsions and gels, are gaining popularity in ocular drug delivery due to prolonged ocular contact, improved drug penetration, and controlled release. The gel matrix provides an advantage in reducing drug loss through drainage, while the emulsion component ensures the efficient delivery of lipophilic drugs [12,13,14].

Moreover, in situ-forming emulgels have emerged as an exciting prospect, providing a unique approach for ophthalmic drug delivery. Upon contact with the ocular surface, these systems form gels in situ, offering enhanced stability, ease of administration, and targeted release of therapeutic agents. In situ gelling systems can offer a significant advantage over traditional formulations by adapting to the physiological conditions of the eye and ensuring controlled drug release over extended periods [6,15].

While previous reviews on NEs [3,4,16,17,18], SNEDDSs [9], MEs [19,20], nanoemulgel [21], and ophthalmic gel [22] have discussed ophthalmic emulsions or emulgels separately, a comprehensive and comparative report covering both liquid systems (NEs, MEs, SNEDDSs/SMEDDSs) and semisolid formulations (emulgels and in situ emulgels) for ophthalmic drug delivery has not been presented to date to our knowledge. This review bridges that gap by integrating recent advances in formulation design, emulsion-based ophthalmic products, and translational aspects, including patents and clinical developments. It outlines briefly various ocular diseases, the challenges of topical ophthalmic delivery, and the limitations of conventional formulations, providing an in-depth update on emulsion-based NDDSs. This includes formulation strategies, mechanisms behind improved ocular bioavailability, advantages and disadvantages, challenges, approaches for excipient selection, design-of-experiment considerations for optimization, detailed characterization techniques, and sterilization methods. Finally, this review highlights the marketing potential and future perspectives on these systems.

Overall, understanding their unique characteristics, formulation challenges, and clinical promise is important for advancing the application of emulsion-based formulations in ophthalmic therapeutics. By critically analyzing ophthalmic emulsions and emulgels, this work highlights the translational potential of emulsion-based NDDSs in overcoming ocular barriers and improving treatment outcomes in ocular diseases.

## 2. Ocular Diseases and Drug Delivery

### 2.1. Ocular Diseases

The ocular diseases are classified into anterior-segment eye diseases and posterior-segment eye diseases. Some major anterior diseases that have been targeted with NEs and MEs include conjunctivitis, pterygium, dry eye disease (DED), keratitis, keratoconus, anterior uveitis, cataracts, and glaucoma (ocular hypertension). Some major posterior diseases include age-related macular degeneration (AMD), diabetic retinopathy (DR), and retinitis pigmentosa.

#### 2.1.1. Conjunctivitis (Pink Eye)

Conjunctivitis is a very common eye disease represented by inflammation of the conjunctiva. It rarely causes lasting vision loss. It is mostly caused by infections, allergens, and hazardous chemicals. Infectious conjunctivitis is mainly caused by adenoviruses and staphylococcal or streptococcal bacteria. Ophthalmia neonatorum is a form of conjunctivitis that occurs at birth due to the transmission from a mother infected with *Chlamydia trachomatis*, which may be severe and cause permanent visual loss if not treated early [1]. Allergic conjunctivitis, the most prevalent form of conjunctivitis, affects 15% to 40% of the population [23]. Treatment of conjunctivitis includes topical antihistamines, steroids, non-steroidal anti-inflammatory drugs, and antibiotics [1,6].

#### 2.1.2. Pterygium

Pterygium is an ophthalmic surface disease represented by a wing-shaped non-cancerous tissue growth of conjunctival and limbal tissue over the adjacent cornea [24]. The cause is not entirely understood, but it is thought to be linked to environmental factors, especially UV radiation, dust, wind, viral agents, inflammatory and immunological factors, and hereditary factors [24]. The worldwide incidence of pterygium is estimated to be around 12% [25]. In the early stages, a pterygium may be asymptomatic or cause only mild irritation, redness, or dryness. As it grows towards the cornea, and it can lead to blurred vision, discomfort, sensation of a foreign body, or eye redness. In severe cases, it may distort the shape of the cornea, leading to astigmatism and further affecting vision. Symptomatic relief is achieved by using artificial tears and lubricating eye drops to alleviate irritation and dryness. Surgical removal is carried out if the pterygium is causing significant discomfort, affecting vision, or growing quickly [24,25,26,27].

#### 2.1.3. Dry Eye Disease (DED) or Keratoconjunctivitis Sicca

Dry eye disease (DED) is a growing multifactorial disorder [28]. Sometimes, it is associated with various systemic autoimmune diseases. DED has a global prevalence of 5–50% due to a lack of sufficient tear fluid or fluid quality for eye lubrication. It is represented by instability of the tear film, inflammation, hyperosmolarity, and damage of ocular surface. DED symptoms include reduced visual acuity, pain, soreness, irritation, ocular heat, and foreign body sensation. The treatment involves the use of drugs increasing tear production (cyclosporine A), anti-inflammatory drugs (dexamethasone, prednisolone), artificial tears with aqueous polymers and small devices (punctal plugs), which slow the drainage of tears [1].

#### 2.1.4. Keratitis

Keratitis refers to corneal inflammation causing corneal opacity, which is the fifth major cause of global blindness [1]. It presents as acute erythema of the eyelids and the conjunctiva, stromal infiltration, corneal ulceration, reduced vision, and pain. Infectious keratitis is caused by acanthamoeba, fungi, viruses, and bacteria, whereas noninfectious keratitis is caused by corneal injury, dry eye, and prolonged use of contact lenses. The incidence of infectious keratitis is reported to range from 2.5 to 799 cases per 100,000 people per year [29]. Treatment strategies include antibiotic therapy and corneal transplants. In addition, certain anti-inflammatory drugs, antibiotics, and immunosuppressants are used to treat complications (inflammation, corneal transplant rejection reaction) after corneal surgery [1,6,30].

#### 2.1.5. Keratoconus

Keratoconus is a bilateral asymmetric ocular disease where there is progressive thinning of the cornea resulting in a cone shape. This abnormal shape can lead to irregular astigmatism, distort vision, and affect the eye’s ability to focus correctly [31,32,33]. The incidence of keratoconus has been reported to range from 1.5 to 25 cases per 100,000 individuals per year [32]. Symptoms, including blurring of vision, distorted vision (glare, photophobia, diplopia, and halos), and monocular polyopia (‘ghost’ images), are mostly observed in keratoconus [32].

The exact cause of keratoconus is not clearly identified; however, eczema, allergy, family history of keratoconus, asthma, eye rubbing, and UV light exposure are common risk factors for the development of keratoconus [31,32]. Mild conditions in early stages are mainly treated with spectacles, and moderate conditions with contact lenses, while severe keratoconus requires corneal surgery. Mild to moderate cases of keratoconus can also be treated surgically, mainly by corneal cross-linking [31,32,33]. Various laser techniques are used to regularize the cornea and reduce more subtle and complex refractive errors [32,33].

#### 2.1.6. Uveitis

Uveitis is an acute inflammation mainly affecting the uveal tract (iris, ciliary body, and choroid) [34,35,36]. It also extends to the sclera, vitreous region, and back of the eye, including the retina and optic nerve [34]. Based on the inflammation site, uveitis is classified into anterior (anterior chamber), intermediate (vitreous body), posterior (retina and choroid), and panuveitis (all layers of the uvea) [6,36]. Uveitis contributes to approximately 10% of blindness cases worldwide [34]. Patients with uveitis present with conjunctival redness, eye pain, and photophobia [34]. The acute and chronic inflammation may lead to vision loss via the development of cataracts, damage to the optic nerve, inflammation of the vitreous, development of synechiae, permanent deterioration of the blood–aqueous barrier, and cystoid macular swelling if it remains untreated [34]. The treatment includes corticosteroids and anti-inflammatory agents (e.g., dexamethasone sodium phosphate), either as monotherapy or in combination with other immunosuppressants [6,34,35,36].

#### 2.1.7. Cataract

Although cataract-related blindness is fully reversible, it accounts for over 50% of blindness cases worldwide [37]. It is represented by clouding of the eye lens, which develops with age, leading to vision impairment. With age, the proteins of the lens tend to aggregate, which leads to clouding and decreases the quantity of transmitted light that reaches the retina. Lens replacement surgery is used for the treatment of cataracts. Post-operative adverse effects, such as suprachoroidal hemorrhage and endophthalmitis, are rare but require drug treatment [1,37]. Antibiotics (moxifloxacin, vancomycin, ceftazidime) and corticosteroid therapy (dexamethasone) are used for the management of endophthalmitis [38], whereas intraocular pressure (IOP)-reducing drugs (timolol, acetazolamide) and steroids (prednisolone acetate) are used for the management of suprachoroidal hemorrhage [39].

#### 2.1.8. Glaucoma (Ocular Hypertension)

Glaucoma is represented by death of retinal ganglion cells (RGCs), which is mostly associated with high IOP. It is another major driver for vision loss globally [40,41]; estimates indicate that by 2040, about 111.8 million people globally will be affected [40]. It is categorized by open-angle, closed-angle, and congenital glaucoma [41]. In open-angle glaucoma, the drainage angle is open; however, the eye’s drainage system becomes less efficient due to increased production or decreased outflow of aqueous fluid. In contrast, closed-angle glaucoma is acute and occurs due to the narrowing or blocking of the angle between the cornea and iris [40]. The ocular fluid cannot drain properly in both cases, leading to increased ocular pressure [1,6]. Closed-angle glaucoma requires quick treatment by surgical methods or laser to avoid optic nerve injury. Open-angle glaucoma can be treated by reducing IOP by decreasing the secretion of aqueous humor and/or enhancing its outflow [41]. Prostaglandin analogs, α-agonists, β-blockers, and carbonic anhydrase inhibitors are the most common agents used for glaucoma treatment [41,42,43]. Surgical procedures for glaucoma include trabeculectomy, minimally or microinvasive glaucoma surgery, glaucoma drainage devices, and cyclodestructive procedures. These target existing trabecular outflow, improve suprachoroidal outflow, reduce aqueous production, or create subconjunctival blebs [44].

#### 2.1.9. Age-Related Macular Degeneration (AMD)

Nearly 200 million individuals globally are affected by some form of AMD [45]. It affects the macula, leading to progressive loss of visual functions [45,46,47,48]. Genetic factors, older age, hyperlipidemia, family history, ethnicity, and environmental factors, like smoking cigarettes and lower physical activity, are associated with AMD development [47]. There are two major types of AMD, dry AMD (degenerative) and wet (neovascular AMD/nAMD or exudative AMD) [45,48]. Dry AMD is represented by deposition of drusen (lipid and protein) under the retinal pigment epithelium (RPE), thickening of Bruch’s membrane, and progressive death of photoreceptors, RPE, and choriocapillaris in the macula [45,48]. Wet AMD involves macular neovascularization (MNV) with new vessels developing from the choroid or deep retinal capillary bed to the RPE or through the RPE to the subretinal region. The development of abnormal blood vessels from the choroid penetrates through Bruch’s membrane, resulting in scarring, RPE detachment, hemorrhage, and exudations [48]. Photodynamic therapy (PDT), laser therapy, and intravitreal injection of anti-vascular endothelial growth factor (VEGF) drugs (bevacizumab, ranibizumab, faricimab, brolucizumab, aflibercept, and conbercept) are mostly preferred for the treatment of nAMD [6,45,47,48].

#### 2.1.10. Diabetic Retinopathy (DR)

Diabetic retinopathy is one of the major causes of vision loss across the globe, caused by the high blood sugar level due to diabetes mellitus [49]. It occurs in about 30 to 40% of diabetic patients. The worldwide prevalence and impact of DR are projected to rise significantly in the coming decades, increasing from ~103 million people in 2020 to 130 million by 2030, and reaching 161 million by 2045 [50]. Patients observe black, dark areas, and floaters in the vision field [46]. DR is represented by multiple pathological events, mainly inflammatory reactions and oxidative stress due to hyperglycemia [51]. DR is categorized into early-stage, non-proliferative diabetic retinopathy (NPDR) and late-stage, proliferative diabetic retinopathy (PDR) [52]. NPDR involves damage to retinal capillaries and microaneurysms, leading to leakage of blood and fluid without neovascularization, whereas PDR involves retinal ischemia, neovascularization, leakage from retinal neovessels, retinal detachment, and loss of vision [52]. Diabetic macular edema involves the leakage of fluid from damaged capillaries, leading to thickening of the macular retina and blurred or distorted central vision. Therapies for DR include vitreoretinal surgery, anti-VEGF drugs/biologics as above, small-molecule glucocorticoid receptor agonists (conbercept, dexamethasone), and lipoprotein lipase stimulator (fenofibrate) [51]. In addition to the above, laser therapy, especially panretinal photocoagulation (PRP), is also used in the management of DR [51].

#### 2.1.11. Retinitis Pigmentosa (RP)

Retinitis pigmentosa is an inherited ocular disease that leads to progressive retinal degeneration. It is represented by highly impaired rod function and cone function [53,54]. Among Western populations, RP is estimated to occur in roughly 1 out of every 3000 to 5000 people [53]. The degeneration of rod photoreceptors (which offer achromatic night vision) and cone photoreceptors (which offer high-acuity central color vision) leads to RP [54]. The degeneration of rod cells leads to the death of cone cells [54]. The loss of vision worsens over time, first affecting night vision and peripheral vision, followed by central vision loss in advanced stages [53]. The experimental treatment strategies include gene therapy (gene augmentation therapy, CRISPR/CAS9-based therapy, antisense oligonucleotides), gene-independent strategies (optogenetics, stem cell therapy, retinal prostheses, neurotrophic factors, neuroprotective agents), nutritional therapies (vitamin A, lutein and docosahexaenoic acid (DHA) supplementation), and retinal implants [53,54,55].

### 2.2. Challenges for Topical Ocular Delivery

**Figure 1:**
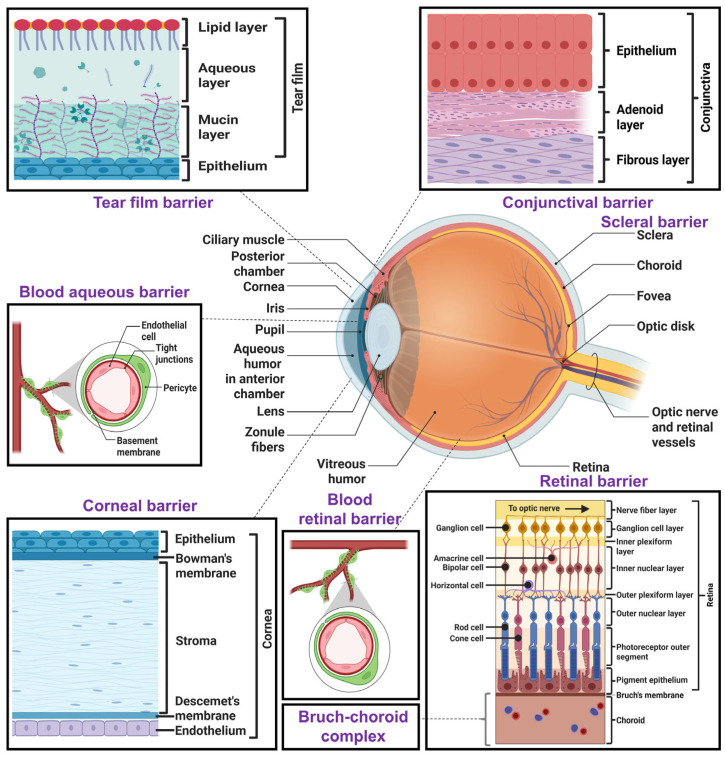
Graphical illustration of various structural ocular barriers in human eye. This figure illustrates the key anatomical barriers that influence topical ocular drug delivery, including the pre-corneal tear film, corneal epithelium, conjunctiva, sclera, Bruch’s–choroid complex, and retinal barrier. In addition to these, it also represents some systemic ocular barriers, such as the blood–aqueous barrier and blood–retinal barrier. Created with biorender.com.

Topical ocular drug delivery offers good patient compliance, low cost, and site-specific drug delivery, minimizing systemic side effects. But ocular barriers make topical success challenging. These ocular barriers are classified into pre-corneal, corneal, and blood–ocular barriers. The ocular anatomy with various ocular barriers is shown in Figure 1. The topically administered drugs suffer from poor absorption and ocular bioavailability due to the presence of pre-corneal, corneal, and conjunctival barriers, efflux pumps, and melanin binding. Other barriers, such as the blood–aqueous barrier (BAB), blood–vitreous barrier (BVB), and blood–retinal barrier (BRB), act as potential ocular barriers for systemically administered drugs.

#### 2.2.1. Pre-Corneal Barriers

The pre-corneal barriers restrict the penetration of topically administered ocular treatments before they reach the cornea [1]. These include the tear film barrier, protein binding and metabolism, reflex blinking, limited capacity of the cul-de-sac (conjunctival fornix), nasolacrimal drainage, and tear turnover.

##### Tear Film Barrier

The tear film comprises an oily outer layer, aqueous middle layer, and mucin-based inner layer that act as the first barrier to topical ophthalmic formulations. The outermost lipid layer acts as a barrier for hydrophilic drugs, whereas the middle aqueous layer acts as a barrier for hydrophobic drugs. The innermost mucin layer is anionic in nature and interacts with cationic drugs electrostatically, but it repels anionic drugs and formulations [1,6]. The rate of tear flow is 1–3 µL/min under normal physiological conditions, renewing the tear film every 5 min. After topical ocular application, the volume increases, leading to stimulation of the reflex and increased secretion of tears. This leads to dilution of the dose, washing out by the nasolacrimal duct, and poor ocular bioavailability [6].

##### Protein Binding and Metabolism by Enzymes

The aqueous portion of the tear film contains metabolizing enzymes (esterase, cytochrome P-450, and peptidases) and proteins (lysozyme, albumin) that can metabolize or cause protein–drug binding of the topically administered drugs or peptides, ultimately decreasing the fraction of free drug/peptides and their therapeutic activity. Under normal conditions, the protein and metabolizing enzyme levels are low; however, their level significantly increases during inflammation or diseased conditions [1,56]. Various enzymes, such as oxidoreductases (aldehyde oxidase, cytochrome P450, cyclooxygenase, monoamine oxidase, glutathione peroxidase), hydrolases (carboxylesterase 1 and 2, fatty-acid amide hydrolase, arylacetamide deacetylase, paraoxonase), reductases (aldo/ketone), transferases (glutathione S-transferases, N-acetyl transferase), and peptidase (cystine aminopeptidase) expressed in the cornea, ciliary body, iris, lens, and retina can metabolize active drugs and peptides, thereby decreasing ocular bioavailability [57,58,59].

##### Reflex Blinking

During topical ophthalmic administration of formulations, the blinking of the eye helps to spread the formulation across the cornea and conjunctiva. However, the reflex blinking also quickly washes out the administered drug from the eye, reducing the ocular contact time with the epithelium and drug absorption. Blinking also stimulates tear production and drainage. Tears further flush out the drug and contain enzymes and other substances that may degrade the drug, further reducing the residence time.

##### Limited Capacity of the Cul-De-Sac

Most of the topical ophthalmic drops are applied to the cul-de-sac, with very limited capacity (nearly 30 µL in the case of humans), and are further decreased to 70–80% due to the eyelid movement. That is further reduced in pathological events, such as allergy and inflammation [1,3]. The low volume of the cul-de-sac offers low retention of ocular formulations.

##### Nasolacrimal Drainage and Tear Turnover

After ocular drug administration, the drug is mainly eliminated by lacrimation and drainage, which maintains a regular tear volume (7–9 μL) [3]. The nasolacrimal duct drains the topically applied drugs into the nasal cavity, where they are absorbed into the bloodstream [1]. The tear turnover rate (14.9%/minute) reduces the ocular contact time, washes out formulations, and ultimately reduces their therapeutic effect [1]. Ophthalmic formulations, including pH-triggered or electrolyte-sensing in situ gels, improve the ocular retention time by avoiding solution lacrimation and drainage [3].

#### 2.2.2. Corneal Barriers

The cornea acts as an impermeable second barrier to a variety of chemicals and drug molecules. Hence, transcorneal diffusion is considered an important step for the permeation of drugs into the aqueous humor and their distribution to other ocular tissues. Despite having a surface area of less than 6% of the total ocular surface, the cornea is primarily impermeable because of its several hydrophilic and lipophilic layers. The cornea comprises three major layers: (i) epithelium, (ii) stroma, and (iii) endothelium, obstructing drug absorption into the eye. Physicochemical characteristics of drugs, such as molecular weight, hydrophobicity, and degree of ionization, influence the corneal permeability [1]. Lipophilic corneal layers prevent the entry of hydrophilic drugs, and the hydrophilic region restricts the permeation of lipophilic drugs [1]. Only small drug molecules with optimal lipophilicity/hydrophilicity can effectively permeate across corneal layers. The epithelial cells of the cornea possess paracellular tight junctions with pore sizes of 2.0 nm, which act as potential permeation barriers for hydrophilic drugs [1]. In general, drugs with a molecular weight of more than 500 Da or a molecular size of greater than 5.5 Å cannot permeate the epithelium of the cornea via paracellular transport [1]. The stroma comprises 90% of the cornea, which is hydrophilic in nature due to its high water content. This layer allows the permeation of drugs up to 500 Da; however, it restricts the permeation of lipophilic drugs [1]. The endothelium comprises a monolayer of cells with intercellular tight junctions that obstruct the permeation of hydrophilic drugs. However, due to the lower thickness, the endothelium barrier is weaker. The endothelial porous cell network allows the permeation of macromolecules up to 70 kDa [1]. The presence of sialic acid residues on the apical part of the epithelium results in a negatively charged corneal surface. Therefore, negatively charged molecules and nanoformulations may penetrate relatively more slowly than positively charged drugs/formulations [1]. Cationic NEs/MEs offer improved transcorneal permeation via interaction with the anionic corneal membrane [3]. The pre-corneal and corneal barriers reduce the ocular bioavailability (<5%) of topically applied drugs [1,60].

#### 2.2.3. Conjunctival Barrier

The conjunctiva also acts as a significant barrier to the permeation of topically administered drugs. However, the conjunctival surface area is 17–20 times more than that of the cornea and possesses a broader intercellular space with relatively higher drug permeability. The existence of lymphatic and blood vessels causes the outflow of drugs that decrease the ocular bioavailability [1,17]. The conjunctival path allows the absorption of large hydrophilic molecules (<20 kDa), such as peptides and proteins, whereas the corneal pathway mainly allows small lipophilic drugs [17].

#### 2.2.4. Scleral Barrier

The sclera is comprised of glycoproteins, proteoglycans, and collagen fibers, which maintain the eyeball’s shape and prevent the entry of foreign particles into the posterior segment of the eye [6]. Thus, the sclera also acts as an effective barrier to the ocular permeation of drugs. The permeation of drugs across the sclera increases with a decrease in lipophilicity and vice versa. Larger-molecular-weight drugs with high lipophilicity suffer poor permeation through the aqueous pores of the sclera [6]. The permeation of drugs is also affected by the intraocular pressure. A normal intraocular pressure (15–20 mmHg) has a negligible effect on the permeation, whereas increased intraocular pressure (>20–60 mmHg) decreases the permeability significantly due to the compression of ocular tissue and alteration of tissue microanatomy [17,61]. The charge of drug molecules also affects their permeation across the sclera. Cationic drugs are comparatively less permeable than anionic drugs since the proteoglycan layer of the sclera is negatively charged. The positively charged molecules interact with the negatively charged proteoglycan matrix, obstructing their permeation across the sclera [6].

#### 2.2.5. Vitreous Humor

The vitreous fluid is a highly hydrated semisolid fluid comprising 99% water and non-collagenous proteins; proteoglycans of chondroitin sulfate; types I, V, IX, and XI collagens; hyaluronic acid; and heparan sulfate. The viscous vitreous gel restricts the diffusion of drugs from the vitreous fluid to the retina [6]. Large, lipophilic/hydrophilic drugs are retained more in the vitreous humor [17]. Charged and larger drug molecules may interact with anionic collagen or hyaluronic acid, leading to aggregation and precipitation in the vitreous fluid, making the drug difficult to absorb [6].

#### 2.2.6. Retina

The retina itself restricts the permeation of drugs from the vitreous fluid into the retina. The retina has a 15–20 nm intracellular space without any tight junctions, which allows the permeation of small lipophilic and hydrophilic drugs. Cationic larger molecules suffer poor penetration into the retina. The outer and inner plexiform layers are the major barriers to the permeation of large-molecular-weight drugs into the retina [17]. The RPE forms the outer BRB through tight junctions, which maintains retinal homeostasis and regulates the transport of drugs from choroid to retina by restricting passive drug diffusion [1].

#### 2.2.7. Choroid and Bruch’s Membrane Barrier

The choroid is a pigmented layer between the retina and sclera, covering the posterior portion of the eye. It comprises fenestrated capillaries that supply blood to the outer retina and the RPE, which is supported by an elastic Bruch’s membrane [6].

The choroidal thickness decreases with age, whereas the thickness of Bruch’s membrane increases with age. Thus, the barrier function is altered with age [17]. The Bruch’s–choroid (BC) complex acts as a significant barrier to transscleral drug transport, especially for cationic hydrophobic drugs [6].

#### 2.2.8. Efflux Pumps

In addition to barriers of ocular layers, the efflux pumps in ocular cells cause the efflux of drugs. Mainly, they are known as permeability glycoprotein (P-gp) and multidrug resistance protein (MRP), which are expressed in the cornea, conjunctiva, ciliary body, iris, and RPE. P-gp and MRP act as potential efflux pumps, causing the efflux of the administered drug and reducing the intracellular drug absorption and concentration. Human corneal efflux transporters, such as MRP1-4 and MRP6, are mainly localized in the basal layer of the corneal epithelium, whereas MDR1 and MRP7 are expressed in the entire corneal epithelium. MRP6, MDR1, MRP2-4, and BCRP are expressed in the basal cell layer of human conjunctiva, whereas MRP1 and MRP7 are found in the entire conjunctival epithelium. MDR1 and MRP1-2 are mostly found in stromal cells of the human iris ciliary body [62]. These efflux pumps reduce the ocular bioavailability of topically administered drugs.

#### 2.2.9. Melanin Binding

Melanin pigment is found in the ciliary body, iris, RPE, and choroid. It binds reversibly to lipophilic and basic drugs, decreasing the amount of free drugs. Similar to protein–drug binding, melanin–drug binding also affects the pharmacokinetics of ophthalmic drugs [1]. As only free drugs participate in drug distribution, melanin binding of drugs sequesters a significant fraction of topically administered drugs, reducing the free active drug at target sites and thereby lowering ocular bioavailability. However, the melanin-bound drugs act as reservoirs, and a small fraction of bound drug can release at a slow rate over a longer duration [57,63].

#### 2.2.10. Blood–Ocular Barriers

These barriers are not relevant to topical ocular drug delivery but also pose significant challenges for delivering drugs from the systemic circulation. Certain specialized ocular barriers, the blood–aqueous barriers, blood–vitreous barriers, and blood–retinal barriers, obstruct the permeation of drugs from blood to the eye [1]. These ocular barriers shield the eye from the entry of toxic materials and systemically administered drugs. The BAB exists at the anterior part of the eye, which restricts the nonspecific entry of solutes into the aqueous humor. It allows the entry of lipophilic and low-molecular-weight drugs [3]. It consists of the iris endothelium, ciliary muscle, posterior iris, and non-pigmented ciliary epithelium [1]. The BVB prevents the entry of drugs from blood to the vitreous fluid. The BRB is a posterior barrier composed of RPE cells and retinal endothelium. The RPE and endothelial tight junctions prevent the paracellular transport of hydrophilic drugs and high-molecular-weight drugs from the systemic circulation into the retina [1]. NEs or MEs are reported to cross these barriers effectively [3].

### 2.3. Conventional Drug Delivery Systems, Their Limitations, and the Importance of NDDSs, Especially Emulsions

Conventional formulations for topical ocular drug delivery include solutions, gels, ointments, emulsions, and suspensions [40]. Topically administered ophthalmic drugs through conventional formulations suffer from poor ocular bioavailability due to the small corneal surface area, complex anatomical barriers, and physiological processes such as conjunctival absorption, tear turnover, induced lacrimation, drug metabolism, drainage, and drug retention in the aqueous and vitreous fluids. Consequently, less than 5% of the topically administered dose reaches the aqueous humor, necessitating high drug concentrations. These formulations provide pulse delivery with an initial high concentration followed by rapid clearance, requiring frequent dosing and resulting in poor patient compliance. In addition, rapid drug drainage from tear production and reflex blinking, poor corneal permeation, and lack of bioadhesion reduce the ocular residence time and contact with the target tissues. Systemic absorption through the nasolacrimal duct may also cause adverse reactions, such as hypertension, hypotension, cardiac arrhythmias, myocardial infarction, drowsiness, rashes, facial flushes, and acute asthma attack [64]. Due to the aforementioned limitations of conventional topical ophthalmic formulations, they lead to limited pharmacotherapeutic efficacy [3,15,16,40,60].

## 3. Emulsion-Based Ophthalmic Drug Delivery Systems

Multiple novel drug delivery systems, such as nanoemulsions, microemulsions, self-emulsifying systems, liposomes, nanoparticles, nanosuspensions, niosomes, micelles, nanofibers, dendrimers, solid lipid nanoparticles, spanlastics, and hydrogels, have been reported for ocular drug delivery with promising therapeutic activity [65,66]. Emulsions are liquid dosage forms comprising two immiscible liquids in which one phase is dispersed in another liquid phase and stabilized by an emulsifying agent. The external phase is referred to as the dispersion medium or continuous phase, whereas the internal phase is called the dispersed phase. Surfactants are mostly used as emulsifying agents [4]. Emulsions are broadly classified into single and multiple-emulsion systems. The single emulsion is prepared using the single-step method, whereas multiple steps are used to prepare the multiple emulsions. A single emulsion can be an oil-in-water (o/w) or water-in-oil (w/o) type. Multiple emulsion includes the oil/water/oil (o/w/o) or water/oil/water (w/o/w) type [4]. The multiple emulsions are multi-layered and possess combined advantages of both types (w/o and o/w) of microemulsions while avoiding their individual drawbacks [67]. Such liquid dosage forms are widely exploited for various routes of drug administration, including oral, topical, intravenous, intranasal, and topical ocular drug delivery.

Advantages of ophthalmic emulsions

The ophthalmic emulsions offer the following [1,3,40,60]:

- Optical transparency, causing no blurred vision.
- Improved aqueous solubility of hydrophobic drugs.
- Delivery of both lipophilic and hydrophilic drugs.
- Enhanced wettability by reducing the contact angle due to surfactants.
- Increased permeability across ocular barriers.
- Prolonged ocular contact time and improved bioavailability.
- A non-invasive route of ocular drug administration.
- Reduced dosing frequency, leading to increased patient compliance.
- Improved physical and chemical stability of the formulation.
- Prolonged shelf life of the loaded drugs.
- Sustained or controlled drug release.
- Avoidance of frequent administration at high concentrations, minimizing potential toxicity.
- Ease of sterilization of the formulation.
- Inhibition of P-gp efflux activity on corneal and retinal epithelial cells when suitable surfactants are used.
- Opening of tight junctions, thereby improving drug penetration.
- Negligible irritation when non-ionic surfactants are incorporated.

Disadvantages

In addition to the advantages of emulsions for ocular drug delivery, they have several shortcomings [3], such as the following:

- Low viscosity and low ocular retention; hence, gelling agents are introduced to increase the viscosity.
- Potential for ocular cytotoxicity due to the large quantity of surfactants in NEs and MEs.

### 3.1. Nanoemulsions

Nanoemulsions (NEs) are kinetically stable liquid dosage forms with two immiscible liquids (oil and water) stabilized by surfactants/cosurfactants with a globule size of 20–500 nm [3,4,5]. They are also called “miniemulsions” [4,16]. The NEs comprise oil, water, and surfactants/cosurfactants. Sometimes, cosolvents are added to the NEs to enhance their colloidal properties and stability. Based on the dispersion phase and continuous phases/dispersion medium, they are classified into o/w, w/o, and a bi-continuous type, in which oil and the aqueous phase are interdispersed within the NEs [3,16]. Among the NEs, the o/w type is widely used for ophthalmic drug delivery as the water is in the external phase, and oil is in the internal phase, which can solubilize and load lipophilic drugs and allow maximum retention and improved ocular permeation [3,4]. It is one of the frequently used non-invasive ophthalmic formulations due to its ability to (i) provide high aqueous solubility, (ii) improve the solubility of poorly soluble drugs, (iii) ensure excellent permeability across ocular barriers, (iv) increase contact time and pre-corneal retention in the eye, (v) decrease the contact angle and prolong the drug dwelling time through electrostatic interactions with the anionic mucin layer of the cornea, (vi) reduce the dosing frequency, (vii) improve stability, (viii) offer sustained or controlled drug release, (ix) maintain optical transparency, (x) enhance patient compliance, and (xi) exhibit high scale-up potential [3,4,16,40]. The lipid material of NEs interacts with the lipid layer of the tear film, which allows prolonged retention of the ocular formulation and acts as a drug depot [3]. The cationic NEs decrease the contact angle and interact with the anionic mucin layer of the cornea, further prolonging ocular retention [3]. However, they possess certain disadvantages, such as (i) ocular irritation due to a high surfactant concentration, (ii) a low residence time due to low viscosity, and (iii) occasional instability: precipitation, phase separation, flocculation, and coalescence [3].

### 3.2. Microemulsions

There is a lot of similarity between NEs and MEs. MEs are thermodynamically stable, isotropic liquid dosage forms composed of oil, surfactant, cosurfactants, and water [30,36,37]. NEs are thermodynamically unstable, but kinetically stable. MEs are thermodynamically stable systems that form spontaneously and remain stable indefinitely under equilibrium conditions without phase separation, whereas NEs exhibit kinetic stability, being metastable systems that remain stable only due to energy barriers preventing phase separation.

There are varied reports on the sizes of NEs and MEs. From the names, NEs should be in the nano range, and MEs should be in the micro range. However, the globule size of MEs (5–200 nm) [6] is reported to be lower than that of NEs (20–500 nm) [3]. In another report, the size of NEs is reported to be within 100–400 nm, whereas the MEs are within 10–100 nm [68]. Conversely, the globule size of MEs is reported to be higher (~100–400 nm) than the NEs (1 to 100 nm) [69]. The term “micro” for MEs is used due to their small globule size compared to the conventional emulsions. However, their globule size mostly falls in the nanometric range, which creates confusion [69].

MEs are easily prepared by mixing the phases, resulting in spontaneous emulsification without the use of high external energy [60]. In contrast, NEs require both low-energy and high-energy methods for the formulation (see Section 8.1 and Section 8.2). MEs form spontaneously because relatively high surfactant/cosurfactant combinations reduce interfacial tension so effectively that the system minimizes free energy (free energy of formation is negative) and increases entropy, and it drastically reduces the interfacial tension. NEs, lacking such low interfacial energy (free energy of formation is positive), require mechanical input to create and stabilize small globules.

Kinetic stability is governed by the Brownian motion of globules and the prevention of flocculation, sedimentation, and creaming due to energy barriers, whereas thermodynamic stability depends on the equilibrium among the components/phases of the system [69]. In NEs, the free energy of the dispersed droplets is more than that of the separate oil and water phases, making them thermodynamically unstable, whereas in MEs, the dispersed phase has lower free energy than the separate phases, rendering them thermodynamically stable [68,70]. Both MEs and NEs are composed of the same components. Although the compositions of MEs and NEs (oil, water, surfactant, and cosurfactants) are the same, the difference lies in their internal structure, interfacial properties, and formation energetics. MEs contain a higher surfactant-to-oil ratio compared to NEs. MEs are thermodynamically stable systems because of a specific ratio of oil, surfactants, cosurfactants, and water that forms spontaneously when they are mixed in suitable proportions, leading to ultra-low interfacial tension and a negative Gibbs free energy (ΔG < 0). The resulting entropy gain and minimal interfacial energy make the dispersed state an equilibrium condition, ensuring long-term stability. In contrast, NEs are kinetically stable dispersions produced through high external mechanical energy (e.g., ultrasonication, high-pressure homogenization). Although surfactants temporarily prevent droplet coalescence, the higher free energy (ΔG > 0) of NEs and relatively higher interfacial tension make them thermodynamically unstable, and they eventually undergo phase separation via processes like Ostwald ripening or coalescence. Surfactants in the NEs only retard globule coalescence by kinetic stabilization, not by achieving thermodynamic equilibrium. The ultra-small, nanosized NEs offer Brownian motion, which increases repulsion among globules and prevents coalescence, providing kinetic stability with extended resistance against flocculation, sedimentation, and creaming [69]. Thus, MEs are equilibrium systems governed by thermodynamics, whereas NEs are metastable systems stabilized only by kinetic barriers that slow down the destabilization process but do not eliminate the natural tendency toward phase separation [70].

Similar to NEs, MEs are o/w, w/o, and the continuous type [30,36,60]. MEs have been recognized to improve drug permeation through complex ocular barriers [34]. Their smaller globule size improves the permeation across the ocular barriers [6,60]. MEs also inherently possess transparency, fluidity, stability, and load drugs of different polarities; they protect the loaded drug against oxidation; and they enhance ocular permeability and improve ocular bioavailability [30,34,37,71]. Further, they do not need specialized instruments for production and have easy scale-up potential [15]. However, MEs have low viscosity, resulting in low ocular residence. This can be improved with the use of suitable gelling agents or cationic emulsifying agents [15]. Further, the surfactants in MEs may cause ocular irritation and toxicity. Thus, their concentration and type of surfactant should be judiciously selected for the development of ophthalmic MEs. Again, the stability of MEs should be taken into account for their practical application [6].

### 3.3. Macroemulsions

A macroemulsion is a type of emulsion in which the dispersed phase is relatively large, generally greater than 0.4 μm. Due to the large globule size, the macroemulsions are often called opaque or coarse emulsions [68,72]. When used in ophthalmic drug delivery, macroemulsions are an important formulation strategy for increasing the delivery of lipophilic drugs to the eye. O/w-type macroemulsions are typically used for ophthalmic drug delivery, where the active ingredient (mainly hydrophobic) is dispersed in an aqueous solution. W/o-type emulsions are less commonly used but may be applied in some cases. They offer improved ocular bioavailability, sustained release, and prolonged retention. Due to their slower clearance, they can prolong the therapeutic effects of the drug. However, one of the main challenges in using macroemulsions is their physical stability [73]. The large droplets can lead to phase separation over time, requiring proper stabilizers and emulsifying agents to maintain uniformity. In addition, the viscosity of the macroemulsion needs to be carefully controlled to ensure ease of instillation while maintaining drug efficacy. Occasionally, they cause blurry vision due to their larger droplets. Thus, the macroemulsions must be optimized to maintain stability, the droplet size, and compatibility with ocular tissues.

### 3.4. Self-Emulsifying Drug Delivery Systems (SEDDSs)

SEDDSs are widely studied to increase the ocular bioavailability and therapeutic efficacy of drugs used for treating eye diseases. These formulations use a mixture of oils, surfactants, and cosurfactants that, upon contact with aqueous environments (like tears in the eye), spontaneously form emulsions with mild agitation (blinking). The emulsification improves the solubility of lipophilic drugs, increases the residence time, and offers sustained and controlled release in the ocular region. Such formulations are widely exploited for the treatment of glaucoma, AMD, DED, infections, and inflammation. These formulations are broadly classified into (i) self-nanoemulsifying drug delivery systems (SNEDDSs) and (ii) self-microemulsifying drug delivery systems (SMEDDSs) [11,74,75,76].

#### 3.4.1. Self-Nanoemulsifying Drug Delivery Systems (SNEDDSs)

SNEDDSs are an advanced form of SEDDSs. These are anhydrous forms of NEs comprising isotropic mixtures of oil, surfactants, cosurfactants, and/or cosolvents, which emulsify spontaneously to produce o/w NEs upon contact with aqueous physiological body fluids with mild agitation [77,78]. These systems are designed to form nanosized globules upon contact with aqueous fluid (tear), offering several benefits over conventional emulsions, such as improved stability, solubility, and bioavailability. SNEDDSs have been reported for ophthalmic drug delivery. Although NEs are reported with a varied size range of 20–500 nm [3], 100–400 nm [68], or 1–100 nm [69], the sizes of SNEDDSs are reported to be less than 100 nm [79,80]. The nanoemulsion droplets are much smaller than conventional emulsions, providing advantages in drug delivery efficiency [77,80,81,82,83].

#### 3.4.2. Self-Microemulsifying Drug Delivery Systems (SMEDDSs)

SMEDDSs are a subset of SEDDSs, designed to form MEs spontaneously upon contact with the aqueous environment. The reported globule sizes of MEs vary widely, ranging from 5 to 400 nm (10–100 nm [68], 5–200 nm [6], and ~100–400 nm [69]), while SMEDDSs typically exhibit sizes between 100 and 250 nm [79]. These SMEDDSs offer distinct advantages in increasing drug solubility, bioavailability, and the controlled release of drugs. SMEDDSs have shown significant promise in the delivery of ocular drugs, addressing the challenges associated with the topical ophthalmic administration. Upon instillation into the eye, SMEDDSs form microemulsions when they contact with the aqueous environment (tears). The ultra-low-sized droplets improve the solubility of lipophilic drugs and improve their permeation across the ocular barriers, such as the corneal epithelium, conjunctiva, and sclera. The surfactants help stabilize the system and improve the drug’s interaction with the ocular tissues. The small size of the droplets increases the effective surface area for absorption, leading to faster and more efficient penetration into the eye. The sustained release profile permits the drug to stay in the ocular region for longer periods, improving the therapeutic outcome [7,76].

## 4. Emulgels for Ophthalmic Drug Delivery

Emulgels are NDDSs that integrate the advantages of emulsions and gels. The term “emulgel” refers to a gel-based formulation that contains emulsion droplets, typically o/w emulsions. They have received significant interest in the pharmaceutical field, especially in ocular drug delivery, due to their capacity to offer controlled drug release, increased stability, and improved drug absorption. In ophthalmic applications, emulgels provide several advantages for the delivery of both hydrophilic and lipophilic drugs to the eye, overcoming several barriers of conventional eye drop formulations. The components of emulgels include API, oil, surfactant, cosurfactants, water, and gelling agents. The gelling agent imparts viscosity, forming a viscous matrix that enhances the formulation’s ocular residence time. The drug release from the emulgel is affected by the size of the emulsion globule, the type of gelling agent, and viscosity. They have been exploited in the ocular field for glaucoma, DED, AMD, ocular infections, and inflammation. Different mechanisms (electrolyte-triggered, pH-triggered, and temperature-responsive) of in situ ophthalmic emulgels can be formulated with improved therapeutic activity compared to drug-loaded plain gel [15]. Some mucoadhesive gelling agents, such as chitosan, hydroxypropyl methylcellulose or HPMC K4M, Carbopol 981, gellan gum, polyvinylpyrrolidone/PVP K29/32, xanthan gum, sodium alginate, and Poloxamers, have been exploited as mucoadhesive gelling agents for ocular drug delivery to prolong the ocular retention of drugs via various novel drug delivery systems [3,84,85,86,87].

Advantages and disadvantages of emulgels for ophthalmic drug delivery:

- Emulgels can improve the ocular bioavailability of hydrophobic drugs by solubilizing them in the oil phase of the emulsion.
- Due to their gel-like consistency and controlled release characteristics, emulgels improve ocular retention, reducing drug loss due to tear drainage and blink reflexes. This results in better therapeutic outcomes.
- Emulgels allow for prolonged and sustained drug release, reducing frequent dosing and enhancing patient compliance.
- The small emulsion droplet size (nano to micro-scale) of emulgels facilitates better penetration of drugs through the corneal barrier compared to plain gel.
- The use of non-ionic surfactants and gelling agents in emulgels minimizes ocular irritation compared to conventional eye drops that may contain higher concentrations of surfactants. This improves the tolerability of the formulation, particularly for long-term use.
- Emulgels can be used to deliver both hydrophilic and lipophilic drugs. Lipophilic drugs are loaded in the oil phase of the emulsion, while hydrophilic drugs can be included in the aqueous phase. This makes emulgels versatile for various therapeutic applications.

Disadvantages

- Sometimes, emulgels cause ocular irritation due to the existence of surfactants and cosurfactants in the emulsion systems.
- Emulgels with high viscosity may cause blurred vision or a sticky sensation, leading to low patient compliance.
- Due to the sustained-release property of such emulgels, they may delay therapeutic onset when rapid action is required.

### 4.1. Nanoemulgels

The nanoemulgel is an innovative drug delivery system that integrates the advantages of NEs and gels to create a versatile platform for ocular drug delivery. The nanoemulgel system combines the nanosized droplets of an emulsion with the viscoelastic properties of a gel, providing sustained release, enhanced drug penetration, and improved bioavailability of therapeutic agents. This system addresses the challenges of ocular drug delivery, mainly for hydrophobic drugs, and the optimization of controlled drug release. Carbomer, sodium hyaluronate, pluronic F127, hydroxypropyl methylcellulose, chitosan, xanthan gum, gelatin, and polysaccharides have been exploited for the development of ophthalmic nanoemulgels [21,88,89,90].

### 4.2. Microemulgels

The microemulgel is another innovative drug delivery system that integrates the advantages of microemulsions and gels for the effective and sustained delivery of ocular therapeutics. These systems offer enhanced solubility, controlled release, and prolonged ocular retention, which is crucial for improving therapeutic outcomes, particularly in treating ocular diseases. These systems can efficiently deliver both lipophilic and hydrophilic drugs, addressing the challenges associated with conventional ophthalmic formulations [91,92].

### 4.3. In Situ-Forming Nano and Microemulgels

In situ-forming nano and microemulgel systems represent an innovative smart approach in ocular drug delivery, combining the beneficial properties of nanoemulsions or microemulsions with gel-forming excipients that change from a liquid to a gel at the administration site. These systems offer the advantages of sustained drug release, enhanced ocular retention, improved drug penetration, controlled drug delivery, and avoiding frequent administration [6]. The in situ formation of these systems in the ocular environment allows targeted and prolonged ocular drug delivery. The system transforms from liquid to gel under specific conditions due to temperature changes, pH changes, or ionic strength variations.

Different approaches, such as (i) temperature-responsive in situ ophthalmic emulgel, (ii) pH-triggered in situ ophthalmic emulgel, and (iii) electrolyte-triggered in situ emulgel, can be prepared with improved therapeutic activity compared to drug-loaded plain gel [15]. The thermoresponsive materials form a gel at the eye surface (33–34 °C) [93]. They prolong the retention of the drug on the corneal surface and thus improve corneal penetration, thereby avoiding the rapid nasolacrimal drainage of conventional eye drops. They avoid frequent administration, thus improving patient compliance [71]. Thermosensitive gelling agents such as xyloglucan, Poloxamer 407, or Poloxamer 188 form gels at the ocular surface (33–34 °C) [94]. The pH-sensitive gelling agents, such as chitosan and Carbopol-934P, remain in solution at pH 1–6 and gel at the higher ocular pH of 6–7 [15]. The ion-sensitive gelling agents such as gellan gum, xanthan gum, and sodium alginate have also been explored as ion-sensitive gelling agents for in situ-forming gels.

**Figure 2:**
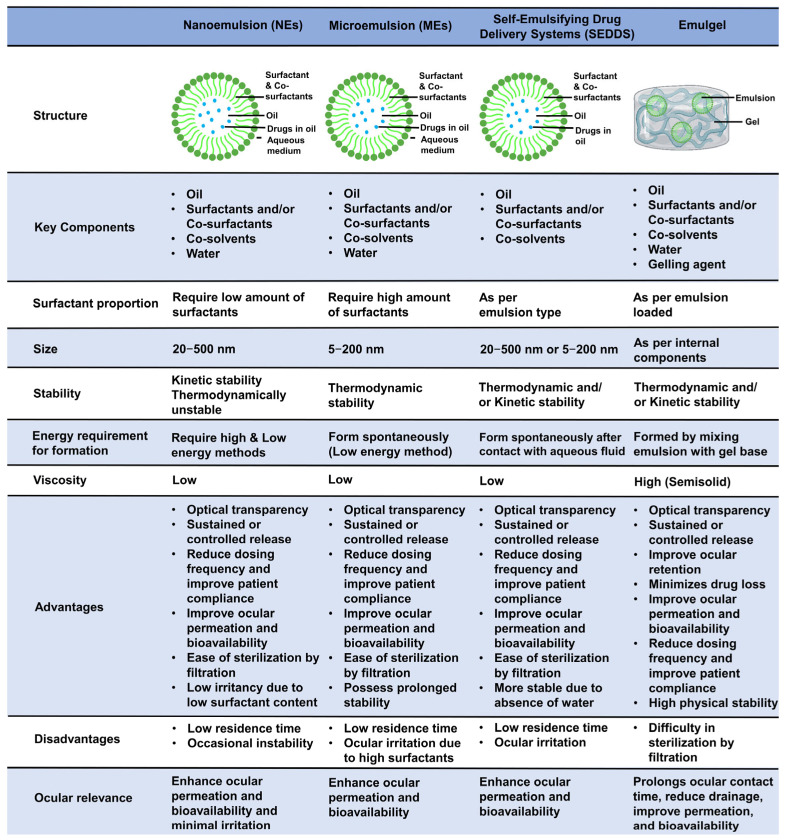
Comparison among ophthalmic nanoemulsions, microemulsions, self-emulsifying drug delivery systems, and emulgels. This figure compares NEs vs. MEs vs. SEDDSs vs. emulgels, highlighting their structure, key components, size, stability, energy requirement for formulation, viscosity, advantages, limitations, and ocular relevance.

A comparison of NEs vs. MEs vs. SEDDSs vs. emulgels, highlighting their structure, key components, size, stability, energy requirement for formulation, viscosity, advantages, limitations, and ocular relevance, is presented in Figure 2.

## 5. Examples of Emulsion Systems for Ophthalmic Drug Delivery

Work to date on ophthalmic emulsion systems for anterior and posterior drug delivery for the treatment of various ocular diseases is listed in Table 1.

## 6. Mechanism Behind Improved Ocular Bioavailability by Ophthalmic Emulsions

**Figure 3:**
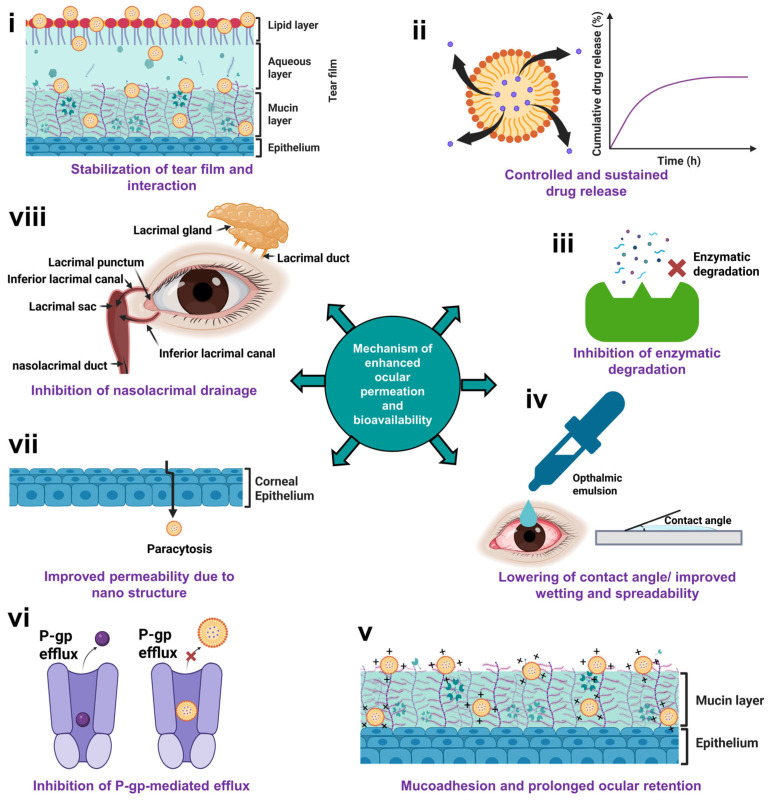
Mechanism of improved ocular bioavailability of nano/microemulsions and emulgels across ocular barriers. This figure illustrates the key mechanisms of enhanced permeation and ocular bioavailability of emulsion and emulgel-based ophthalmic drug delivery systems via (**i**) stabilization of tear film and interaction, (**ii**) controlled and sustained drug release, (**iii**) inhibition of enzymatic degradation, (**iv**) lowering of contact angle/improved wetting and spreadability, (**v**) mucoadhesion and prolonged ocular retention due to electrostatic interaction between by cationic emulsion and anionic mucin layer, (**vi**) inhibition of P-gp mediated efflux, (**vii**) paracytosis due to nanostructure, and (**viii**) inhibition of nasolacrimal drainage.

The mechanisms enhancing ocular bioavailability of nano/microemulsions and emulgels across ocular barriers is summarized in Figure 3. Various mechanisms responsible for improved ocular bioavailability of emulsions include (i) stabilization of tear film and interaction, (ii) controlled and sustained drug release, (iii) inhibition of enzymatic degradation, (iv) lowering of contact angle/improved wetting and spreadability, (v) mucoadhesion and prolonged ocular retention, (vi) inhibition of P-gp mediated efflux, (vii) paracytosis due to the nanostructure, and (viii) inhibition of nasolacrimal drainage.

### 6.1. Stabilization of Tear Film and Interaction

The three layers of the tear film are the inner mucin layer, middle aqueous layer, and outer lipid layer. The outer lipid layer prevents the loss of aqueous fluid by evaporation, maintains the surface tension, and causes lubrication of the eyeball. The mucin layer changes the epithelium of cornea from lipophilic to hydrophilic. The biophysical interaction of emulsions with the tear film and mucin is crucial for prolonged ocular retention and ocular bioavailability. Formulation properties, such as globule size, viscosity, surface charge, and presence of mucoadhesive excipients, influence the biophysical interaction. Ophthalmic emulsions interact with the tear film, spread the drop, prolong the ocular retention time, and ultimately improve the ocular bioavailability [3,4]. The lipid excipients of ophthalmic emulsions interact with the lipid-rich layer of the tear film, which improves the retention of the ophthalmic formulation in the conjunctival sac and acts as a drug depot [3]. The oil phase of the emulsion lipid layer of the tear prevents the evaporation of the aqueous layer, whereas the aqueous phase of the emulsion increases the aqueous volume of the tear and moistens the eye. The surfactants and cosurfactants increase the wettability of the tear film by interacting with the mucin layer [4]. This property of o/w emulsions is helpful for dry eye syndrome [3].

### 6.2. Controlled and Sustained Drug Release

Entrapment of the drug in the oil droplets of NEs or MEs allows slow diffusion, thereby preventing the “burst release” or pulsatile release observed with conventional eye drops while reducing the dosing frequency and maintaining desired therapeutic levels [17,111].

### 6.3. Inhibition of Enzymatic Degradation

As discussed earlier, the tear film and ocular tissues (cornea, ciliary body, iris, lens, and retina) contain various metabolizing enzymes (esterase, cytochrome P-450, peptidases, aldehyde oxidase, cyclooxygenase, hydrolase, aldo/ketone reductase, monoamine oxidase, and transferase) that degrade active drugs, thereby decreasing ocular bioavailability [57]. The loading of drugs into NEs or MEs prevents direct exposure to these enzymes and shields them from enzymatic degradation, ensuring that more intact drug reaches ocular tissues [124,125].

### 6.4. Lowering of Contact Angle or Improved Wetting and Spreadability

Emulsions decrease the contact angle between formulation and cornea, increase wetting, and offer prolonged retention of the formulation on the eye [3,35]. Cationic NEs or MEs further lower the contact angle and prolong the drug residence time via electrostatic interactions with the anionic mucin layer of the cornea [3]. Compared to the w/o type of emulsions, the o/w emulsions are ideal for the ocular delivery of hydrophobic drugs as the continuous phase is water, which is diluted easily with aqueous tear fluid of the eye, enabling excellent spreadability, wetting, and improved ocular permeation [35].

### 6.5. Mucoadhesion

Mucoadhesion allows the enhanced ocular retention of formulations, thereby allowing the exposure of medicament to the ocular surface [46]. Ocular mucus comprises transmembrane mucins (MUC1, MUC4, MUC13, MUC15-17) and secretory mucins (MUC2, MUC5AC, MUC7). These mucins modulate the viscoelasticity and surface tension of the tear film and increase the wetting of the ocular surface glycocalyx [46]. Mucoadhesion occurs via hydrophobic bonds, Van der Waals interactions, hydrogen bonds, ionic bonds, and covalent bonds [46]. Factors such as the concentration, hydration properties, molecular weight, chain length, degree of cross-linking, charge, spatial conformations, flexibility, viscosity, and pH of polymers affect the degree of mucoadhesion [46].

### 6.6. Inhibition of P-gp-Mediated Efflux

The corneal epithelium contains the P-gp efflux pump, which inhibits the entry of outside substances by an efflux mechanism and thus decreases the permeability of many topical drugs. The surfactants of the emulsion inhibit the P-gp-mediated efflux and improve the drug permeation [3]. Non-ionic surfactants are reported extensively for inhibition of P-gp efflux, thereby increasing tissue absorption and bioavailability [126].

### 6.7. Improved Permeability Due to Nanostructure

The smaller globule size of NEs and MEs increases the surface area-to-volume ratio, which improves drug contact with the corneal surface [127]. The small globule size facilitates high permeation across the corneal tight junctions, thus improving therapeutic efficacy [128]. Further, the surfactants and cosurfactants in such formulations act as permeation enhancers, which reduce corneal epithelial barrier resistance by loosening tight junctions and altering permeability [129].

### 6.8. Inhibition of Nasolacrimal Drainage

The presence of oil, surfactants, mucoadhesive agents, and gelling agents in MEs, NEs, and gels increases viscosity and promotes adhesion to the mucin layer, thereby decreasing the flow of drugs into the nasolacrimal duct and loss. The nanometric globules form a stable film by spreading on the corneal surface uniformly, which reduces the rapid nasolacrimal drainage [17,85,130].

## 7. Drug Absorption Pathways of Topically Administered Ophthalmic Emulsion and Emulgel to the Posterior Segment

**Figure 4:**
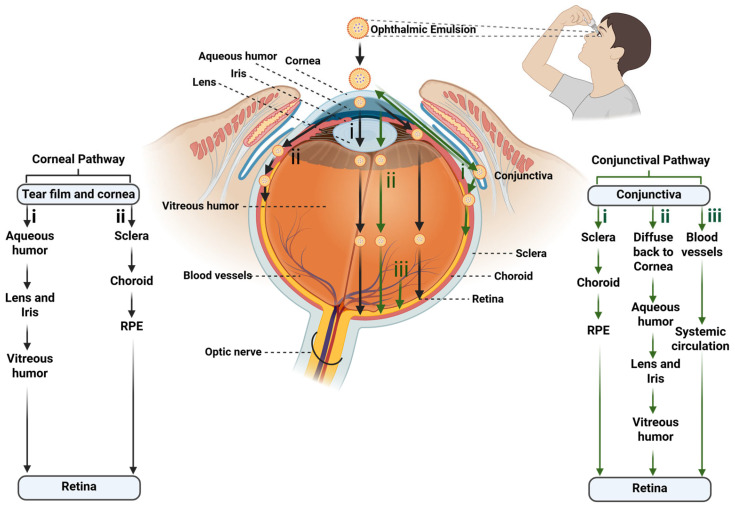
Drug permeation pathways of topically administered ophthalmic emulsions to the posterior eye segment. This schematic figure illustrates the major ocular pathways through which drug-loaded ophthalmic emulsions can penetrate ocular tissues following topical administration. The figure highlights the corneal pathway, involving permeation across the tear film and corneal epithelium, followed by (i) aqueous humor, lens and iris, and vitreous to reach the retina, and/or (ii) sclera, choroid, and retinal pigment epithelium (RPE) to reach the posterior retina. It also highlights the conjunctival pathway, where the emulsion globules (i) diffuse through the conjunctiva and pass through the sclera, choroid, and RPE, and/or (ii) diffuse back to cornea, pass through the aqueous humor, lens and iris, and vitreous humor, and/or (iii) diffuse through the blood vessels and systemic circulation to access the posterior segment. The black colored arrows represent corneal pathway, whereas the green colored arrows represent conjunctival pathway.

In general, the topically administered ophthalmic emulsion follows (a) the corneal and (b) conjunctival pathway (Figure 4). Among them, the corneal pathway has been identified as the main pathway for the ocular absorption of hydrophobic drugs to the posterior ocular segment [46].

Ophthalmic NEs/MEs/SNEDDSs/SMEDDSs/emulgels cross the cornea via the paracellular route. After corneal absorption, the drugs reach the posterior segment of the eye by following either of the two pathways. (i) After corneal permeation, 90% of the absorbed molecules are distributed to the anterior chamber of the eye. Then, they redistribute to the nearby ocular tissues, such as the lens, ciliary body/iris, and vitreous chamber, and ultimately, reach the posterior retina. (ii) The drugs absorbed across the cornea can also diffuse to the sclera, from where they are distributed to the choroid and the posterior segment of ocular tissues [3,46].

Via the conjunctival pathway, the anterior chamber of the eye is bypassed, and drug distribution mainly occurs in the vitreous humor and uveal tract. By this pathway, the drugs reach the posterior segment of the eye through one of three pathways. (i) The major pathway is the diffusion of the drug across the conjunctiva, sclera, and choroid to reach the retina. (ii) Occasionally, the drugs absorbed by the conjunctival pathway can also diffuse laterally to the cornea, iris, and ciliary body, i.e., diffuse to the anterior chamber with other intraocular tissue. (iii) Lastly, the blood vessels at the conjunctiva can absorb the drug into the systemic circulation, followed by distribution to various organs of the body, including retinal tissue [46].

## 8. Formulation Strategies of Ophthalmic Emulsions and Emulgels

Formulation of emulsions occurs by high-energy and low-energy methods [4,95]. The high-energy methods require high-shear stirring, ultrasonication, or high-shear homogenization for the development of emulsions [16]. Low-energy methods rely on the intrinsic physicochemical properties of the system rather than strong external mechanical energy, with only minimal external energy applied. These methods involve changes in temperature and chemical composition for the formulation of emulsions. Such methods include the phase inversion composition [16], the phase inversion temperature method, and the spontaneous emulsification method [95]. The low-energy methods possess the advantages of (i) utilization of internal energy/requiring gentle stirring, (ii) a smaller globule size compared to high-energy methods, (iii) ease of scale-up, (iv) no specialized equipment, (v) avoiding the degradation of thermolabile drugs due to heating (mainly induced by high-energy methods), and (vi) a low production cost. However, the low-energy methods require a high concentration of surfactants [95]. Suitable gelling agents are incorporated in the aqueous phase to develop ophthalmic emulgels. Various stimuli-responsive gelling agents (thermosensitive, pH-sensitive, ion-sensitive) are used to develop in situ ophthalmic gels. The emulsion and aqueous gel base are prepared separately and finally incorporated into an emulsion to produce emulgel [12,131,132].

### 8.1. High-Energy Methods

The application of high energy for a short duration causes cleavage of the internal dispersed phase in the continuous/dispersion medium to develop nanometric/micrometric emulsions. High-pressure homogenizations, high-shear stirring, ultrasonication, magnetic stirring, and microfluidics are mostly used as external energy sources [4,16].

#### 8.1.1. Homogenization/High-Pressure Homogenization and High-Speed Homogenization

In this technique, macroemulsions are forcefully passed through a small orifice at very high pressure (500 to 5000 psi) to produce NEs or MEs. The intense turbulence, hydraulic shear, and cavitation during homogenization cause the formation of nano- or microglobules [3,4,16,105]. The homogenization pressure and cycles influence the properties of ocular NEs, such as globule size, surface charge, and polydispersity index, which ultimately affect the formulation stability [105]. In general, a higher homogenization pressure and more processing cycles improve the shear and cavitation forces acting on the emulsion droplets, which decreases the globule size and narrows the PDI, thereby improving the homogeneity and stability of the formulation [133].

#### 8.1.2. High-Shear Stirring

This uses high-energy rotor-stators and mixers to develop nanometric globules. Silverson mixers, rotors, and stators are mostly used to emulsify oil with the water phase. The high speed induces high refraction, and the centrifugal force causes the passage of the globules through tiny orifices to develop emulsions [16].

#### 8.1.3. Ultrasonication

During ultrasonication, the acoustic field produces an interfacial wave that causes the dispersion of the internal phase in the external phase. Moreover, the ultrasound causes acoustic cavitation, leading to the development and collapse of microbubbles due to variations in pressure caused by the sound wave. The generation of localized high turbulence and micro-implosions leads to the disruption of larger globules into sub-micron or nanometric size [3,4,16]. This method is widely employed for laboratory-scale development of NEs [4].

#### 8.1.4. Magnetic Stirring

This process involves the addition of the internal oily phase/organic phase into the aqueous medium with continuous magnetic stirring. Since the technique does not develop heat, as in the case of ultrasonication, it is suitable for thermolabile drugs [16]. The size of the globules depends on the rate of stirring. The nanometric globule size forms at a very high speed.

#### 8.1.5. Microfluidic Method

This technique is widely utilized in the pharmaceutical industry to develop fine emulsions. In this technique, a microfluidizer is employed to induce high pressures, which propel macroemulsions to an interaction chamber through micro-channels at high pressure (500–20,000 psi) to produce NEs. Emulsions with the desired size can be developed by changing the operating pressure, shearing, impact, and cavitation [3,4,16].

### 8.2. Low-Energy Methods

Low-energy methods require very low energy for the development of NEs or MEs. Such techniques involve spontaneous emulsification, the phase inversion temperature, emulsion inversion point, and phase inversion composition [3].

#### 8.2.1. Spontaneous Emulsification

This method forms the NEs or MEs spontaneously at room temperature without using high external energy. This method is also called solvent diffusion emulsification or the self-emulsification method. When the internal dispersed phase is combined with the dispersion medium/continuous phase containing a high proportion of surfactants/cosurfactants, it undergoes spontaneous emulsification by self-assembly. SEDDSs, SMEDDSs, and SNEDDSs are developed mainly by this technique upon mild agitation by blinking of the eye [3,16].

#### 8.2.2. Phase Inversion Temperature Method

In this method, a change in temperature causes the phase inversion. Certain surfactants, such as polyethoxylated surfactants, possess temperature-dependent solubility and hydrophilic–lipophilic balance (HLB) values. At higher temperatures, such surfactants undergo changes into a hydrophobic form due to the dehydration of polyoxyethylene groups. In this condition, the o/w type of NE changes to the w/o type. The extremely low interfacial tension at the HLB temperature promotes spontaneous emulsification. However, the developed emulsions show rapid coalescence, which leads to instability [3,4,16].

#### 8.2.3. Emulsion-Phase Inversion Method or Emulsion Inversion Point

Phase inversion usually occurs when the concentration of the dispersed phase is relatively higher than that of the dispersion medium. In such conditions, the dispersed globules are closely packed, leading to the translation of o/w emulsions into w/o emulsions or conversely [95]. The phase transition happens throughout the emulsification process. Temperature, salt concentration, oil fraction, water, input energy, and variations in formulation parameters (salinity) affect the phase inversion. These techniques utilize chemical energy released during phase transformation during emulsification [134].

#### 8.2.4. Phase Inversion Composition Method

In this technique, a change in the composition of the phases leads to phase inversion. At a constant temperature, a change in the HLB occurs due to the addition of a new component. When the dispersed phase is continuously mixed with dispersion medium and surfactant/cosurfactants, NEs or MEs are formed. Due to its simplicity, in addition to being one of the components, it is mostly used for the large-scale formulation of NEs/MEs. However, this method requires the precise selection of HLB-transforming agents for the development of the best NEs [3,4,16].

### 8.3. Other Methods

Some of the other techniques, such as the bubble-bursting method and evaporative ripening methods, are used for the development of NEs or MEs. The bubble-bursting method involves the generation of bubbles at the oil–water interface, then causing them to burst, which causes the dispersion of oil as tiny droplets in the aqueous continuous phase, effectively producing NEs. The energy released during the collapse of the bubble causes the breaking of oil globules into submicron size, and the surfactants stabilize the droplets in the aqueous external phase [16]. The evaporative ripening method includes the use of volatile and non-volatile oils as an internal phase. They are prepared by conventional techniques and heated to evaporate the volatile oil, which shrinks the globule size [16].

## 9. Approaches for Selection of Excipients for Ophthalmic Emulsions and Emulgels

The selection of appropriate excipients is a critical step in the development of safe and effective ophthalmic emulsions and emulgels, as each component significantly influences the therapeutic activity, stability, and patient acceptability. Key decision criteria for excipient selection include ocular biocompatibility, solubility of the drug, physicochemical properties of oil, surfactants, and cosurfactants (refractive index, viscosity, therapeutic profile of the oil phase, origin and type of excipients, safety and toxicity profile, HLB value, critical micelle concentration (CMC)), properties of the gelling agent, preservatives and their toxicity considerations, stability, and relevant regulatory aspects.

### 9.1. Ocular Biocompatibility

Generally recognized as safe (GRAS) listed excipients should be considered for the development of ophthalmic emulsions and emulgels, as the ocular tissue is very sensitive [3]. These excipients should be biocompatible, nontoxic, and nonirritant to the eye. Since excipients (oil, surfactants, cosurfactants, cosolvents, and preservatives) can induce irritation, selecting appropriate types and concentrations is crucial. Their ocular safety should be confirmed through in vitro, ex vivo, and in vivo evaluations. A wide range of excipients are currently employed at the laboratory scale for developing ophthalmic emulsions; however, many of these are not listed as GRAS.

### 9.2. Solubility of Drug in Excipients

The drug should be soluble in the emulsion components. The selection of suitable oil, surfactants, cosurfactants, and cosolvents is mainly based on the saturation solubility study of drugs. Safe excipients with a high drug solubilization capacity are selected to develop a pseudoternary phase diagram and formulation.

### 9.3. Physicochemical Properties, Therapeutic Profile, Origin, Types, and Safety Profile of Oil Phase

The physicochemical properties (such as refractive index, viscosity, and drug solubility), along with the therapeutic properties, origin, type, and safety profile of the oil phase, significantly influence the patient compatibility, safety, and therapeutic efficacy of ophthalmic emulsions.

The oily phase includes vegetable oil, mineral oil, medium-chain triglycerides, and others [4]. The long-chain and medium-chain triglycerides are mainly used as a lipid phase in NEs to improve the solubility and ocular bioavailability of lipophilic drugs [4]. The following oils have been reported for ocular emulsions: Capryol^®^ 90, Capryol^®^ PGMC, cinnamon oil, peanut oil, olive oil, castor oil, olive oil, soya oil, corn oil, pegylated castor oil (polyoxyethylene 35 castor oil), rose oil, lemon oil, grape seed oil, liquid paraffin, isopropyl myristate (IPM), pomegranate seed oil, colchicum oil, chamomile oil, egg-lecithin, triacetin, pegylated castor oil, Lipoid E80, Lipoid S75, Lipoid S100, Lipoid E-80, Phospholipon^®^ 90H, oleic acid, Dermol^®^ M5, vitamin E, ethyl oleate, Mygliol^®^ 812, Mygliol^®^ 840, Estasan (caprylic- capric-triglyceride), Capmul^®^ MCM, Epikuron^®^ 200, Maisine^®^ 35-1, Lauroglycol^®^ 90, and 1,2-dioleoyl-3-trimethylammonium propane (DOTAP) [3].

#### 9.3.1. Refractive Index

A refractive index (RI) close to that of water (1.333) indicates high transparency of ocular emulsions, which is essential for clear vision without blurring after application. Since tear fluid RI varies between 1.340 and 1.360, an oil phase with RI proximity to the aqueous phase should be selected. Formulations with an RI close to the RI of tear fluid are preferred to avoid vision discomfort or blurring [3,17].

#### 9.3.2. Viscosity

Formulations with very low viscosity tend to spill easily and exhibit poor ocular retention, whereas excessively high viscosity can hinder topical administration. Further, oils with low viscosity and interfacial tension provide emulsions with smaller globules [4]. Hence, an optimum viscosity is needed for ophthalmic formulations. The concentration and type of oil phase should be selected in such a way that it should offer a final viscosity of the formulation within the acceptable range (15–150 mPa·s) [16]. Oils with low surface tension and viscosity are essential to develop NEs with a smaller globule size [18].

#### 9.3.3. Solubility of Drug

The drug’s solubility in the oil phase is also an important factor to consider for the development of ophthalmic emulsions. The oil should solubilize the required amount of drug to achieve therapeutic efficacy. Lipophilic drugs can be loaded into the oil phase with a low amount of surfactants or cosurfactants to avoid toxicity issues of surfactants. Single oil or a mixture of oils can be used for the solubilization of drugs [18]. An oil phase within a concentration of 5–20% is mostly used for the development of ophthalmic emulsions [18].

#### 9.3.4. Therapeutic Profile of Oil

The therapeutic profile of the oil phase is important since oils possess various therapeutic properties, including anti-inflammatory (e.g., wild olive oil), antibacterial (e.g., almond oil, essential oils of *Mentha piperita*, *Rosmarinus officinalis*, *Melaleuca alternifolia*, *Thymus vulgaris*), and antioxidant activities (e.g., wild olive oil, almond oil) [18,135]. Oils reported for the treatment of various ocular disorders may be selected for an oil phase that can provide additional therapeutic benefits.

#### 9.3.5. Origin of Oil

The origin of the oil phase is also crucial for choosing the oil phase. Both natural oils (e.g., olive oil, castor oil, almond oil) and synthetic oils (e.g., propylene glycol dicaprylate, dicaprylate) are used for the development of ophthalmic emulsions. In addition to these, synthetic phospholipids (e.g., 1,2-dimyristoyl-sn-glycero-3-phosphocholine/DMPC) are often employed as the oil phase for the formulation of ophthalmic emulsions [18].

#### 9.3.6. Safety and Biocompatibility Profile of Oil

The safety and biocompatibility profile of the oil phase should be considered for use as the oil phase of ophthalmic emulsions. Vegetable oils are noted for their toxicity to the cornea and conjunctiva [18]. Sometimes, the rancidity of the oil phase causes the development of harmful free radicals that further damage ocular tissue [18].

### 9.4. Physicochemical Properties, Types, and Toxicity of Surfactants and Cosurfactants

Surfactants decrease the interfacial tension between the water and oil interface and produce emulsions [4]. Due to their amphiphilic properties, they create a layer on the oil phase, inhibiting the oil globules from flocculation, Ostwald ripening, coalescence, and phase separation, leading to prolonged stability [4]. They also solubilize both hydrophobic and hydrophilic drugs. Hence, surfactants act as emulsifiers, solubilizers, and stabilizers [18]. Types of surfactant, HLB value, viscosity, solubilization ability for drugs, toxicity, and regulatory aspects of surfactants and cosurfactants should be considered for the development of ophthalmic emulsions [18].

The following surfactants have been used for ocular emulsions: Caproyl™ PGMC, Cremophor^®^ EL, Solutol HS15, Kolliphor HS15, Cremophor EL35, Cremophor^®^ RH 40, Cremophor^®^ RH 60, Plantacare^®^ 818 UP, Plantacare^®^ 1200 UP, Pluronic F127, Pluronic F68, Eumulgin^®^ CO40, Acrysol™ K-140, Acrysol™ K-140, Tween^®^ 80, Tween^®^ 40, Tween^®^ 20, Span^®^ 20, cetrimonium bromide, Brij 35, D-α-tocopherol polyethylene glycol succinate (TPGS), Tyloxapol, Poloxamer 407, Poloxamer188, Miranol C2M, Soluphor P, cetalkonium chloride, and DSPE-PEG 2000 [3,4].

Cosurfactants are low-molecular-weight amphiphilic structures similar to surfactants. They provide synergistic action with surfactants by enhancing the flexibility and fluidity of the interfacial layer around the oil globules [18]. The cosurfactants reduce interfacial tension, increase interfacial fluidity, enhance system entropy, and maintain film curvature and the optimum viscosity and transparency of emulsions. They are used with surfactants at a particular ratio to obtain globules with the optimum size [3]. Sometimes, cosolvents are used as cosurfactants; hence, they are used alone or in combination with other cosurfactants. The cosolvents offer increased drug solubility, globule dispersion, and stability to the emulsions. The cosurfactants improve the fluidity of the interfacial layer of droplets and reduce the amount of surfactants required, thus avoiding their possible toxicity [4,18]. Cosurfactants should be compatible and synergize with the primary surfactant to produce stable emulsions with reduced interfacial tension. They increase the stability region of NEs in the phase diagrams. Suitable proportions of surfactants and cosurfactants are required to develop emulsions with a smaller globule size [18].

The following cosurfactants have been reported for ocular emulsions: Propylene glycol, Transcutol^®^ P, Cremophor^®^ EL, Transcutol^®^ HP, ethylene glycol, pentylene glycol, butylene glycol, hexylene glycol, polyethylene glycol (PEG)-300, polyethylene glycol (PEG)-600, polyethylene glycol (PEG)-400, Plurol^®^ Oleique, and glycerol [3,4].

#### 9.4.1. HLB Value of Surfactants

Surfactants with high HLB values (8–18) are used for o/w emulsions, whereas surfactants with low HLB values (3–6) are used for w/o emulsions [18]. Surfactants with HLB values of more than 10 facilitate corneal permeation; hence, they are used for o/w emulsions [3]. Emulsions often use a combination of surfactants whose composition is decided by HLB calculation to provide a stable formulation [18].

#### 9.4.2. Type of Surfactants

Based on the electrical properties, surfactants are classified into non-ionic, anionic, cationic, and zwitterionic/amphoteric. Ionic surfactants offer colloidal stability by offering a high zeta potential due to surface charge, whereas non-ionic surfactants offer stability due to steric hindrance, hydrogen bonding, and dipole interaction. The ionic surfactants cause electrostatic repulsion among the droplets, prevent aggregation/coalescence of globules, and offer stability [4]. Combining cationic surfactants with non-ionic surfactants is reported to improve colloidal stability [4]. The cationic quaternary ammonium compounds act as surfactants and preservatives [4]. Non-ionic surfactants are considered safer than ionic surfactants [3,4]. The irritancy of surfactants is in the order of non-ionic < amphoteric < anionic < cationic [18]. Various non-ionic surfactants (e.g., Tween^®^ 20, Tween^®^ 80, Cremophor^®^ EL, Poloxamer 188, Cremophor^®^ RH40) are often used in various ophthalmic emulsions [18]. The hydrophilic non-ionic surfactant, Tween^®^ 80, is extensively used in ocular emulsions since it is non-irritant, has excellent ocular biocompatibility, and causes reversible alterations in the ocular surface permeability [4]. Cationic surfactants are often employed to prolong ocular retention. The positive charge on the cationic surfactants offers a higher affinity towards negatively charged mucin at the ocular surface, leading to improved retention and bioavailability [18]. Some cationic surfactants, such as benzalkonium chloride and cetalkonium chloride, also act as antimicrobial preservatives [18]. Cationic surfactants are frequently mixed with non-ionic surfactants to provide improved colloidal stability [18]. Cationic lipids, such as 1,2-dioleoyl-3-trimethylammonium-propane (DOTAP) or dioleoyl phosphatidylethanolamine (DOPE), are often used in non-preserved cationic ophthalmic formulations [18].

#### 9.4.3. Concentration of Surfactants

A higher proportion (20–50%) of surfactants compared to oil is required for a stable emulsion. However, surfactants at very high concentrations cause ocular irritation and produce emulsions with high viscosity [3,4]. The higher viscosity of the topical ophthalmic formulation helps increase ocular retention, thereby improving the absorption and therapeutic efficacy of drugs. However, increased viscosity can also cause temporary visual blur after instillation. Very high concentrations of surfactant disturb the tear film and damage ocular tissue [18].

#### 9.4.4. Solubility of Drugs in Surfactants

In addition to the solubility of a drug in the oil phase, its solubility in the surfactant also plays a vital role in achieving maximum drug loading. Surfactants can improve the solubilization of lipophilic drugs either below the CMC by creating localized microenvironments that attract drug molecules or above the CMC through micellar solubilization. Surfactants with low CMC values are particularly advantageous, as they enable effective micellar solubilization at the low concentrations typically required for ophthalmic emulsions [18].

#### 9.4.5. Biocompatibility of Surfactants

Biocompatible surfactants with low ocular toxicity and irritation potential are preferred for the formulation of ophthalmic emulsions. Irritation due to surfactants and cosurfactants may be avoided by lowering their concentrations [18].

### 9.5. Properties of Gelling Agents

The selection of a suitable gelling agent is critical in the development of ocular emulgels, as it determines the viscosity, ocular retention, and in situ gelation behavior. An ideal gelling agent should provide optimum viscosity to minimize ocular drainage while ensuring patient comfort and ease of installation.

#### 9.5.1. Stimuli-Responsive Gelling Agent

Stimuli-responsive polymers (pH-sensitive, thermosensitive, ion-sensitive) can be used as gelling agents to produce in situ gels after topical ocular application.

The pH-sensitive polymers (e.g., chitosan and carbopol) are liquid at an acidic pH (pH 1–6) and converted to gel (sol–gel conversion) at a relatively higher neutral ocular pH. They are used when pH-triggered sol–gel transition is desired.

Thermoresponsive polymers (e.g., Poloxamers: Poloxamer 407, Poloxamer 188, xyloglucan, glycerol-2-phosphate, and poly (N-isopropyl acrylamide)) convert to gel at an ocular surface temperature (33–34 °C). Such gelling agents are preferred when temperature-induced gelation is desired.

Ion-sensitive polymers (e.g., gellan gum, sodium alginate, xanthan gum) are ideal when ionic gelation is targeted. The sol-gel transformation for ion-responsive polymers occurs at the ocular surface due to various cations (e.g., magnesium, sodium, calcium) and anions (e.g., bicarbonate, chloride, phosphates) in tear fluids. The ionic interaction between these ions and polymers leads to the formation of ionic bonds and the development of “egg box” structures [3].

#### 9.5.2. Mucoadhesive Property

Mucoadhesive polymers, such as chitosan, carbopol 981, sodium alginate, polyvinylpyrrolidone, and hydroxypropyl methylcellulose (HPMC or hypromellose), are used as mucoadhesive agents due to their excellent aqueous solubility, transparency, biocompatibility, and rheological properties [46,84]. The structure of mucus allows for interaction with mucoadhesive polymers containing nanoformulations. The mucus layer of ocular tissue has been widely studied for the non-invasive delivery of drugs to the anterior and posterior eye segments [46].

### 9.6. Type of Preservatives and Toxicity Considerations

To avoid microbial contamination during use, ophthalmic emulsions and emulgels often require preservatives. Cationic preservatives, like benzalkonium chloride, cetalkonium chloride, cetyl trimethyl ammonium bromide (CTAB)/cetrimide, and benzethonium chloride, are used in the ophthalmic NEs and MEs. The cationic preservatives also act as surfactants. In addition to these, other cationic agents, such as stearylamine, oleylamine, chitosan, poly(L-lysine), and poly(ethylenimine), are reported to be used in the development of emulsions. These cationic agents offer electrostatic repulsion among the globules and offer enhanced stability. Further, these cationic agents interact with anionic mucin on the ocular surface, which offers prolonged retention and improved therapeutic benefits [3,108].

However, preservatives are reported to induce conjunctival toxicity, tear film disruption, allergic symptoms, and corneal damage. They disrupt the glycocalyx and lead to ocular surface inflammation, allergy, fibrosis, and DED. Thus, the newer, less-toxic preservatives (polyquaternium-1, polixetonium, sorbic acid, sodium perborate) may be considered to reduce ocular toxicity [136]. Preservative-free formulations are now being developed to avoid preservative-related irritation.

### 9.7. Safety Profile, Concentration, and Type of Osmotic Agents

Osmotic agents maintain the osmolarity of topical ocular formulations [4]. Osmotic agents, such as propylene glycol, glycerol, sorbitol, mannitol, and dextrose, are used in ophthalmic emulsions to make them isotonic and suitable for ophthalmic use [3]. Among them, glycerol is widely used as an osmotic agent and cosolvent in NEs and can potentially alter the viscosity, density, and refractive index of the formulation [4]. The decrease in the globule size has been reported when increasing the amount of glycerol in the aqueous phase of o/w NEs and vice versa [4]. The optimum concentration of osmotic agents should be selected to achieve osmolarity similar to tear fluid. Non-toxic, non-irritant, and compatible osmotic agents should be considered for the development of ophthalmic emulsions.

### 9.8. Properties of Other Excipients

Stabilizers in the emulsion improve the stability [134]. The selection of stabilizers depends on their compatibility with formulation components and suitability for ocular use (biocompatible, non-irritant, and GRAS-listed). Texture modifiers (xanthan gum) mostly increase the viscosity and prevent the gravitational phase separation. They should be chosen based on their capacity to achieve optimum viscosity, physical stability, and ocular retention without causing discomfort and blurring. Ripening inhibitors such as hydrophobic compounds and long-chain triglycerides are used to prevent Ostwald ripening. Antioxidants (ascorbic acid, tocopherols, and butylated hydroxytoluene (BHT)) are mostly used to prevent the oxidation of the oil phase and maintain the stability of emulsions. Ripening agents and antioxidants should be chosen based on their safety, efficacy, compatibility, and stability under the storage conditions of formulations.

### 9.9. Volume of Dispersion Medium or Continuous Phase

Water or phosphate-buffered saline (PBS) is mostly used as a dispersion medium. The pH of the aqueous phase affects the phase behavior of the emulsion [137]. Buffers are mainly used as pH modifiers to make formulations compatible with ocular fluid. Different components of emulsions, such as surfactants, osmotic agents, or polymers of aqueous phase, provide a supplementary effect on the eye surface [137]. A higher volume of aqueous phase may lead to phase separation and cracking of emulsions. An appropriate proportion of aqueous phase should be considered from the ternary-phase diagrams for the development of stable emulsions.

### 9.10. Stability

The refractive index, viscosity, interfacial tension, polarity, and density of the oil phase affect the stability of emulsions [3,4]. Physicochemical properties and types of surfactants and cosurfactants affect the stability of the emulsion. A relatively higher proportion of surfactants and a lower proportion of oil phase constitute emulsions with prolonged stability. The surface charge of surfactants affects zeta potential and stability. Ionic surfactants provide colloidal stability by generating a high zeta potential through surface charge, leading to electrostatic repulsion that prevents droplet aggregation or coalescence. In contrast, non-ionic surfactants stabilize emulsions through steric hindrance, hydrogen bonding, and dipole interactions, maintaining the uniformity and stability of the formulation [4]. Loading a low quantity of drug provides better formulation stability, whereas high drug loading can lead to precipitation and phase separation, compromising the integrity of the emulsion.

### 9.11. Regulatory Considerations

Regulatory approval and safety of the excipients are crucial during the development of ophthalmic products [2]. Regulatory requirements for ophthalmic emulsions focus on safety, stability, sterility, purity, and adherence to international and pharmacopeial standards. Excipients intended for topical ophthalmic use must either have prior approval for ophthalmic applications or be supported by ocular toxicity data. They should be non-irritating, non-toxic, sterile, and either previously used in approved ophthalmic emulsions or listed in the FDA Inactive Ingredient Database or documented as GRAS (Generally Recognized as Safe).

## 10. Design of Experiment Considerations for Optimization of Ophthalmic Emulsions and Emulgels

**Figure 5:**
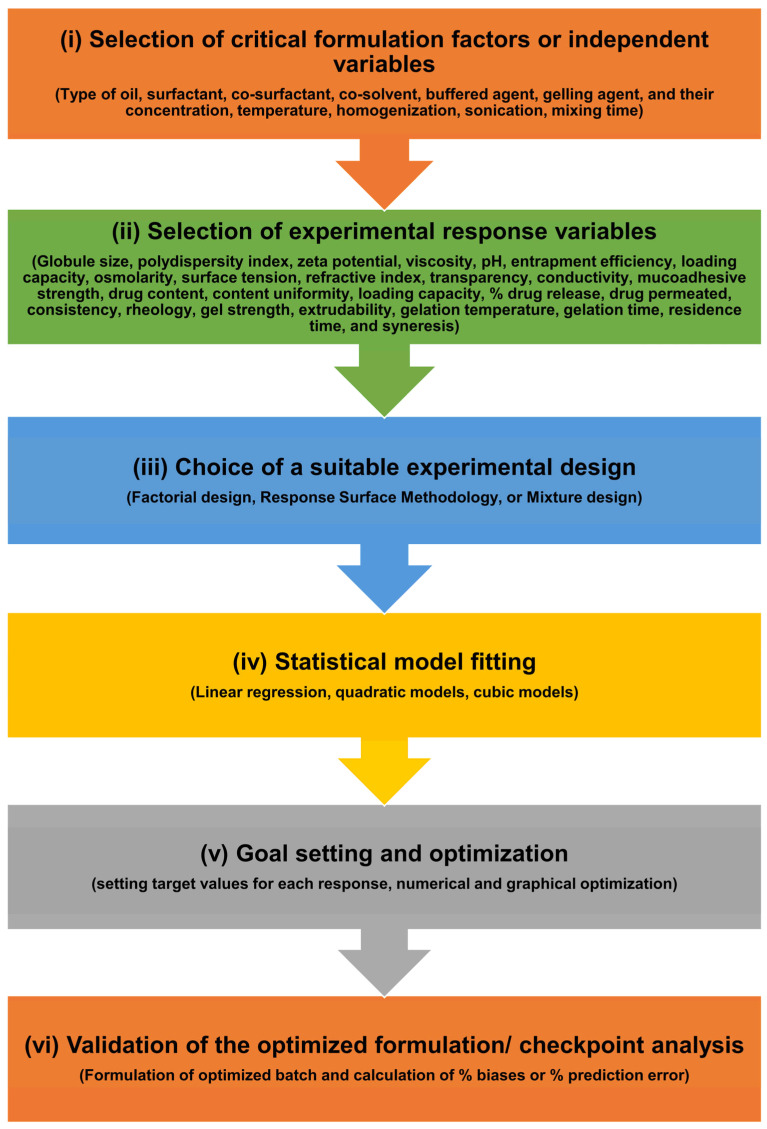
Key considerations for the optimization of ophthalmic emulsions and emulgels. This figure illustrates the key considerations for developing ophthalmic emulsions and emulgels, such as the (**i**) selection of critical formulation factors or independent variables, (**ii**) selection of experimental response variables, (**iii**) choice of a suitable experimental design, (**iv**) statistical model fitting, (**v**) goal setting and optimization, and (**vi**) validation of the optimized formulation/checkpoint analysis.

Design of Experiment (DoE) is a statistical technique widely used to study the use of various formulation variables for developing an optimized formula with the required pharmaceutical and therapeutic properties. DoE is widely exploited to develop and optimize ophthalmic emulsions and emulgels. The rationale for using DoE in the formulation development of emulsions and emulgels is to identify the critical factors that significantly influence the performance of the formulation, stability, and efficacy. The one-factor-at-a-time approach requires a huge amount of human effort, time, and expenditure to study the relationship between the factor and response. In contrast, quality by design (QbD)-based systematic optimization allows an improved understanding of the factor–response relationship with minimal resources. Mainly, in QbD, the desired critical quality attributes (CQAs) are defined, and the effect of each independent factor is studied. This allows the development of consistent batches with required attributes. The ophthalmic products need careful optimization to achieve high-quality pharmaceutical properties such as safety, efficacy, and improved patient compliance. Various statistical software packages (Design-Expert^®^, Minitab^®^) are used for the formulation optimization [15]. Key considerations for the optimization of ophthalmic emulsions and emulgels are shown in Figure 5.

### 10.1. Selection of Critical Formulation Factors

The initial step in the DoE is to identify major factors that affect the physicochemical, pharmaceutical, and biological properties of ophthalmic emulsion or emulgel. These factors include critical material attributes (CMAs) and critical processing parameters (CPPs). Various CMAs considered for the development of emulsions include the type of oil (natural, modified) and concentration of oil phase, type of surfactant and cosurfactant (anionic, cationic, non-ionic, or zwitterionic), type and concentration of cosolvent, and type (buffered solution, purified water) and volume of water. Various CPPs for ophthalmic emulsions include temperature, homogenization, sonication, mixing time, etc. Similarly, the CMAs for ophthalmic emulgels include all the CMAs for emulsions plus type and concentration of the gelling agent, pH, and ionic strength. The CPPs for emulgels include the swelling time, shear rate/mixing conditions, and temperature.

### 10.2. Selection of Response Variables

Various response variables include the globule size of the emulsion, polydispersity index, viscosity, pH, entrapment efficiency, loading capacity, zeta potential, osmolarity, surface tension, refractive index, transparency, conductivity, mucoadhesive strength, drug content, content uniformity, loading capacity, % cumulative drug release, and the cumulative amount of drug permeated. Various response variables for the emulgel include the pH, consistency, texture, homogeneity, spreadability, rheology, gel strength, extrudability, gelation temperature, gelation time, swelling ratio, gel residence time, syneresis, drug content, content uniformity, % drug release, and ocular permeability.

### 10.3. Choice of Experimental Design

The most commonly used designs of the experiments in formulation optimization of emulsions and emulgels include the following:

- Factorial design;
- Response surface methodology (RSM);
- Mixture design.

#### 10.3.1. Factorial Design

Fractional factorial and full factorial designs are mostly used to optimize emulsions. The full factorial design analyzes all probable combinations of the formulation factors. Such a design is useful for studying the interaction effects among various factors. The fractional factorial design is a simplified form of the full factorial design that analyzes a fraction of the total combinations/probabilities. This design is useful for studying a large number of factors while reducing the experimental workload. Several recent examples demonstrate the use of factorial design in the development and optimization of ophthalmic emulsions.

A 2^3^ full factorial design was used to formulate and optimize rifampicin-loaded cationic NEs using oleic acid, Poloxamer 188, Polysorbate 80, polymyxin B, and chitosan by high-pressure homogenization to treat ocular tuberculosis. The effects of cationic agents (chitosan chloride or polymyxin B sulfate), frequency of rotation, and incubation time were studied on globule size and zeta potential. The statistical analysis and formulation optimization were performed using Minitab^®^ software. The optimized NEs showed a nanometric globule size (150 nm), excellent zeta potential (51.3 mV), and mucoadhesive properties [107].

Cationic MEs of clotrimazole were formulated using oleic acid, Cremophor^®^ EL, Transcutol^®^ HP, and chitosan by the spontaneous emulsification method with continuous stirring. The formulation was optimized using a 2^2^ × 3^1^ full factorial design by Design-Expert^®^ software. The percentage of surfactant-to-cosurfactant mixture (S_mix_), type of surfactant, and ratio of surfactant to cosurfactant were chosen as independent variables, and their effects on the globule size, zeta potential, and polydispersity index (PDI) were investigated. The levels of the independent factors were chosen based on pilot studies using a pseudoternary phase diagram. The optimized MEs demonstrated a lower globule size, PDI, and optimum zeta potential with a spherical morphology. The optimized formula showed no histological abnormalities in retinal tissues and showed improved antifungal activity over 12 h compared to the drug suspension. The chitosan-coated cationic MEs demonstrated prolonged ocular retention due to their mucoadhesive nature [30].

MEs of quercetin were prepared using oleic acid, Transcutol^®^ P, Span^®^ 20, Tween^®^ 80, and propylene glycol, and the formulation was optimized by a full factorial design. The effect of the surfactant/cosurfactant ratio and percentages of oil and water was studied on globule size, viscosity, release, pH, diffusivity, and flux. The optimized MEs showed significantly improved transcorneal permeation compared to aqueous suspension [110].

In another study, ophthalmic NEs of brinzolamide and brimonidine tartrate were formulated and optimized using a full factorial design using Minitab^®^ software. The concentrations of castor oil and S_mix_ (polysorbate 80 and glycerol) were considered as independent variables, and their effect on the average globule size and % transmittance was studied. The optimized NEs were found to be transparent with a nanometric globule size. They maintained preservative efficacy, non-irritating properties, and a sustained drug release profile compared to the marketed product, Simbrinza^®^. The ex vivo ocular permeation across goat cornea demonstrated improved drug permeation compared to the marketed product [97].

#### 10.3.2. Response Surface Methodology (RSM)

The RSM is an advanced design of experiments that allows for the simultaneous optimization of multiple variables/factors and their interactions. It includes using statistical models to predict optimal formulation variables by analyzing the response variables (e.g., drug release, globule size, viscosity, etc.). Central composite designs (CCDs), Box–Behnken designs (BBDs), and optimal designs are mainly used to analyze the relationships among factors and responses.

The CCD model was used to statistically analyze and optimize the ophthalmic MEs of naringenin for effective treatment of corneal neovascularization. The weights of oil and S_mix_ were considered as independent variables, and their effects on dependent variables (droplet size and % drug loading) were studied using Design-Expert^®^ software. The formulation was prepared by the spontaneous emulsion method, and optimized MEs showed an ultra-low nanometric size (13.22 ± 0.13 nm), excellent entrapment efficiency (99.63 ± 0.03%), drug loading (2.92%), and thermodynamic stability. It showed no cytotoxicity against HCECs and no clinical signs of ocular irritation. The hydration level (80.76 ± 0.57%) maintained the integrity of the cornea after MEs’ instillation without epithelial and endothelial cell damage. The MEs formulation demonstrated significantly higher drug permeation across the cornea compared to the drug suspension. The developed ophthalmic MEs showed significantly higher concentrations of the drug in tears, the cornea, conjunctiva, and aqueous humor compared to a drug suspension. The ocular pharmacokinetics showed a 2.15-fold, 1.45-fold, and 1.35-fold increase in the area under the curve (AUC_0–120_ min) over 120 min in the conjunctiva, cornea, and aqueous humor, respectively, with drug-loaded MEs compared to a drug suspension in rabbit eyes. The MEs (0.5% naringenin) showed comparable efficacy to that of dexamethasone (0.025%) in the inhibition of CNV in the CNV model [111].

MEs of osthole were prepared using Capryol-90, Cremophor^®^ EL, Transcutol^®^ P, and sodium hyaluronate using phase inversion emulsification and optimized by CCD using Design-Expert^®^ software. Various independent factors, such as the oil concentration and composition of surfactant to cosurfactant (k_m_ value), were considered, and their influence on the globule size and PDI was studied. The optimized MEs showed a very low globule size (16.18 ± 0.02 nm) and PDI (0.09 ± 0.00). It showed no in vitro cytotoxicity against HCECs and was found to be biocompatible with the rabbit eye without causing any irritation. The ocular pharmacokinetics on the rabbit’s eye revealed 28.2 and 102.34-fold higher pharmacokinetic profiles (AUC_0–t_) in the cornea and conjunctiva compared to the drug suspension. Further, the osthole-loaded MEs (0.1%) presented a similar efficacy to marketed dexamethasone eye drops (0.025%) on CNV in alkali burn-based (NaOH solution) mouse models [115].

NEs of luliconazole were prepared using ethoxylated hydrogenated castor oil, Capryol 90, and Transcutol^®^ P and optimized by CCD for fungal keratitis. The optimization was carried out with respect to the drug content and globule size, and the influence of oil and the surfactant–cosurfactant ratio was studied using Design-Expert^®^ software. The NEs showed a very low globule size (18.43 ± 0.05 nm), low PDI (0.070 ± 0.008), and high entrapment efficiency of 98.37 ± 0.47%. The NEs demonstrated increased drug release compared to a drug suspension and significantly increased the antifungal activity compared to a drug suspension. They showed good tolerance in rabbit eyes and did not cause any irritation. Further, the ocular pharmacokinetics showed improved bioavailability of NEs compared to a drug suspension [100].

NEs of isoliquiritigenin were prepared with dicaprylate, propylene glycol, Cremophor^®^ EL, polyethylene glycol 400, and sodium hyaluronate and optimized by CCD. The effects of oil and surfactant–cosurfactant on the entrapment efficiency and globule size were studied using the response surface methodology. The optimized formula showed a globule size of 34.56 ± 0.80 nm with a very low PDI (0.048 ± 0.022) and entrapment efficiency of 99.96 ± 0.02%. The drug release and permeation of optimized NEs were found to be significantly higher than their suspension. Further, the study demonstrated no cytotoxicity in HCECs and no irritation in the New Zealand white rabbit eye. The NEs showed bioavailabilities of 7.80-fold, 5.76-fold, and 2.13-fold higher than drug suspensions in the cornea, tear, and aqueous humor, respectively. The efficacy of NE treatment (0.2% isoliquiritigenin) was found to be comparable to that of dexamethasone treatment (0.025%) in the inhibition of alkali (NaOH solution) burn-based corneal neovascularization in BALB/c mice eyes [103].

Kassem et al. used the BBD model to optimize dorzolamide hydrochloride-loaded ophthalmic cationic NEs. The % surfactant, oil, and cationic agent were considered independent variables, while the droplet size, zeta potential, and PDI were used as dependent variables for investigating the factor–response interaction and optimizing the formulation. The optimized formulations showed a lower globule size (336.300 nm), optimum zeta potential (32.500 mV), and PDI (0.209) and demonstrated their potential for topical ocular use. The formulation was also found to be thermodynamically stable and non-irritant to the ocular surface, showed a sustained drug release, and significantly improved therapeutic activity compared to a drug solution and marketed eye drops for glaucoma [108].

#### 10.3.3. Mixture Design

Mixture design is a special type of experimental design that involves the blending of formulation components (proportion of oil, surfactant, water) in fixed proportions. This design is especially useful for the optimization of emulsions and emulgels, where the goal is to estimate the optimal proportions of formulation components to achieve a product with desired characteristics. In a mixture design, the percentage of each component is more crucial than its absolute quantity since the constituents must always sum to 100% *w*/*w*, whereas other statistical designs rely primarily on the actual quantities of ingredients to achieve the intended outcomes [138]. Simplex lattice design [15,81], simplex centroid, screening, and optimal designs are various types of mixture designs that are mostly used for formulation development and optimization of emulsions.

SNEDDSs of resveratrol and melatonin were prepared using Capryol^®^ PGMC, Tween^®^ 80, and Transcutol^®^ P. The simplex lattice design was used for ternary-phase diagrams, with the concentrations of oil, surfactant, and cosurfactant as independent variables and % transmittance as dependent variables. Further, I-Optimal design was used for formulation optimization using the concentration of oil, surfactant, cosurfactant, and type of surfactant as independent variables and the globule size, emulsification time, and % transmittance as dependent variables using Design-Expert^®^ software. The formulations showed a nanometric globule size (13.26 ± 0.07–127.29 ± 1.12 nm), emulsification time (12.04–15.76 s), % transmittance (88–100%) with excellent stability, and mucoadhesive property. They demonstrated excellent cytocompatibility with corneal epithelium cells and showed potential for ocular administration [81].

Dhaval et al. used a simplex lattice design for the optimization of MEs-based in situ gel for ophthalmic drug delivery of sparfloxacin [15]. The amount of oil/lipid phase (Labrafil M1944 CS), S_mix_ (Transcutol^®^ P and Acrysol™ 140), and water were chosen as the independent variables, and their influence on globule size and PDI were selected as dependent variables. The levels of factors were selected based on the microemulsion region of the pseudoternary phase diagram, and the formulation optimization was carried out using Minitab^®^ software [15]. Then, the optimized MEs were used for the formulation of in situ gelling MEs using Poloxamer 407 as a thermoresponsive gelling agent. The optimized formula showed excellent pharmaceutical properties and was found to be non-irritant. It showed the in situ gelling property at ocular temperature, sustained drug release for more than 10 h, and improved drug permeation across the goat cornea compared to the marketed eyedrop solution [15].

A mixture design (extreme vertices design) was used to develop and optimize itraconazole-loaded NEs using benzyl benzoate, Eumulgin CO40, and propylene glycol using a spontaneous emulsification method. The statistical analysis, factor–response relationship, and optimization were studied by Minitab^®^ software. The percentages of the oil phase, aqueous phase, and S_mix_ were considered independent variables, and their influence on globule size was studied. The optimized formulation showed a nanometric globule size with excellent thermodynamic stability, improved antifungal activity, and showed a seven-fold increase in the cumulative percentage of drug released compared to aqueous suspension [98].

MEs of brinzolamide were prepared using isopropyl myristate, Tween^®^ 80, and Transcutol^®^ P and optimized using a D-optimal mixture design. The globule size, zeta potential, viscosity, and transparency were considered as response variables, and the influence of the percentages of oil, surfactant, and water was studied by Design-Expert^®^ software. The optimized MEs showed a globule size of 41.69 nm, zeta potential of −9.496 mV, transparency of 1.483 NTU, viscosity of 170.8 cps, and pH of 7.646, which are found to be suitable for ophthalmic delivery. The MEs showed prolonged drug release and were found to be safe and non-irritant for ocular drug delivery [43].

### 10.4. Model Fitting

Various statistical models (e.g., linear regression, quadratic models, cubic models) are fitted to the experimental data to analyze the influence of each factor alone and in combination with other factors on the response/outcomes via model-coded equations and graphical data. The suitability of the experimental model is evaluated from a high model R^2^ value (0.999), non-significant lack-of-fit value (>0.05), low sequential *p*-value (<0.0001), reasonable difference among adjusted and predicted R^2^ values (<0.2), low standard deviation, and very high signal-to-noise ratio/adequate precision (>4.0).

### 10.5. Goal Setting and Optimization

Once the model is fitted and the effect of each factor on the responses is obtained, the optimization process begins by setting target values for each response (e.g., globule size reduction, decrease in the PDI value, increase in % loading capacity, increase in zeta potential). Then, with the use of an optimization tool in DoE software, the optimal level of formulation variables/factors to achieve desired formulation characteristics is established. The optimization is carried out using numerical and graphical optimization techniques.

### 10.6. Validation of the Optimized Formulation

After the software has predicted the optimized formula, the next step is to validate the predictability of the experimental model with actual experimental outcomes. This is carried out by preparing three to five optimized batches/checkpoint batches with the software-suggested optimized factors and testing them against the desired responses. The % biases or % prediction error is calculated as per the following equation to ensure the predictability of the optimized batch. A bias of <5% is accepted for such studies.

(1) $$\%\;prediction\;error=\frac{Experimental\;value-predicted\;value}{Predicted\;value}\times 100$$

## 11. Characterizations of Ophthalmic Emulsion and Emulgel Systems

### 11.1. Characterizations of Ophthalmic Emulsions (NEs, MEs, SEDDSs, SNEDDSs, SMEDDSs)

After the development of ophthalmic emulsions and emulgels, these formulations are mainly characterized by their physicochemical, pharmaceutical, and therapeutic properties. Key characterization parameters include pH, viscosity, osmolarity, surface tension, refractive index, transparency, globule size, polydispersity, zeta potential, ocular retention, drug–excipient compatibility, ocular compatibility, transcorneal or scleral permeability, safety, toxicity, irritancy, stability, and sterility. A summary of key characterization tests, acceptance criteria, and their relevance to the ophthalmic performance of ophthalmic emulsions and emulgels is shown in Table 2.

#### 11.1.1. Organoleptic Properties

The optimized formula is inspected for color, odor, transparency, precipitation, and phase separation [3,16,30]. The clarity of the emulsions is investigated against black and white backgrounds to observe light and dark particles [37].

#### 11.1.2. pH

The pH is mostly checked to evaluate the compatibility of the ophthalmic formulation with the ocular pH to avoid irritation. It is evaluated by a digital pH meter, litmus paper, or any other pH indicator [15,111,112]. The ideal pH for ophthalmic NEs or MEs should be close to that of tear fluid (pH: 7.4) to avoid irritation. Sometimes, buffers are included in the formulation to adjust the pH. Alkaline formulations (pH > 8.5) can cause major injuries to the cornea and internal structures of the eye. Low-pH emulsions are less risky than high-pH emulsions as they do not infiltrate the eyes [16]. The accepted pH range of formulations is within the range of 3.5–8.5 [17], or more precisely within 6.5–8.5 [16].

#### 11.1.3. Viscosity/Rheology

Viscosity is checked to investigate the applicability of the NEs/MEs/SEDDSs for topical administration. Very low viscosity causes spillage and provides low ocular retention, and very high viscosity causes difficulty in topical application [4]. Hence, an optimum viscosity is needed for ophthalmic preparations. It is evaluated by viscometers/rheometers without diluting samples [37,107,108]. The optimum viscosity of NEs allows prolonged ocular retention and maximizes the absorption. Gelling agents (carbopol, poloxamer) may be added to increase the viscosity and to obtain maximum ocular retention. The optimal viscosity of ophthalmic NEs that offer maximal corneal retention is within the range of 15–150 mPa·s [16].

#### 11.1.4. Osmolality

The osmolarity of ocular emulsions should be close to the osmolarity of tear fluid (270–310 mOsm/L) to avoid irritation [16]. It is measured by osmometers previously calibrated with glycerol [37,107,108]. In disease states such as DED, the homeostatic regulation of osmolarity fails, and a change in the osmolarity occurs. The ophthalmic emulsions should be designed as per the purpose and disease state.

The osmolarity is also checked from the morphology of red blood cells (RBCs). The lacrimal fluid and blood possess similar osmolarity. RBCs maintain their morphology in lacrimal fluid and blood. So, topical ocular formulations with similar osmolarity to blood and tears should exhibit similar behavior towards RBC. An equal volume of ophthalmic emulsion, isotonic 0.9% *w*/*v* NaCl solution, hypertonic 1.5% *w*/*v* NaCl, and hypotonic 0.45% *w*/*v* NaCl is added to RBCs and individually incubated. Then, the samples are dropped on a glass slide and observed for morphological alterations [117].

#### 11.1.5. Surface Tension

Surface tension is evaluated using a thermostatically controlled tensiometer [37,105,108,110]. The droplet’s volume is directly correlated with the solution’s surface tension. Since the volume of droplet/eyedrop is related to the quantity of contained drug, it is necessary to measure the surface tension of the product. Commercial eye drops deliver 25.1 to 56.4 µL with an average droplet volume of 39.0 µL. The inclusion of surfactants reduces the interfacial tension and the droplet size. A very high surface tension reflects lower tear film stability, whereas a low surface tension offers effective mixing with tear fluid, which leads to proper spreading of emulsion on the cornea and prolonged ocular retention. However, very low surface tension (<35 mN/m) might be painful and discomforting for the eye. A surface tension within 40–50 mN/m is reported as optimum for ocular delivery [3,4,17].

#### 11.1.6. Refractive Index (RI)

The RI value indicates the transparency and homogeneity of the formulation. It is evaluated using an Abbe refractometer [34,37,108]. An RI value close to water (i.e., 1.333) represents the transparency of ocular emulsions. A transparent formulation should allow clear vision without any blurring after application to the eye. The RI values from different regions of the formulation demonstrate the homogeneity of the product. A very similar RI value among different regions of same formulation indicates that the product is isotropic in nature. The RI of tears is within 1.340 to 1.360. A very close RI value of emulsions to that of tear fluid or a value less than 1.476 is desired for ophthalmic use without causing any blur effect or patient vision discomfort [3,17].

#### 11.1.7. Impurity Analysis

Impurities are generated from the ophthalmic emulsions during storage and exposure to various stability conditions. High-performance liquid chromatography (HPLC) or liquid chromatography–mass spectrometry (LC-MS) methods are used to evaluate the degradation products in ophthalmic NEs and active pharmaceutical ingredient (API). Impurity profile analysis is important during different stages of the shelf life to assure the safety of the product [16].

#### 11.1.8. Polarized Light Microscopy

Polarized light microscopy is used to verify the isotropic nature of emulsions. Briefly, a drop of the sample is observed under a cross-polarized light. The crystalline material possesses optical birefringence, whereas the amorphous substance does not possess any birefringence since the anisotropic liquid crystals mainly interfere with a polarized light, but the isotropic materials do not. The absence of bright light birefringence or the appearance of a black field is the outcome of the isotropic nature of emulsion [110].

#### 11.1.9. Percentage Transmittance (%T)

The %T represents the transparency of the emulsion. The higher the %T, the better the vision, and the lower the blurring effects. A transparent product is an indication of the effective formulation of NEs or MEs. The nanometric globule size of the NEs and MEs allows the light to be transmitted; hence, such formulations appear transparent or translucent [3]. It is evaluated by UV-visible spectroscopy at 650 nm [34,101]. A %T of >98% reflects the transparency of the product and its suitability for topical ocular application [17].

#### 11.1.10. Cloud Point

Cloud point is the temperature at which surfactants undergo a phase transition, forming a cloudy/turbid product due to the phase separation/breaking of emulsion. It is observed visually due to turbidity. The changes in globule size and homogeneity corroborate the breaking of the emulsion. Briefly, the study is carried out by diluting (1:10–1:300, *v*/*v*) the ophthalmic emulsion with simulated tear fluid and subjecting it to a thermostat bath with a gradual increase in temperature. Then, the temperature at which the formulation becomes turbid is recorded. Further, the sample is analyzed by a turbidimetric UV method at 650 nm to observe the change in transparency [81].

#### 11.1.11. Conductivity

The conductivity of emulsions is measured by a digital conductivity meter [34,98]. This study explains the type of emulsion. Since the water is the external phase, the o/w type of emulsions shows a higher conductivity compared to the w/o type.

#### 11.1.12. Robustness to Dilution

Emulsions should not show any sign of cracking or separation upon the addition of a continuous phase/dispersion medium [37]. If the NE/ME is extensively diluted with water without precipitation, flocculation, or phase separation, it can be said to be o/w type. If the emulsion is exclusively diluted with oil, it is a w/o type. The robustness to dilution of the ophthalmic emulsions is also checked with double-distilled water, simulated tear fluid, and PBS (pH 7.4) with a dilution of 10–1000 times, observing the changes in precipitation or phase separations after 24 h [108]. The formulations without any sign of precipitation represent dilution stability and have a potential ocular use with dilution.

#### 11.1.13. Mucoadhesive Strength

The mucoadhesion property of NEs or MEs allows prolonged ocular retention. The mucoadhesive strength is evaluated by a texture analyzer and modified balance method [3]. The mucoadhesive interactions are studied by various spectroscopic methods, such as attenuated total reflectance–Fourier transform infrared spectroscopy (ATR-FTIR) and ^1^H and/or ^13^C nuclear magnetic resonance (NMR) [46]. The mechanistic interaction with mucus at the macromolecular level is studied by rheological analysis [46]. Mucus acts as a viscoelastic gel-like material, and upon contact with another macromolecule, such as a polymer, the rheological properties are altered, which is investigated by rheology study [46]. Another technique, atomic force microscopy (AFM), is used to study the conformation and mechanistic understanding of mucoadhesion [46]. The interactions among mucin and mucoadhesive formulations are also studied by in vitro cell culture studies [46].

The in vitro mucoadhesion is also tested by measuring the zeta potential. Briefly, the ophthalmic emulsion is incubated with different concentrations of mucin (0.025–0.5 mg/mL) and mixed for 30 min, followed by measurement of the zeta potential. The terminal oligosaccharide chains of the mucin contribute a negative surface charge due to the existence of sulfate and sialic acid groups. Upon incubation of cationic emulsions with mucin solution, the magnitude of the zeta potential is lowered, which represents successful mucoadhesion [107,108,116].

Another method involves the ocular formulation being incubated with mucin or artificial tear fluid, and the change in viscosity upon mixing is studied by a viscometer. By subtracting the viscosity of the formulation with artificial tear fluid from the viscosity of the formulation with mucin, the increase in viscosity due to bioadhesion is obtained. Then, the obtained data is translated to bioadhesion force by multiplying it by the shear rate [67].

Ex vivo mucoadhesion study can be carried out using the falling liquid film method. Briefly, the freshly excised cornea is collected, and the formulation is applied to the cornea surface, which is tied over a polyethylene support. Warmed PBS (pH 7.4) is peristaltically pumped over the mucosa for 10 min, and the drug in the collected perfusate is quantified spectrophotometrically. The quantity of adhered drug is quantified as the difference between the total quantity applied and the total quantity of drug that flowed. The mucoadhesion strength is calculated as the ratio of adhered drug quantity to the applied drug quantity [116].

#### 11.1.14. Ocular Retention

Prolonged ocular retention allows improved drug absorption and ocular bioavailability. In vivo ocular retention is evaluated by γ-scintigraphy [109], surface plasmon resonance spectroscopy, and fluorescence imaging [1]. The nanoglobules of these emulsions offer a highly effective surface area, better ocular retention, and improved drug absorption [3].

#### 11.1.15. In Vitro Gelation Study

The in situ gelling ability of an ophthalmic emulsion is tested by incubating with freshly prepared simulated tear fluid and incubating at the ocular temperature. The time required for the appearance of gelling consistency is evaluated visually [15]. In another way, the emulsion is placed in a glass vial, and the temperature is gradually increased from 15 °C to the ocular surface temperature (33–34 °C) at a heating rate of 1 °C/min using a water bath. The gelation temperature is noted by intermittently observing the consistency changes/gelling consistency by inverting the vial [93].

#### 11.1.16. Dye Test

In addition to conductivity and dilution studies, the dye test is used to study the type of emulsion. If the water-soluble dye is added to an o/w type of emulsion, it shows complete fluorescence/color due to the solubility in the external phase. An oil-soluble dye (e.g., Sudan III) is added to an o/w emulsion; it shows a dotted fluorescence/color pattern due to its solubility in the internal oil globules.

#### 11.1.17. Globule Size (Z_avg_), Polydispersity Index (PDI), and Zeta Potential (ZP)

These parameters are mainly evaluated by photon correlation spectroscopy (PCS) or the dynamic light scattering method (DLS) using a particle size analyzer [3,4,15]. Z_avg_ is an indication of the globule size of the NEs/MEs. The smaller globule size offers an increased effective surface area, improved solubility, and enhanced permeation of drugs across the ocular layers [16]. Smaller globules (<200 nm) can be transported via receptor-mediated endocytosis, whereas larger globules are internalized by phagocytosis [84]. The PDI indicates the homogeneity of dispersed globules, and it ranges from 0.0 to 1.0. A PDI value closer to zero (<0.3) indicates a highly homogenous nature of globules and narrow size distribution, whereas a value >0.3 indicates heterogeneity and a broad size distribution. A very low PDI value avoids Ostwald ripening and indicates stability of the emulsion. The ZP reveals the colloidal stability of emulsions. A higher ZP (>±30 mV) represents more repulsive force between globules, which avoids aggregation/coalescence and indicates prolonged stability. A value > ±20 mV provides short-term stability, whereas very low ZP (<±5 mV) represents a rapid aggregation potential of oil globules and low stability [84]. The non-ionic surfactants offer low ZP values but still ensure dispersion stability due to steric stabilization [138].

#### 11.1.18. Morphology

The morphology reveals features of the developed emulsion, size, and size distribution. It is evaluated by transmission electron microscopy (TEM) [30,111] or high-resolution transmission electron microscopy (HRTEM) [34,95], high-resolution scanning electron microscopy (HRSEM) [77], scanning probe microscopy (SPM), or atomic force microscopy (AFM) [3,4]. In addition to the morphological features, it reveals the elemental composition through the integrated energy-dispersive X-ray spectroscopy (EDX) system and crystallinity information by the selected area electron diffraction (SAED) attachments. A diffused SAED pattern represents an amorphous nature, whereas a dotted pattern indicates crystallinity.

#### 11.1.19. Drug Content and Content Uniformity

This is evaluated to estimate the total content of the drug per unit volume of the formulation, which is represented by the percentage. The evaluation of drug content at different locations in the same product and comparison is used for the evaluation of content uniformity. Analytical techniques (UV-vis spectroscopy) [15,97], HPLC [37,95], and LC-MS) are mainly used for the estimation of the drug content. Briefly, the emulsion is diluted with methanol or other organic solvent/extracting solvent, centrifuged, and diluted, and the drug content is analyzed by analytical techniques [84]. The value of drug content within 85–115% (±15%) is considered a good product with content uniformity, whereas the value of 75–125% (±25%) or more is not accepted as a good product as per USP chapter <905> [16]. The percentage drug content is evaluated using the following Equation (2) [108].

(2) $$Drug\;content\;\left(\%\right)=\frac{Estimated\;amount\;of\;drug}{Initial\;amount\;of\;drug\;added}\times 100$$

#### 11.1.20. Entrapment Efficiency

This is the ratio of the amount of entrapped drug in the formulation to the total amount of drug taken for formulation development. The difference between the total amount of drug and the free drug provides information about the amount of entrapped drug. The free drug is estimated by subjecting the emulsion to ultrafiltration and centrifugation, followed by collecting the filtrate containing the free drug. The % EE is calculated using Equation (3) [100,107,111,112,115].

(3) $$Entrapment\;efficiency\;\left(EE\right)=\frac{Amount\;of\;entrapped\;drug}{Total\;amount\;of\;drug\;added}\times 100$$

#### 11.1.21. Loading Capacity

This is the ratio of the entrapped drug to the total amount of formulation. The entrapped drug or loaded drug is estimated by UV-vis spectroscopy, HPLC, or LC-MS methods. Loading capacity is calculated using the following equation [111,112,115].

(4) $$Loading\;capacity\;\left(LC\right)=\frac{Amount\;of\;entrapped\;drug}{Total\;amount\;of\;drug\;and\;excipient}\times 100$$

#### 11.1.22. Drug–Excipient Compatibility

This is studied by Fourier transform infrared spectroscopy (FTIR) [115] or ATR-FTIR [34,37,112,117]. FTIR is a representative of functional groups and chemical integrity. A small change in the chemical structure is represented by major spectral changes. Any interactions between the selected excipients and the drug are represented by the disappearance or shifting of the characteristic vibration peaks of the drug. Additionally, the FTIR also reveals the effective loading of drugs into the internally dispersed phase. The vibrational spectra of drug-loaded emulsions, similar to blank emulsions, are an indication of effective loading into the globules. Thermal analysis techniques, such as differential scanning calorimetry (DSC) and thermo-gravimetric analysis (TGA), are also used to evaluate the compatibility among drugs and excipients by recording the thermogram of a drug, formulation excipients, physical mixture of drug and excipients, and optimized formulation [34,71,107,117].

#### 11.1.23. Stability Studies

Thermodynamic and kinetic studies are mainly performed to represent the stability of NEs or MEs/SEDDSs. The thermodynamic stability of such is evaluated by exposing the product to several thermodynamic stress conditions, such as the heating–cooling cycle (40 °C and 4 °C) and freeze–thaw cycle (−21 °C and 25 °C), each for 48 h, and centrifugation (6000–13,000 rpm for 15–30 min) [36,93,101,102,111,119]. The kinetic stability is evaluated by exposing the product to room temperature and evaluating the colloidal properties (Z_avg_, PDI, ZP), drug content, content uniformity, and loading capacity at various time points [3]. An accelerated stability study is carried out as per the International Conference on Harmonization (ICH Q1) guideline at 40 ± 2 °C/75% RH ± 5% RH [15,43] for 6 months and evaluated intermittently for the change in globule size, PDI, zeta potential, pH, clarity, viscosity, drug content, etc. [95,110]. Long-term storage stability study is carried out at room temperature or 25 ± 2 °C/60% RH ± 5% RH for 12 months [95] and refrigeration (4 ± 2 °C) [81]. Then, the globule size, PDI, zeta potential, and drug contents are studied intermittently [96,108].

#### 11.1.24. Preservative Efficacy

This study is carried out to estimate the efficacy of antimicrobials in an ophthalmic nano/microemulsion to keep it free from microbial contamination. The preservative efficacy is carried out for multidose nanoformulations containing preservatives, such as benzalkonium chloride, methylparaben, propylparaben, benzyl alcohol, etc. It is studied by antimicrobial effectiveness testing where the NEs/MEs are incubated with a wide range of microbes (*Pseudomonas aeruginosa* and *Staphylococcus aureus*) at 37 °C for 24 h and their antimicrobial activity is measured in terms of the zone of inhibition [16,97].

#### 11.1.25. In Vitro Drug Release Study and Release Kinetics

The drug release directly affects the ocular bioavailability. It is evaluated to investigate the release pattern/profile of the loaded drug from the emulsion systems. It is mainly carried out by a dialysis bag method with the use of certain molecular cutoffs or a USP-type dissolution apparatus and suitable release medium, such as PBS, simulated tear fluid (STF), simulated aqueous humor fluid, simulated vitreous humor fluid, etc. [117]. The sink condition is maintained by the addition of cosolvents or surfactants in the release medium. The temperature is set at 34 ± 0.5 °C by a controlled magnetic stirrer to match the ocular surface temperature, and rotation is applied (~20–100 rpm) to imitate the blinking of the eye [16]. The cumulative % of drug release is estimated using UV-visible spectroscopy, HPLC, and LC-MS techniques and plotted against time [30,35,93,101]. To understand the release kinetics, the release data are fitted to various kinetic models, such as zero order, first order, Korsmeyer–Peppas, Baker–Lonsdale model, Higuchi, and Hixson–Crowell cube root law. The release exponent (n) of the Korsmeyer–Peppas model is used to understand the release mechanism [35,36,98,108,110,115].

#### 11.1.26. Diffusion Study

This study is carried out using a vertical or horizontal Franz diffusion apparatus. The donor chamber is filled with formulation, and the receptor chamber is filled with diffusion media (PBS, STF, etc.), with rotation by magnetic stirring to imitate the blinking of the eye. A synthetic membrane (cellulose or dialysis) is sandwiched between the donor and receptor compartments, and the surface is maintained at 34 ± 0.5 °C to achieve the ocular surface temperature. At various time intervals, aliquots are withdrawn from the receptor chamber and analyzed by spectroscopic or chromatographic methods [16].

#### 11.1.27. Ex Vivo Transcorneal/Scleral Permeation Study

The ex vivo corneal/scleral permeability is studied using excised cornea/sclera of animals such as rabbits, pigs, goats, or cows [3,15]. The study is carried out under occluded or nonoccluded conditions using a vertical or horizontal Franz diffusion apparatus. Occasionally, the study is carried out under static and simulated tear flow conditions [71]. The eyeball is collected, dissected to collect the sclera or cornea, processed, and sandwiched between the donor and receptor compartments of the diffusion cell with the epithelium towards the donor side. The receptor compartment is filled with diffusion media, such as PBS (pH 7.4), STF (pH 7.4), simulated aqueous humor fluid, or simulated vitreous humor. The temperature is kept at 34 ± 0.5 °C to match the ocular surface temperature, and rotation is applied (~20–100 rpm) to imitate the blinking of the eye. Sodium azide (0.0025% *w*/*v*) is added to the diffusion medium to avoid the growth of microbes. A specified quantity of NEs or MEs is applied uniformly over the tissue. Aliquots (~0.1–2 mL) are collected from the receptor compartment at various time points, diluted suitably, and the drug quantified using validated analytical techniques. The cumulative amount of drug permeated through a unit area of ocular tissue at a predetermined time is calculated using HPLC, LC-MS, and UV-visible spectroscopy [35,97] and plotted against time. The permeability coefficient (kp), steady-state flux (J_ss_), maximum flux (J_max_), and enhancement ratio (Er) are calculated and compared with a drug solution or suspension [3,16,34,84,99,103,111].

#### 11.1.28. Depth of Permeation by Confocal Laser Scanning Microscopy (CLSM)

Albino rabbits or Sprague–Dawley rats are used for the CLSM study to evaluate the depth of penetration. Fluorescent dye (rhodamine B or coumarin-6)-loaded emulsions and fluorescent dye solutions are compared to evaluate the depth of penetration. After 24 h post-administration, the eyes of each group are enucleated, rinsed in PBS, and fixed in 10% (*w*/*v*) paraformaldehyde at 4 °C for 24 h. Sometimes, various time points are considered (0.5 h, 1 h, 2 h, 4 h, and 6 h), and the animals are grouped accordingly to evaluate the fluorescent-based pharmacokinetic study [118]. Then, after exposure to acetone and xylene, the paraffin blocks are prepared. Thin sections of tissue (5 µm) are prepared and further deparaffinized by exposing them to xylene. Then, the sections are visualized using CLSM at the respective excitation wavelength [34,117,118].

#### 11.1.29. Transepithelial Electrical Resistance (TEER) Value Determination

The ocular tight junctions develop electrical resistance. Hence, alteration in the resistance provides insights into the integrity of tight junctions [117]. For continuous TEER monitoring, a monolayer of cells is cultured on permeable supports (transwell inserts) and connected to an automated TEER system equipped with electrodes maintained at 37 °C. For background correction, a blank resistance is recorded with cell-free insert-containing media. Continuous TEER values per surface area of the monolayers (Ω·cm^2^) are recorded at defined intervals (5–15 min) under stable temperature and CO_2_ conditions. The TEER profile demonstrates real-time changes in barrier integrity, enabling the evaluation of cellular responses to drugs, and formulations [140,141].

#### 11.1.30. Ocular Biocompatibility, Toxicity, and Irritation Study

Ocular irritation and toxicity studies are carried out by in vitro, ex vivo, and in vivo methods.

##### In Vitro Methods

An in vitro cytotoxicity study is performed to study the toxicity and biocompatibility of the formulation with ocular cells. Ocular formulations should be tested for their biocompatibility, toxicity, and irritancy. Various cytotoxicity assays, such as the MTT assay, CCK-8 assay, and/or Alamar Blue assay, are performed using ocular cells (human corneal epithelial cells: HCECs, human retinal pigment epithelial cell line: ARPE-19, human retinal pigment epithelial cells: HRPEs, human corneal limbal epithelial cell line: HCLE, Statens Seruminstitut rabbit cornea: SIRC) to ensure the ocular biocompatibility of the developed formulations [4,67,77,111,112,115,117].

##### Ex Vivo Methods

The hen’s egg test–chorioallantoic membrane (HET-CAM) test is a study that examines the effect of samples on ocular vasculature. Since the CAM is similar to the vascularized tissues of humans, this test is usually preferred to investigate the irritancy of topical ocular formulations. For this experiment, fertilized hen’s eggs are collected, swabbed with 70% ethanol, and placed in an incubator at 37 °C, 67 ± 5% RH for 2 days. The eggs are rotated at an interval of 12 h for complete circumferential membrane formation. On day 3, after candling to identify decayed eggs, the eggs are broken at the tapering end, 1 mL of albumin is removed, and they are again incubated for another 2 days. On the 5th day after candling, 1 mL of formulation, negative control (0.9% NaCl, PBS pH 7.4), and positive control (0.1–1 N NaOH, acetone, propylene glycol) are added, and the photographs are captured at various time points to observe the change in vasculature [4,93,117]. Formulations inducing negligible vasculature alteration are considered nonirritant [117]. Clotting, hyperemia, and hemorrhage on CAM blood vessels are the signs of irritation [3].

A corneal hydration test is performed to study the irritancy effect of ophthalmic formulations. Cornea tissue is collected, weighed, and fixed between the donor and receptor compartments of the Franz diffusion cell. The receptor compartment is filled with suitable media (PBS pH 7.4), and the donor compartment is filled with test samples. The diffusion is allowed to continue for 1–2 h under temperature-controlled (32 ± 0.5 °C) magnetic stirring. Then, the cornea is removed and weighed, and the weight variation is evaluated. Significant variation in weight is an indication of possible edema by the applied formulation [102,117]. Other ex vivo studies, such as Bovine Corneal Opacity and Permeability (BCOP) tests and Isolated Chicken Eye (ICE), have been approved by the Organization for Economic Cooperation and Development (OECD) and Global Harmonized System (GHS) Category 1 for ocular irritation studies [3].

##### In Vivo Methods

In vivo ocular irritation of optimized NEs, MEs, or emulgels is assessed on New Zealand albino rabbits using the Draize test [3,34,95,115] following the OECD 405 guideline. NEs or MEs (100 µL) are applied to the lower cul-de-sac of the eye. In addition to formulations, saline (0.9% NaCl, PBS 7.4) is used as a negative control, and standard irritants, such as 1 N NaOH/1% sodium lauryl sulfate, are used as a positive control [93]. One of the eyes is treated with the aforementioned samples, and the remaining eye is an untreated control. The eyelids are gently closed for ~10 s to avoid the loss of instilled samples. Each animal is observed for any sign of ocular irritation, such as discharge, redness, conjunctival chemosis, iris, and corneal lesions at 5, 10, 15, and 30 min and 1, 2, 3, 6, 8, 12, and 24 h post instillation. The irritation score is provided as 0 (absence) to 4 (highest) grades. Then, the overall ocular irritation index is estimated by adding total scores over the studied time points. A score of 2 or 3 for any parameter or irritation index of more than 4 is considered an indicator of significant ocular irritation [16]. The ocular irritation is graded as (i) practically non-irritating (score 0–3), (ii) slightly irritating (score 4–8), (iii) moderately irritating (score 9–12), and (iv) severely irritating or corrosive (score 13–16). Slit lamp biomicroscopic evaluation, fluorescein staining, fluorescein angiography, and fundoscopy evaluations are carried out for a detailed investigation of anterior and posterior segments of the eye.

In vivo irritation studies have also been reported with mice using the Draize technique [120]. The samples of 15 µL are applied to one of the eyes, the other serving as an untreated control. The mice are observed for signs of ocular irritation using Draize criteria at various time points. Each group’s average ocular irritation score is calculated for the cornea, iris, and conjunctiva and interpreted for possible toxicity and irritation. The irritation score is graded from 0 to 16 (0–3: non-irritating, 4–8: slightly irritating, 9–12: moderately irritating; and 13–16: severely irritating or corrosive) [120]. In addition to that, the eyes are also stained with 2 μL of 0.5% fluorescein and analyzed using a fluorescence microscope for possible corneal lesions/abrasions [120]. Fundoscopy and fluorescein angiography are also used to obtain detailed insights into the posterior eye tissue.

A phenol red thread test is also carried out to observe potential ocular irritancy by emulsions. In this study, phenol red-impregnated cotton thread is inserted into the inferior fornix of the conjunctival sac. The thread changes its color from yellow to red upon wetting by tears due to its pH-sensitive nature. A length of wet red thread less than 10 mm within 15 s represents the deficiency of tears. The baseline is established by inserting the thread into the other eye and noting the color length after 15 s of insertion [42].

Histopathology studies are carried out to investigate the alteration in the histology of ocular tissues after the application of samples. These observations are carried out via ex vivo experiments or in vivo experiments. In ex vivo studies, the cornea is sandwiched between the donor and receptor compartment of a Franz diffusion cell, and the samples are applied. After a certain time period, the tissue is used for histopathology evaluation. In in vivo experiments, the eye is enucleated at the end of the irritation study, fixed in paraformaldehyde, and processed for histopathology study after staining with hematoxylin and eosin [34,95,116].

In the spectroscopy method, ATR-FTIR analyses are mainly carried out to investigate the interaction of NEs/MEs with corneal tissue. The samples are applied topically, and at the end of the study, the eye is enucleated, and the cornea is immediately separated gently. Then, the corneal tissues from each experimental group are subjected to ATR-FTIR spectroscopy. ATR-FTIR spectra are recorded within a frequency range from 600 to 4000 cm^−1^ at a resolution of 4 cm^−1^. Since the cornea mainly consists of collagen and water, a change in absorption intensity and shift in frequency of IR bands suggest the denaturation of proteins [96]. A DSC study can also be carried out to investigate the interaction of emulsions with ocular tissue. The DSC of the cornea and cornea treated with optimized ophthalmic emulsion is carried out, and the thermograms are compared to identify possible interactions. Shifting of the thermogram/phase transition event of the cornea is the indication of possible interaction of the ophthalmic formulation [110].

Schirmer’s test is a relatively quick and simple diagnostic tool for evaluating tear production by lacrimal glands and diagnosing ocular irritation or dry eye syndrome. Ocular irritation occurs due to decreased tear breakup time, representing the presence of tear film instability. This is caused by decreased tear production [142]. In this experiment, a small strip of filter paper (~5 mm by 30 mm) is placed in the lower conjunctival sac of each eye, and the eye is allowed to close in a quiet and controlled environment to avoid external factors from affecting tear production. Following the principle of capillary action, the watery content of the tear travels along the length of the paper strip. The rate of travel along the test strip is directly proportional to the rate of tear production. The test can be carried out with or without anesthesia [143]. After a certain time (5 min), the degree of wetting on the filter paper is measured in millimeters. Under normal conditions, the amount of wetting is usually >15 mm after 5 min, whereas a wetting of <5 mm over 5 min typically represents tear deficiency with significant ocular irritation or DED [143].

#### 11.1.31. Cellular Uptake Study

The cellular uptake is carried out by confocal laser scanning microscopy (CLSM). The ocular cells (RPE, HCEC) are seeded in a six-well plate and treated with fluorescent dye (coumarin-6)-loaded ocular emulsions [77,106]. Then, the cells are washed with PBS, fixed with 4% *v*/*v* formalin, followed by counterstaining with 4′,6-diamidino-2-phenylindole (DAPI) or Hoechst dye. The photomicrographs are captured using CLSM at various time points and analyzed by ImageJ for quantitative uptake analysis [77].

#### 11.1.32. In Vivo Uptake Study

The study is carried out using albino rabbits. The animals are housed individually in a standard facility. They are topically treated with coumarin-6-loaded emulsions for up to 7–14 days as per the study protocol. Then, the eyes are enucleated, and cryosections are prepared. The sections are visualized under fluorescent or CLSM to study the fluorescence at different regions of the eye, which predicts the depth of penetration [77].

#### 11.1.33. Ocular Pharmacokinetics Study

The ocular pharmacokinetic study is mostly carried out on rabbits. Other animals including rats are also reported for studying the ocular pharmacokinetics of emulsions. The animals are housed individually while being provided a standard diet and water. The ophthalmic formulation and control solution or suspension is administered topically into the lower eye cul-de-sac to various groups. At predetermined time points, tear fluid, aqueous humor, corneal, and conjunctival tissues are collected [111]. Fresh aqueous humor is collected from the rabbit eye by inserting a small needle across the cornea, above the corneoscleral limbus of the eye. Suitable extraction techniques are used to extract the drug from the collected tissue for its quantification [103,115]. The calibration curve is prepared by spiking a known concentration of drugs with the aqueous humor and quantifying by analytical techniques, such as HPLC [95,111], and LC-MS [100]. The bioanalytical method should be validated as per FDA guidelines [111]. Then, the amount of drug in the real sample will be evaluated by collecting the aqueous humor at various time intervals of the topical administered group, extracting the drug, and quantifying it using a validated analytical technique. Various pharmacokinetic parameters, including the maximum drug concentration (C_max_), time needed to reach the maximum drug concentration (T_max_) and the area under the curve (AUC_0–∞_), half-life (t_1/2_), and elimination rate constant (k_el_), are evaluated to analyze the pharmacokinetic profile [95,103,109,111,112,117]. Mice and rats can be used for pharmacokinetic studies. However, due to a limited volume of aqueous humor and tear fluid in mice and rats, eyes must be enucleated at various time points and quantified for the drugs in various ocular tissues by sophisticated analytical instruments with the use of validated bioanalytical methods. Rats have been used for ocular pharmacokinetics study by replacing drugs with fluorescent markers (coumarin 6) in ophthalmic emulsions. Plain coumarin 6 solution is used for comparison purposes. Such studies are carried out in dark conditions to avoid fluorescent quenching. After topical application of coumarin 6-loaded ophthalmic emulsions, the rats are sacrificed at a predetermined time period (0–6 h) and their eyes are enucleated, processed, and observed by a confocal microscope. The integrated fluorescence density is measured for different ocular tissues and compared [117].

#### 11.1.34. Sterility Testing

The ocular formulations must be sterile. Sterility tests confirm the absence of microbial contamination. The test must be conducted under aseptic conditions to avoid accidental microbial contamination during testing. The culture media required for the growth of aerobic and anaerobic bacteria and fungi are prepared as per the pharmacopeia. The sterility is evaluated by (i) the membrane filtration method (Method A) and (ii) the direct inoculation method (Method B). In the direct inoculation method, the sample is directly inoculated in tubes containing fluid thioglycolate medium or soybean casein digest medium and incubated in an incubator [15,43]. The membrane filtration method involves filtration of the ocular formulation through membrane filters (0.45 µm pore, 47 mm diameter) under vacuum. Then, the membrane is cut into two equal halves and kept in test tubes containing fluid thioglycolate medium or soybean casein digest medium and incubated for a definite period (14 days) [16]. The appearance of turbidity in the test tubes represents possible microbial contamination and a failure of product sterility. The direct inoculation method can also be used to check sterility. The formulation is directly inoculated using a sterile syringe into a fluid thioglycolate medium and incubated for 14 days at 30–35 °C to observe any microbial growth [15]. Alternatively, the ophthalmic emulsion (test), sterile saline solution (negative control), and bacterial culture (positive control) are incubated in Luria broth agar in Petri dishes. The plates are sealed with paraffin film and incubated at 37 °C in an incubator. The absence of the growth of microorganisms is an indication of sterility [117].

#### 11.1.35. Post-Sterilization Characterization

The emulsions are characterized after sterilization by filtration (0.22 µm) to evaluate possible alterations in the pharmaceutical properties, such as globule size, zeta potential, PDI, drug content, etc. [95,105].

#### 11.1.36. Pyrogen Test

Pyrogens are metabolic byproducts of microorganisms. Gram-negative bacteria mainly generate the most potent pyrogens. Such pyrogens, when introduced into the body, cause fever and a cascade of pathogenic responses (chills, body aches, cutaneous vasoconstriction, increased blood pressure). Endotoxins are a subset of pyrogens. They are water-soluble, insoluble in organic solvents, pass through 0.2 µm filters, and are not destroyed by autoclaving. The pyrogens can be tested in sterile preparations using a rabbit test and Limulus Amebocyte Lysate (LAL) test/bacterial endotoxin test. The rabbit test involves measuring the rise in body temperature after 3 h of i.v. administration of test samples. In the LAL test, the gelling property of lysates of *Limulus polyphemus* (horseshoe crab) is evaluated. It involves the incubation of a test sample with LAL reagent for 1 h at 37 °C and observation for the presence of gel. A sample containing endotoxins forms a firm gel. This method is simple, offers improved sensitivity, and is less expensive compared to rabbit tests [16]. Alternatively, a fluorometric method is a rapid and highly sensitive alternative method to animal-based pyrogen tests that can be used for the detection of pyrogens even at low concentrations. This measures changes in the fluorescence intensity of Rhodamine 6Zh in the presence of bacterial lipopolysaccharides. Free pyrogens enhance fluorescence, while intact microbial biomass initially quenches it [144].

### 11.2. Characterization of Ophthalmic Emulgels

#### 11.2.1. Organoleptic Properties

The color, odor, texture, and appearance of the ophthalmic emulgel are observed visually and compared with the marketed gel [122,131,145].

#### 11.2.2. Consistency and Homogeneity

The consistency and homogeneity of emulgels can be observed visually or evaluated by placing a small amount of gel between the thumb and the index finger and slightly rubbing to observe the presence of any masses or lumps.

#### 11.2.3. Spreadability

This assesses the spreading ability of the emulgel during topical ophthalmic application. An easily spreadable ophthalmic emulgel can be easily applied at low pressure, thus offering better patient compliance. A certain amount of weight is applied and maintained onto a gel to assess the increase in diameter due to spreading, and it is compared with the marketed gel. Alternatively, the spreadability of emulgel is measured using the ‘Slip’ and ‘Drag’ method. In this method, the emulgel is sandwiched between two glass slides with similar dimensions, and a specific weight is placed in a pan attached to the pulley with the help of a hook. The spreadability or spreading coefficient is evaluated by measuring the time it takes for a weighted upper plate to slide a particular distance over a gel layer sandwiched between two plates [131].

#### 11.2.4. Extrudability

Extrudability is expressed in terms of weights needed to extrude the emulgel from a collapsible tube. Easily extrudable emulgel is applied easily on the eye with limited pressure and offers improved patient compliance.

#### 11.2.5. pH

The pH of emulgel is evaluated to assess its compatibility with ocular pH to avoid irritation. An aqueous dispersion of the emulgel is made, and pH is evaluated using a calibrated digital pH meter [122,145]. The pH value for emulgel is compared with the marked gel.

#### 11.2.6. Osmolality

The osmolarity of ocular emulgels is measured by freezing point depression using suitable osmometers. Emulgels with optimum osmolarity avoid ocular irritation [145].

#### 11.2.7. Rheological Studies and Gelation Temperature

The rheology of emulgel is evaluated at a constant shear rate, increasing shear rate, and amplitude sweep mode using a rotational rheometer (cup-bob, cone-plate, or parallel plate). The rheogram of the emulgel is studied to evaluate its rheology and compared with the marketed gel. The gelation temperature (T_gel_) or sol–gel transition temperature of an in situ-forming ocular gel is evaluated by a rheometer. The study is conducted under thermal equilibrium. Initially, the rheology is assessed to find the linear viscoelastic region, followed by minor strain oscillatory shear applied at a constant heating rate to identify the T_gel_ of the ocular formulation. The gelation temperature of the in situ gelling emulsion is also tested by placing it in a glass vial and gradually increasing the temperature from 15 °C to the ocular surface temperature (33–34 °C) at a heating rate of 1 °C/min using a water bath. The gelation temperature is noted by observing the consistency changes/gelling consistency intermittently by inverting the vial [93,122,131,145].

#### 11.2.8. Drug Content and Content Uniformity

The drug content is the quantity of the API in the ophthalmic formulation. The drug content of the emulgel is determined by a validated HPLC/LC-MS or UV-visible spectroscopy method [122,131]. Briefly, a known quantity of emulgel is extracted with organic solvents (methanol/ethanol), vortexed, bath sonicated, centrifuged, and filtered through a syringe filter, and the drug content is estimated by validated analytical methods. In general, the drug content of 95% to 105% is accepted for ophthalmic formulations [16]. The content uniformity of an emulgel is estimated by quantifying the drug content in samples taken from different regions of the container and comparing the variations.

#### 11.2.9. Syneresis

Syneresis is an undesired natural phenomenon of emulgels in which unbound excess water is oozed out from the gel matrix due to shrinkage. It can be controlled by selecting an appropriate gelling agent at an appropriate concentration. It is measured using the centrifugation method by evaluating the initial and final weights of the emulgel after centrifugation, followed by decanting excess water. The syneresis of an emulgel is expressed as a percentage [146]. Lower % syneresis represents higher gel stability.

#### 11.2.10. Swelling Index

The swelling index of an ophthalmic gel affects its ability to hydrate, adhere to the ocular surface, and release the contained drug. Emulgels with an optimum swelling index of 20–30% or higher ensure a prolonged contact time with the eye, leading to improved ocular bioavailability and therapeutic efficacy. The swelling index also affects the mechanical properties of an emulgel and its ability to adapt to the dynamic ocular environment. It is estimated by dipping a known weight of emulgel placed in a porous aluminum foil, followed by dipping in an aqueous medium for a certain time, and reweighing. Then, the equilibrium percentage swelling is calculated from the weight of the swollen gel and the initial weight of the gel [131].

#### 11.2.11. Compatibility Study

The drug–excipient compatibility of the emulgel is investigated by ATR-FTIR spectroscopy by comparing the vibrational spectra of individual formulation components, physical mixture, and formulations [123]. Any interactions between the chosen excipients and the drug can be identified by the alteration or disappearance of the characteristic vibrational peaks of drugs.

#### 11.2.12. In Vitro Drug Release and Release Kinetics

In vitro drug release kinetics are analyzed through the dialysis bag method using cellulose dialysis tubing (MWCO 8000–14,000 Daltons). The emulgel is filled in a pre-activated dialysis bag and dipped in release media similar to drug release studies for NEs or MEs. A control gel loaded with a drug is used for comparison. The release kinetics are evaluated by fitting the release data to various kinetic models [122,131,145].

#### 11.2.13. Ex Vivo Transcorneal/Scleral Permeation Study of Emulgels

The ex vivo corneal/scleral permeability for emulgels is carried out similarly to that of NEs or MEs. In this case, only the emulgel is applied uniformly on the ocular surface, and samples are withdrawn at predetermined time intervals to estimate the drug content. The cumulative amount of drug permeated through a unit area of the ocular surface is calculated. The permeability coefficient, steady-state flux, and enhancement ratio are calculated and compared with a drug-loaded control gel [4,123].

#### 11.2.14. Stability Studies

The stability study of emulgel is carried out as per ICH guidelines (ICH Q1 R2). The emulgel is filled in the sterile collapsible tubes and subjected to long-term stability conditions (5 ± 3 °C) for 12 months or accelerated stability conditions (25 ± 2 °C/60 ± 5% RH) for 6 months. Periodically, at 0, 6, 9, and 12 months for long-term stability study and 0, 3, and 6 months for accelerated condition, the emulgels are evaluated for organoleptic properties, pH, homogeneity, consistency, spreadability, extrudability, viscosity, drug content, content uniformity, syneresis, and drug–excipient compatibility.

#### 11.2.15. Ocular Irritation and Toxicity Study

Ocular irritation and toxicity studies are carried out by in vitro, ex vivo, in vivo, and spectroscopic methods similar to those described under the characterizations of emulsions [145].

#### 11.2.16. Ocular Pharmacokinetics Study

The ocular pharmacokinetic studies of ophthalmic emulgel are carried out similarly to those for ophthalmic emulsions. The emulgels offer maximum ocular retention and excellent pharmacokinetic profiles compared to ophthalmic emulsions. Pharmacokinetic analysis is accomplished using pharmacokinetic software [145].

#### 11.2.17. Therapeutic Efficacy Study

After characterization of the ocular formulation and irritation studies, they are evaluated for therapeutic activity using various models. Their therapeutic activity is compared with a drug solution, suspension, drug-loaded control gel, or marketed formulations.

## 12. Sterilization of Ophthalmic Emulsions and Emulgels

Ophthalmic emulsion or emulgels should be sterile. Sterilization is a critical process in the formulation of ophthalmic emulsions and emulgels to ensure the safety and efficacy of the product for eye applications. These formulations are susceptible to microbial contamination, and since the eye is a sensitive organ, it is crucial to maintain the sterility of the product throughout its shelf life and use. The sterility of the ophthalmic formulations is mandatory to avoid microbial infections. An effective sterilization method ensures the absence of microorganisms.

### 12.1. Sterilization Methods

Several sterilization techniques can be employed for ophthalmic emulsions and emulgels, each with specific advantages and limitations:

#### 12.1.1. Moist Heat Sterilization (Autoclaving)

This includes exposing the formulation to high temperatures (typically 121 °C, 15 psi for 15–20 min) to kill microorganisms. For example, an in situ-forming gel of sparfloxacin MEs was reported to be sterilized by autoclaving for 20 min at 121 °C and 15 psi [15,84,99,120]. This method is suitable for heat-stable emulsions but may alter the physicochemical properties (e.g., viscosity, droplet size) or stability (lipid hydrolysis) of the formulation, especially for sensitive drugs [3]. This method is not used for emulsions containing heat-sensitive components, such as certain drugs or preservatives.

#### 12.1.2. Sterilization by Filtration

This involves passing the formulation through a sterile filter with a pore size of 0.22 microns to remove microorganisms [4,35,84]. This is mainly used for ophthalmic emulsions and emulgels as it preserves the stability and integrity of the formulation, especially for thermolabile drugs. This method is effective for pre-sterilized emulsions and emulgels but may not be suitable for emulsions with high viscosity or those containing certain large particles. The filtration method may affect the globule size, size distribution, and drug content. The globule size of more than 220 nm may clog the filter [3].

#### 12.1.3. Sterilization by Radiation

In one study, ophthalmic dexamethasone sodium phosphate-loaded w/o MEs were sterilized using Cobalt-60 gamma radiation. According to the European Pharmacopoeia, a dose of 25 kGy is considered the standard for achieving the required sterility assurance level. In addition to this standard dose, lower doses of 5 kGy and 15 kGy were also tested to evaluate their effect on microemulsion stability. The sterility test confirmed the achievement of sterility not only immediately after treatment but also after six months of sealed storage, as well as under in-use conditions (samples opened twice daily for 20 s over one week). Further, gamma radiation was reported to be an effective sterilization method for the ophthalmic MEs without significantly compromising its physicochemical stability [34].

In another study, in situ ocular NEs-based gels of terbinafine hydrochloride were reported to be sterilized by gamma radiation using a Cobalt-60 irradiator. To prevent potential side effects from the temperature increase associated with γ-irradiation, the samples were sterilized in the presence of dry ice. The drug release profile was evaluated before and after sterilization, to check the formulation performance, which was found to be maintained [147].

#### 12.1.4. Chemical Sterilization

This uses chemical agents like ethylene oxide to sterilize the formulation. This is a good option for formulations containing sensitive drugs or ingredients that cannot withstand heat or radiation. However, residual chemicals can sometimes remain in the product, necessitating proper purging and safety testing.

### 12.2. Sterilization Challenges

- Viscosity and stability: The gel phase of emulgels can make it more challenging to sterilize without affecting the viscosity and drug release properties. Thermal methods like autoclaving can degrade gel consistency, while filtration can be difficult for highly viscous formulations.
- Microbial growth post-sterilization: While sterilization kills microbes, post-sterilization contamination can occur if the packaging is not handled properly. Thus, maintaining a sterile manufacturing environment and using sterile packaging are crucial to ensuring product sterility.
- Preservative usage: To prevent microbial contamination during use, ophthalmic emulsions and emulgels often require preservatives (e.g., benzalkonium chloride, phenylmercuric nitrate or acetate, chlorhexidine, chlorobutanol, methylparaben, and propylparaben). However, preservatives are reported to induce conjunctival toxicity, tear film disruption, allergic symptoms, and corneal damage. The symptoms of preservative toxicity include foreign body sensation, discomfort on administration, irritation, burning, dry eye, tearing, and itching. They disrupt the glycocalyx and lead to ocular surface inflammation, allergy, fibrosis, and DED. Thus, the newer, less-toxic preservatives (polyquaternium-1, sorbic acid, polixetonium, sodium perborate) may be considered for reducing ocular toxicity [136].

## 13. Recent Patents on Ophthalmic Emulsions and Emulgels

Recent ophthalmic formulation patents (Table 3) demonstrate a strong focus on NEs, MEs, and SEDDSs for improving ocular drug delivery. Most inventions target DED using cyclosporine-based oil-in-water NEs, often combined with permeability enhancers or mucoadhesive polymers to improve stability, bioavailability, and patient tolerability. Several patents extend applications beyond dry eye, including ocular inflammation, DED, cataracts, presbyopia, myopia, and anterior segment diseases. Novel approaches include in situ gelling NEs, liposome/microemulsion-laden contact lenses, and self-nanoemulsifying systems, all aiming at sustained release, enhanced solubility of lipophilic drugs, reduced irritation, and prolonged corneal retention. Collectively, these patents reflect the global trend toward next-generation ophthalmic emulsions designed to overcome ocular barriers and optimize therapeutic outcomes.

## 14. Clinical Trials on Ophthalmic Emulsions and Emulgels

Recently completed clinical trials on ophthalmic emulsions and emulgels are listed in Table 4. Several clinical trials have evaluated ophthalmic emulsions, NEs, and MEs for ocular diseases, with multiple studies reporting favorable results. Phase 3 trials of brimonidine tartrate NEs (NCT03785340) for dry eye disease and clobetasol propionate NEs (NCT04246801, NCT04249076) for post-cataract surgery inflammation and pain demonstrated positive efficacy and safety profiles, supporting their therapeutic potential. Similarly, cyclosporine MEs (NCT04426240) showed beneficial effects in preventing post-cataract surgery dry eye syndrome, while several cyclosporine emulsions (e.g., NCT01109056 for pterygium, NCT04812951 for prophylactic cataract treatment, and NCT02139033 evaluating Retaine™ in dry eye syndrome) confirmed improvements in ocular outcomes. Furthermore, Phase 3 studies with difluprednate emulsions (NCT00406887, NCT00407056, NCT03693989) achieved positive results in managing uveitis and postoperative inflammation, reinforcing the clinical utility of emulsion-based ophthalmic formulations. Collectively, these trials highlight those ophthalmic emulsions, NEs, and MEs not only provide enhanced delivery of lipophilic drugs but also translate into clinically meaningful benefits across diverse ocular conditions.

## 15. Marketed Ophthalmic Emulsions

Marketed products of ophthalmic emulsions are listed in Table 5. Although still few in number, the marketed products reflect the translational potential of emulsion-based systems for ophthalmic applications.

## 16. Challenges in the Formulation and Development of Ophthalmic Emulsions

Despite extensive work on novel ophthalmic drug delivery systems, only a few emulsions have successfully reached the market. This limited clinical translation is primarily attributed to challenges such as biocompatibility and toxicity concerns, patient tolerability issues, preservative-related complications, selection of appropriate animal models, sterilization difficulties, stability and sterility maintenance, manufacturing scale-up and reproducibility, regulatory constraints, and cost-effectiveness.

### 16.1. Biocompatibility and Toxicity

Ophthalmic emulsions and emulgels must be biocompatible and non-irritating to ocular tissues. One of the key challenges in developing ophthalmic emulsions is ensuring the safety and biocompatibility of the oil phase. While oils play a crucial role in solubilizing lipophilic drugs and enhancing stability, certain vegetable oils have been associated with corneal and conjunctival toxicity. Additionally, rancidity or oxidation of the oil phase can generate harmful free radicals, potentially leading to ocular tissue damage [18].

Another significant challenge in ophthalmic emulsion formulation is selecting surfactants and cosurfactants that are both effective and biocompatible, as many can cause ocular irritation or toxicity [137,171]. Surfactants are reported to disturb the tear film and penetrate epithelial cell membranes, leading to potential damage of ocular tissues [172]. Achieving the right balance between stability and safety often requires minimizing the surfactant concentration without compromising emulsion quality [18]. Further, surfactants can be replaced with other stabilizers (polymers: cyclodextrin) to develop Pickering emulsions to reduce surfactant-related irritation and improve ocular biocompatibility [171].

### 16.2. Patient Tolerability

Patient tolerability remains a major challenge for emulsion-based ophthalmic formulations. Issues such as toxicity, ocular irritation, and blurred vision significantly affect patient tolerability. Achieving non-toxic, non-irritant, and transparent ophthalmic emulsions is essential to ensure patient comfort. Additionally, emulsions should be optimized to provide sustained drug release, thereby reducing the dosing frequency and improving patient compliance [171].

### 16.3. Preservative Concerns

Some of the conventional preservatives (e.g., benzalkonium chloride, methylparaben, and propylparaben) used in ophthalmic emulsions are reported to induce conjunctival toxicity, tear film disruption, allergic symptoms, and corneal damage. They disrupt the glycocalyx and lead to ocular surface inflammation, allergy, fibrosis, and DED. Thus, the newer, less-toxic preservatives (polyquaternium-1, polixetonium, sorbic acid, sodium perborate) may be considered for reducing ocular toxicity [136]. Preservative-free formulations are now being developed to avoid preservative-related irritation. Cationorm^®^ is a preservative-free emulsion formulated to enhance comfort and reduce visual blurring in patients suffering from DED [171]. Their ocular safety should be confirmed through in vitro, ex vivo, and in vivo evaluations. Additionally, as emulsions may alter tear film stability and cause dryness, their interactions with the ocular surface must be carefully assessed [40]. For novel ophthalmic emulsions, it is crucial to ensure transparency to prevent blurred vision, while also evaluating the potential for sensitization or allergic reactions.

### 16.4. Selection of Suitable Animal Models

Another major limitation lies in the availability of suitable animal models that exactly mimic human ocular physiology. Commonly used preclinical models, such as mice, rats, and rabbits, differ significantly from humans in terms of ocular anatomy and immune composition. For instance, rodents have smaller eyes with a higher lens-to-cornea ratio, while rabbits exhibit a thicker mucus layer, lower blink rate, and greater susceptibility to irritation. These interspecies differences lead to variations in ocular pharmacokinetics, making it difficult to extrapolate preclinical findings to human outcomes [1].

### 16.5. Sterilization Challenges

As noted above, sterilization is essential for ophthalmic emulsions and emulgels to ensure safety and prevent microbial infections, due to the high sensitivity of the eye. Maintaining sterility throughout production and storage is critical for product efficacy and patient safety. However, sterilization poses challenges, as methods like autoclaving may alter viscosity, droplet size, or stability of ophthalmic emulsions (lipid hydrolysis, drug degradation) [3], while filtration can be difficult for viscous formulations [3]. Alternative sterilization methods (gamma irradiation) should be adopted for effective sterilization of ophthalmic emulsions without compromising their stability [34].

### 16.6. Stability, Safety, and Sterility Maintenance

Sometimes emulsions experience stability problems outside the body (e.g., aggregation, flocculation, coalescence, creaming, Ostwald ripening, the expulsion of loaded drugs), as well as inside the body (premature drug release, degradation), which creates difficulty for long-term storage and clinical use. The agglomeration of globules over time alters their intrinsic properties and therapeutic efficacy [171,172]. The stability of emulsions is affected by properties of the dispersed phase (globule size, distribution), emulsifier/surfactants or cosurfactants (concentrations, type, HLB value), and dispersion medium (volume, viscosity, solubility) [171,172]. Use of an appropriate excipient within its permissible limit, with optimizing processing conditions, provides an emulsion with prolonged stability. Types of excipients, their proportions, and critical processing parameters can be optimized using a QbD approach [171]. Their sterility and safety must be established, not only for immediate use but also following chronic exposure. Furthermore, clinical suitability requires that these formulations are metabolized efficiently after exerting their therapeutic action, without accumulating in ocular tissues [40].

### 16.7. Manufacturing Scale-Up and Reproducibility

Emulsion development often involves complex, multi-step processes that pose challenges for large-scale manufacturing and industrial translation. Scale-up of nanoformulations is challenging due to batch-to-batch variations in attributes like particle size, PDI, zeta potential, and entrapment efficiency, which can impact product consistency and therapeutic efficacy. Implementing QbD and controlling critical material and process parameters can help achieve reproducible large-scale production of ophthalmic emulsions and emulgels.

### 16.8. Regulatory Constraints

Regulatory concerns for ophthalmic emulsions primarily emphasize ensuring safety, stability, sterility, and purity in compliance with international and pharmacopeial standards. Excipients used for ophthalmic emulsions must be safe, non-irritant, non-toxic, and sterile to prevent adverse effects on the ocular surface. The excipients must be previously approved for ophthalmic use, listed in the Inactive Ingredient Database of the FDA, or classified as GRAS. Additionally, supporting data on ocular tolerability and formulation stability are essential to obtain approval, ensuring the final product is both effective and safe for long-term patient use. Stringent regulatory guidelines create a bottleneck for the approval of ophthalmic emulsions.

### 16.9. Cost-Effectiveness and Clinical Translation

The cost-effectiveness of ophthalmic emulsions poses a significant challenge for development and clinical translation. Expensive high-quality excipients, advanced manufacturing procedures, sterilization requirements, specialized equipment for nanosizing, and thorough characterizations increase manufacturing costs, limiting large-scale commercialization. In addition to these, the complexity of scaling-up and an insufficient regulatory framework may increase the cost of novel formulations [173]. Cost optimization is therefore necessary for their successful clinical translation.

## 17. Future Perspective and Concluding Remarks

The treatment of ocular diseases often presents challenges due to the unique anatomical and physiological characteristics of the eye. Conventional ophthalmic drug delivery systems, such as eye drops, are limited by poor bioavailability, short retention times, and rapid ocular clearance. To address these limitations, novel therapeutic platforms such as NEs, MEs, SEDDSs, emulgels, and in situ-forming emulgels have emerged as promising solutions in ophthalmic drug delivery. These innovative drug delivery platforms represent significant advancements in ophthalmic therapeutics. By improving the solubility, bioavailability, and ocular retention of active pharmaceutical ingredients, these systems provide enhanced therapeutic outcomes, especially for drugs that are poorly water soluble.

To date, many emulsions have been developed. However, few of them have been translated into clinical use, or in some cases, even into animal model efficacy. Still, novel emulsions and emulgels with a high penetration ability, low dosing frequency, and improved ocular bioavailability are promising for treating eye diseases.

Research is underway to improve the ocular retention of drugs via novel emulsion systems (cationic emulsion, mucoadhesive), thereby improving ocular bioavailability. In situ gelling formulations will also gain more traction in the upcoming years [40]. To further improve the stability and therapeutic efficacy of ophthalmic products, co-encapsulating drugs with enzyme inhibitors can inhibit ocular enzymes that possibly cause the degradation of drugs, which can improve ocular bioavailability [1].

Clinical translation of ophthalmic emulsions faces several barriers, including challenges in large-scale production, reproducibility, long-term stability, and limited in vitro-–in vivo correlation, all of which hinder the transition from laboratory formulations to commercial products. The scale-up of nanoformulations remains particularly difficult, necessitating the adoption of industrially feasible and controlled manufacturing methods.

Achieving reproducibility is a major concern, as batch-to-batch variations during scale-up and multi-batch production can affect the consistency, quality, and therapeutic performance of ophthalmic emulsions and emulgels. To overcome these challenges, precise control of CMAs and CPPs, along with the integration of QbD principles, is essential to ensure the development of robust and reproducible ophthalmic formulations suitable for clinical translation.

Furthermore, the toxicity, ocular irritation, blurred vision, and instability have an impact on the clinical translation of emulsion-based topical ophthalmic formulations [171]. Patient compliance should be considered for the development of ocular formulations. Non-toxic products with negligible irritation and no blurring effect can maximize patient comfort and compliance. The emulsions should be formulated in such a way that they can offer prolonged drug release to minimize the dosing frequency. Detailed studies should be performed to understand their possible mechanism of uptake in ocular regions and the mechanism of action.

As noted above, newer, less-toxic preservatives should be preferred to minimize adverse effects (ocular toxicity, including irritation, dryness, and inflammation) of conventional preservatives. Preservative-free emulsions should be developed to avoid toxicity due to preservatives [136]. Surfactant-free ophthalmic Pickering emulsions can be prepared with other stabilizers to avoid surfactant-related irritation [171]. The toxicity due to surfactants can be reduced by selecting less toxic, non-irritant surfactants and/or reducing their concentration by incorporating non-toxic cosurfactants and cosolvents.

Advances in cationic and mucoadhesive emulsions have shown significant potential to increase ocular retention, bioavailability, and patient compliance by interacting with the negatively charged ocular surface and mucins [2]. These advanced emulsions enable prolonged drug release and reduce the dosing frequency.

Moreover, advanced formulation strategies offer promising opportunities for targeted drug delivery to the posterior segments of eye by employing surface modification, optimized nanocarrier design, and stimuli-responsive delivery systems.

Novel APIs also hold potential: ophthalmic emulsions incorporating bacteriophages, either alone or in combination with conventional antibiotics, are promising for combating antimicrobial resistance in the treatment of infectious ocular diseases [171].

Artificial intelligence (AI) is increasingly used in the development of novel dosage forms, including NEs, MEs, SNEDDSs, and SMEDDSs, to streamline formulation design. AI models can be employed in preformulation studies to select appropriate excipients, predict drug–excipient interactions, assess solubility, optimize formulations, predict drug release, forecast pharmacokinetics and pharmacodynamics, support quality control and characterization, and predict stability, shelf life, and clinical trial outcomes, thereby reducing trial-and-error experimentation [174,175,176].

Despite their promising benefits, challenges such as biocompatibility and toxicity, patient tolerability, sterilization, stability, scale-up, reproducibility, regulatory constraints, and clinical translation remain. Regulatory challenges remain significant for ophthalmic emulsions, requiring strict adherence to regulatory guidelines to ensure the safety, sterility, and biocompatibility of formulation components. Establishing standardized characterization methods and clear excipient safety and stability criteria will streamline regulatory approvals and facilitate smoother market entry. Nevertheless, these emerging emulsion and emulgel-based drug delivery systems hold great potential for improving the management of various ocular diseases, providing more effective, sustained, and patient-friendly treatment approaches.

## Figures and Tables

**Table 1:** Examples of ophthalmic emulsions for ocular diseases.

Type of Emulsion	Drug	Excipients	Preparation Method	Optimization Tool	In Vitro and In Vivo Experimental Model	Evaluated Therapeutic Use	Overall Outcomes	Reference
NEs	Travoprost	Lipophile oil Labrafac^®^ Tween^®^ 80	Low-energy method: Emulsion inversion point	NA	Sub-conjunctival suspension of 0.1% betamethasone-based glaucoma model in New Zealand white (NZW) rabbits Ocular irritancy on the eyes of NZW rabbits Pharmacokinetic study on NZW rabbit eyes	Glaucoma	Improved ocular absorption (C_max_, AUC) of NE compared to the Travatan^®^ marketed eye drops Found to be safe and non-irritant to ocular tissue The reduction in intraocular pressure (IOP) with Travatan^®^ eye drops was ended at 36 h, while the NE caused the reduction until 60 h The NE demonstrated a significant reduction in % IOP compared to commercial eye drop at 48 h and 60 h (*p* < 0.05)	[95]
NEs	β-caryophyllene	Medium-chain triglycerides (MCTs) Cetrimonium bromide (CTAB) Sorbitan monooleate 80 Polysorbate 80 Phosal^®^ 50 SA+	Spontaneous emulsification	NA	In vitro activity against *Acanthamoeba castellanii* trophozoites by AlamarBlue™ assay Ex vivo permeation across the porcine cornea ATR-FTIR for ex vivo corneal interaction	Acanthamoeba keratitis	The NEs showed significantly improved % inhibition compared to the isolated constituent NEs formulated with Phosal^®^ displayed less tissue interaction compared to CTAB-containing NEs The NEs showed improved permeation compared to plain drug	[96]
NEs	Brinzolamide and brimonidine tartrate	Polysorbate 80 Castor oil Glycerol Benzalkonium chloride Tris buffer	Spontaneous emulsification	Full factorial design	Preservative efficacy study against *Staphylococcus aureus* and *Pseudomonas aeruginosa* Hen’s egg test–chorioallantoic membrane (HET-CAM) for irritation study Ex vivo ocular permeability against goat cornea	--	Optimized NEs showed sufficient retention of preservative efficacy The NE was found to be non-irritating The formulation showed sustained drug release compared to Simbrinza^®^ NEs demonstrated improved permeation compared to marketed products	[97]
NEs	Itraconazole	Benzyl benzoate Eumulgin^®^ CO40 Propylene glycol	Spontaneous emulsification method	Mixture design	In vitro drug release In vitro antifungal activity against *Candida albicans*	Fungal infection	The NEs showed a seven-fold increase in the drug released compared to an aqueous suspension The antifungal activity of NEs was reported to be increased compared to drug suspension	[98]
NEs	Ciprofloxacin	Oleic acid Labrafac^®^ Lipophile WL 1349 Tween^®^ 80 Poloxamer 188	Hot homogenization and ultrasonication	--	Ex vivo transcorneal permeation across the rabbit cornea	Bacterial keratitis	The NEs showed sustained drug release They showed a 2.1-fold enhancement of permeation compared to the marketed ophthalmic solution	[99]
NEs	Luliconazole	Capryol 90 Ethoxylated hydrogenated castor oil Transcutol^®^ P	Gentle mixing	Central composite design	In vivo ocular irritation in NZW rabbit’s eye by Draize test In vivo ocular pharmacokinetics in rabbit eyes In vitro antifungal activity against *Fusarium* and *Aspergillus*	Fungal keratitis	The NEs demonstrated increased drug release compared to the drug suspension Significantly improved the in vitro antifungal activity over the drug suspension The NEs demonstrated good tolerance in rabbit eyes without any irritation Ocular pharmacokinetics showed improved bioavailability of NEs compared to drug suspension	[100]
NEs	Posaconazole	Isopropyl myristate Labrasol^®^ Transcutol^®^ P	Vortexing and stirring, followed by probe sonication	--	Ex vivo permeability study across goat cornea In vivo ocular irritation study in NZW rabbits by Draize test Antifungal activity against *C. albicans* and *A. niger* by well-diffusion assay	Fungal eye infection	The NEs demonstrated improved drug release compared to drug suspension NEs showed improvement in drug permeation across the goat cornea compared to drug suspension Very negligible irritation (conjunctival redness) after 3 h of instillation was observed, and it was found to decrease and disappear gradually In vitro antimycotic inhibitory activity was found to be increased in the case of NEs compared to a drug suspension	[101]
NEs	Besifloxacin	Triacetin Cremophor^®^ RH 40 Transcutol^®^ P	Low-energy emulsification	--	HET-CAM for irritation study Ex vivo transcorneal permeation study through bovine cornea In vitro antimicrobial activity against *P. aeruginosa* and *S. aureus*	Bacterial infection	NEs demonstrated a sustained release profile NEs demonstrated 1.7-fold higher transcorneal permeation compared to drug suspension The HET-CAM showed no sign of irritation Comparative antimicrobial efficacy of NEs (0.2%) with 0.6% drug suspension	[102]
NEs	Isoliquiritigenin	Propylene glycol dicaprylate Cremophor^®^ EL Polyethylene glycol 400 Sodium hyaluronate	Water titration method	Central composite design	Ex vivo corneal permeation study across freshly isolated rabbit corneas Cytotoxicity against human corneal epithelial cells by CCK-8 assay Ocular irritation study and pharmacokinetic study in NZW rabbit’s eye Alkali burn (NaOH solution)-based corneal NV in BALB/c mice eyes	Corneal neovascularization	The NEs showed significantly higher drug release and permeation than drug suspension The NEs showed no cytotoxicity in human corneal epithelial cells and showed no irritation in rabbit eyes NEs showed bioavailability of 5.76-fold, 7.80-fold, and 2.13-fold higher than drug suspension in tears, cornea, and aqueous humor, respectively The efficacy of NE treatment (0.2% ISL) was found to be well comparable to that of dexamethasone (0.025%) in the inhibition of corneal NV in the mouse model	[103]
NEs	Curcumin	Vegetable oil Polyethylene glycol 400 Polysorbate 80 Alpha-tocopherol acetate Ascorbic acid	Self-nano emulsification method	--	Atropine-induced dry eye in Balb/c mice	DED	All the NEs demonstrated increased tear production and maintained it until the end of the study (day 49) compared to the atropine group	[104]
NEs	Fluconazole	Oleic acid Kolliphor EL Tween^®^ 80 Tween^®^ 20 Pluronic^®^ F127 Polyethylene glycol 200 Propylene glycol	High-pressure homogenization	--	In vitro antifungal activity against *C. albicans*, *C. parapsilosis*, *C. glabrata*, and *C. tropicalis* by disk diffusion method	--	The NEs showed equivalent antifungal activity with 0.3% aqueous drug solution The NEs demonstrated prolonged drug release compared to a control	[105]
Cationic NEs	Cyclosporin A	Castor oil, chitosan, Poloxamer 188, glycerin	Magnetic stirring followed by high-shear homogenization	Screening of factors by Taguchi OA design and optimization using central composite design	Cytotoxicity and cellular uptake study using human corneal epithelial cells (HCE-2) HET-CAM for irritation study Ocular biodistribution study in albino rabbit eyes	--	The NEs showed no cytotoxicity in HCE-2 The cellular uptake study of coumarin-loaded NEs showed improved uptake compared to coumarin suspension The HET-CAM showed no sign of irritation The biodistribution study showed therapeutic drug level (50–300 ng/mL) at 90 min post-topical administration The spray-dried ophthalmic emulsion can be stored and reconstituted for ocular administration The NEs showed enhanced anti-inflammatory activity compared to diclofenac reference.	[106]
Cationic NEs	Rifampicin	Oleic acid Polysorbate 80 Poloxamer 188 Chitosan Polymyxin B	High-pressure homogenization	2^3^ full factorial design	In vitro 3-(4,5-dimethylthiazol-2-yl)-2,5-diphenyltetrazolium bromide (MTT) assay against *Mycobacterium tuberculosis*	Ocular tuberculosis	Demonstrated in vitro mucoadhesion property The surface modifications with cationic substances did not alter the antimicrobial activity of rifampicin	[107]
Cationic NEs	Dorzolamide hydrochloride	Isopropyl myristate Tween^®^ 80 Cetyl trimethyl ammonium bromide	High-speed homogenizer	Box–Behnken design	Glaucoma model on male NZW rabbits Draize test for ocular irritancy assessment using NZW rabbits	Glaucoma	NEs showed a sustained release profile compared to plain drug solution The formulation showed in vitro mucoadhesive properties compared to the marketed eye drops and drug solution It showed excellent thermodynamic stability The cationic NEs showed improved and extended lowering effect of IOP in albino rabbits compared to plain drug solution and marketed eye drops The NEs were found to be safe and non-irritant for topical ocular use	[108]
Cationic NEs	Prednisolone	Tween^®^ 80 Propylene glycol Cremophor^®^ RH40 Polyethylene glycol 600 Glycerin Cetalkonium chloride	Mixing followed by probe sonication	--	Ex vivo corneal permeability across rabbit cornea Bovine serum albumin-induced uveitis model on NZW rabbit eye	Uveitis	The cationic NEs demonstrated sustained drug release The cationic NEs showed enhanced flux through rabbits’ corneas compared to free drug suspension and NEs without adding cationic surfactant The cationic NEs significantly decreased the severity of uveitis in rabbits’ eyes compared to the drug suspension NEs showed excellent ocular safety without causing any adverse effects on the eye or an increase in IOP	[35]
Mucoadhesive NEs	Moxifloxacin	Oleic acid Tween^®^ 80 Glycerin HPMC K4M PVP K29/32	Hot homogenization coupled with probe sonication	--	Ex vivo transcorneal permeation across the rabbit eye Antimicrobial activity against methicillin-resistant *S. aureus*	Ocular bacterial infections	The NEs demonstrated sustained release properties for a period of more than 12 h Transcorneal permeation studies of NEs demonstrated a 2.1-fold improvement in drug permeation compared with Vigamox^®^ eyedrops The NEs and mucoadhesive NEs displayed similar in vitro antibacterial activity as the drug solution and commercial eyedrop	[84]
SNEDDSs	Triamcinolone	Castor oil Polysorbate 80 PEG 400 Polyoxyethylene 35 Glycerol Propylene glycol	Low-energy method: Self-nanoemulsion method	Manual optimization	MTT assay on RPE cells In vitro cellular uptake study on RPE cells In vivo ocular irritation study	--	Safety of the SNEDDS towards RPE cells Efficient in vitro uptake of coumarin 6-loaded SNEDDS in RPE cells Excellent in vivo ocular biocompatibility without any sign of histopathological alterations in the sclera, cornea, retina, and optic nerve The SNEDDS showed their ability to penetrate the retina, choroid, and sclera Showed the potential for the delivery of the drug to the posterior segment of the eye	[77]
SNEDDSs	Resveratrol melatonin	Capryol^®^ Tween^®^ 80 PGMC Transcutol^®^ P	Simple mixing	Simplex lattice design for ternary-phase diagrams and I-optimal design for formulation optimization	In vitro cytocompatibility on SIRC cells by MTT assay	--	The formulation showed excellent pharmaceutical properties (size, emulsification time, and % transmittance), stability, and mucoadhesive properties The SNEDDSs showed excellent cytocompatibility with corneal epithelium cells and their potential for ocular administration	[81]
MEs	Dexamethasone sodium phosphate	Capryol^®^ PGMC D-α-tocopherol polyethylene glycol succinate (TPGS) Plantacare^®^ (coco-Glycosides)	--	Manual optimization	Endotoxin-induced uveitis rat model Ex vivo trans corneal permeation via bovine eyeballs Ex vivo mucoadhesion in bovine cornea Ocular irritancy by Draize test using rabbit eyeballs	Uveitis	Draize test demonstrated the ocular compatibility of the ME The ME showed excellent mucoadhesion and transcorneal permeation compared to aqueous solution Confocal laser scanning microscopy (CLSM) showed deeper permeation into the rabbits’ corneas Significant reduction in inflammatory markers (interleukin-6 (IL-6) and tumor necrosis factor-α (TNF-α))	[34]
MEs	Gatifloxacin	Isopropyl myristate (IPM) Tween^®^ 80 Transcutol^®^ P	Spontaneous emulsification with simple vortexing	NA	Ex vivo transcorneal permeability across goat cornea In vivo ocular irritation on NZW rabbits by Draize’s test Corneal retention by gamma scintigraphy and pharmacokinetic studies on NZW rabbits Antimicrobial activity using agar diffusion assay against *Bacillus subtilis*, *S. aureus*, and *Escherichia coli*	Ocular bacterial infections	MEs showed excellent stability It demonstrated greater adherence to the corneal surface The marketed formulation showed improved transcorneal permeability (steady-state flux, permeability coefficient) Gamma scintigraphy showed significantly improved retention of the radioactive tracer in the case of MEs compared to marketed eye drops It showed two-fold improved ocular bioavailability compared to the conventional commercial Zigat^®^ eye drops Showed excellent antimicrobial activity similar to the reference solution (Zigat^®^ eye drops) Demonstrated non-irritancy and excellent ocular tolerability	[109]
MEs	Quercetin	Oleic acid Transcutol^®^ P Span^®^ 20 Tween^®^ 80 Propylene glycol	Spontaneous emulsification: Stirring	Full factorial design	Ex vivo corneal permeation across NZW rabbits’ eyes	--	Showed significantly improved transcorneal permeation (improved flux and diffusivity coefficient) compared to aqueous suspension The MEs showed interaction with the corneal structure and improved corneal permeation	[110]
MEs	*Cineraria maritima* extract	Ethyl oleate Tween^®^ 80 Span^®^ 20	Water titration method	--	In vivo anticataract on intravitreally injected sodium selenite-induced NZW rabbit model Ocular irritation studies in NZW rabbit model by Draize test Ex vivo permeability across goat cornea	Cataract	MEs significantly improved ocular permeation compared to the aqueous marketed formulation The MEs showed their potential for topical ocular administration without any ocular irritation The anti-cataract efficacy of MEs was found to be significantly improved compared to the marketed product	[37]
MEs	Naringenin	Triacetin Cremophor^®^ RH40 PEG400	Spontaneous emulsion method	Central composite design	Ex vivo corneal permeation Toxicity study against Human corneal epithelial cells (HCECs) Ex vivo corneal permeation study on rabbit corneas Ocular irritation study on NZW rabbits using Draize test Ocular pharmacokinetic study on NZW rabbits Alkali (NaOH solution)-induced corneal NV mice model	Corneal neovascularization	MEs showed increased drug release and corneal permeation compared to the drug suspension The formulation showed no toxicity to HCECs It showed no signs of ocular irritation MEs demonstrated significantly improved ocular bioavailability (1.35–2.15-fold) compared to drug suspension The MEs showed significantly higher corneal drug permeation compared to drug suspension MEs demonstrated attenuation of corneal VEGF and MMP-14 expression MEs showed comparable efficacy to that of dexamethasone for the inhibition of CNV	[111]
MEs	Sunitinib	Oleic acid Cremophor^®^ RH40 Transcutol^®^ P Sodium hyaluronate	Phase inversion emulsification method	--	In vitro cytotoxicity to human corneal epithelial cells Ocular irritation by Draize test, ocular pharmacokinetics on NZW rabbits Inhibitory effects on CNV were evaluated in vitro and in vivo Alkali (NaOH solution) burn-induced corneal NV in BALB/c mouse model	Corneal neovascularization	The MEs displayed a sustained release profile The formulation demonstrated no clear toxicity against HCECs The MEs showed an ocular irritation score of 0, representing no ocular irritancy The MEs 2.47 and 2.14-fold improved corneal and conjunctival bioavailability, respectively It suppressed the alkali burn-induced CNV in mice, and at a high dose (0.1%), the formula showed a similar efficacy to dexamethasone (0.025%)	[112]
MEs	Sorafenib	Proprietary drug delivery system (NaMESys)	--	--	In vitro cytotoxicity on SIRC cells In vivo ocular tolerability study in NZW rabbits by Draize test Efficacy study in Brown Norway adult rats retinal ischemia–reperfusion injury model; streptozotocin-induced DR model in Sprague–Dawley rats; and CNV model on C57Bl6/J mice	Diabetic retinopathy	The MEs showed in vitro cytocompatibility on corneal cells of rabbits, and they were well-tolerated following b.i.d. topical ocular administration The MEs significantly inhibited retinal expression of TNF-α (20.7%) and inducible nitric oxide synthase (iNOS) (87.3%) mRNAs compared to controls MEs inhibited retinal expression of NF-κB, TNF-α, GF1, IGF1 receptor, VEGFR1, and VEGFR2 mRNAs in streptozotocin-induced diabetic rats, by 3-fold compared to controls The ophthalmic MEs showed significant inhibition of neovascular lesions by 54% in the laser-induced CNV model	[113]
MEs	Ketorolac tromethamine	Oleic acid Transcutol^®^ P Span^®^ 20 Tween^®^ 80 Propylene glycol	--	Full factorial design	Corneal permeation across the rabbit cornea	--	The MEs significantly increased the cornea permeation compared to ketorolac drops	[114]
MEs	Osthole	Capryol^®^ 90 Cremophor^®^ EL Transcutol^®^ P Sodium hyaluronate	Phase inversion emulsification	Central composite design	In vitro cytotoxicity against HCECs by CCK-8 assay In vivo ocular irritation on NZW rabbit eye by Draize eye test Ocular pharmacokinetics in rabbits Corneal NV mouse model using BALB/c mice by the alkali burn (NaOH solution) method	Corneal neovascularization	The MEs were found to be non-toxic to HCECs It also demonstrated good ocular biocompatibility without causing any irritation to the rabbit eye The MEs showed 28.2 and 102.34-fold higher pharmacokinetic profiles (AUC_0–t_) in cornea and conjunctiva compared to the drug suspension The osthole-loaded MEs (0.1%) showed a similar therapeutic effect to marketed dexamethasone eye drops (0.025%) on CNV in mouse models	[115]
MEs	Triamcinolone acetonide	Oleic acid Cremophor^®^ EL Propylene glycol	Simple mixing	--	In vivo ocular irritation on domestic rabbits using the Draize eye test Bovine serum albumin-induced Uveitis rabbit model	Uveitis	The developed MEs was found to be non-irritant to the rabbit eye The MEs showed improved therapeutic efficiency for the uveitis treatment compared to the marketed suspension	[36]
MEs	Acetazolamide	Castor oil Olive oil Tween^®^ 80	Simple mixing	--	Dexamethasone-induced ocular hypertension in NZW male albino rabbits Ocular irritation study on rabbit’s eye using phenol red thread test	Glaucoma	MEs showed sustained drug release compared to the drug solution It showed a significant IOP reduction The MEs showed two three-fold increases in AUC_0–10_ compared to the drug solution The MEs showed a prolonged duration of action (10 h) compared to aqueous solution (4–5 h) Slight redness initially, which gradually reduced, and complete recovery was observed at 24 h	[42]
MEs	Brinzolamide	Isopropyl myristate Tween^®^ 80 Transcutol^®^ P	Water titration method: Simple stirring	D-optimal mixture design	Ocular irritation study on isolated goat cornea by histopathology	Glaucoma	The MEs showed prolonged drug release The MEs were found to be non-irritant and safe for ocular application	[43]
Mucoadhesive MEs	Ganciclovir	Capmul MCM EP Labrasol^®^ Transcutol^®^ P Chitosan	Water titration method	--	Ex vivo goat corneal permeation In vitro cytotoxicity against SIRC and ARPE-19 by Alamar Blue assay Ex vivo corneal irritation study on goat cornea by histopathological observation In vitro mucoadhesion measurement by zeta potential and ex vivo mucoadhesion by spectroscopy method on goat cornea	--	The chitosan-based MEs showed improved drug permeation across the goat cornea compared to drug solution and plain o/w or w/o MEs The chitosan-based MEs showed higher mucoadhesion compared to other formulations The in vitro cytotoxicity showed the ocular compatibility of the MEs Histopathological observation of the cornea also confirmed the retention of corneal architecture and the non-irritant nature of the developed MEs	[116]
Cationic MEs	Clotrimazole	Oleic acid Transcutol^®^ HP Cremophor^®^ EL Chitosan	Spontaneous emulsification with stirring	2^2^ × 3^1^ full factorial design	In vivo ocular tolerance and histopathological studies of male albino rabbits *Candida albicans* susceptibility test	Fungal keratitis	In vivo ocular tolerance revealed ocular safety (no sign of redness, lacrimation, or inflammation) of MEs for topical application The histopathological studies revealed no abnormalities in retinal tissues The MEs showed sustained antifungal activity for a prolonged period compared to the drug suspension The chitosan-coated cationic ME showed prolonged ocular retention due to its mucoadhesive nature	[30]
PEGylated MEs	Triamcinolone Acetonide	Capmul MCM C8 AccononMC8-2 Transcutol^®^ DSPE-PEG 2000	Water titration	--	Ocular biocompatibility using RBCs, HET-CAM study, histopathology study In vitro cytotoxicity study against SIRC and ARPE-19 cells Transepithelial electrical resistance In vivo pharmacokinetic study on Sprague–Dawley rats	--	The MEs displayed a sustained release profile compared to the solution The formulation was isotonic with tear fluid and showed excellent ocular biocompatibility without any sign of histopathological alterations, in vitro cytotoxicity, and vascular alterations in HET-CAM study The MEs showed a decrease in TEER value The MEs showed prolonged ocular retention and release, crossed ocular barriers, and reached the retina easily with a high amount compared to the solution	[117]
PEGylated MEs	Dexamethasone	Capmul MCM EP Tween^®^ 80 Kolliphor RH40 Labrasol^®^ DSPE-PEG 2000 Transcutol^®^ HP		NA	Ocular biocompatibility using RBCs, HET-CAM study, histopathology study In vitro cytotoxicity study against SIRC and ARPE-19 cells Transepithelial electrical resistance on SIRC cells Pharmacokinetic study on Sprague–Dawley rats	--	Demonstrated sustained drug release The MEs showed cytotoxicity at higher concentrations; however, there was negligible toxicity after dilution The MEs showed a decrease in TEER value; however, no significant decrease was noticed compared to the control group The in vivo ocular pharmacokinetic study demonstrated improved drug retention in the retina compared to the fluorescent-labeled simple eye drop solution	[118]
Bioadhesive Multiple MEs	Ribavirin	Capryol 90 Labrafac^®^ Lipophile WL1349 Labrasol^®^ Soybean lecithin Cremophor^®^ EL Propylene glycol	Simple vortexing	NA	In vitro cell viability of human corneal limbal epithelial cells (HCLEs) by MTT assay Transcorneal permeability against NZW rabbit eyes In vivo ocular tolerance on Dutch belted rabbit eye by modified Draize test	--	The MEs showed a sustained release profile for up to 24 h Formulation improved the corneal permeability by 3-fold The developed MEs showed excellent in vitro biocompatibility The acute and chronic ocular toxicity revealed excellent tolerability to the eye without causing any irritation	[67]
In situ gelling MEs	Moxifloxacin and betamethasone	Ethyl oleate Cremophor^®^ EL Plurol^®^ oleique Poloxamer 407	Ultrasonication	NA	Ex vivo ocular penetration using porcine cornea	Intraocular surgery	Showed in situ gelling property at the ocular temperature Demonstrated controlled drug release compared to the drug solution Improved corneal penetration via porcine corneas compared to control solution	[71]
In situ gelling MEs	Sparfloxacin	Labrafil M1944 CS Acrysol–140 Transcutol^®^ P Poloxamer 407	Spontaneous emulsification	Simplex lattice design	HET-CAM eye irritation study In vitro antimicrobial efficacy study via cup plate (agar plate) method	DED and corneal ulcer	Showed in situ gelling property at the ocular temperature Sustained drug release for more than 10 h Non-irritant Showed improved drug permeation across the goat cornea compared to the marketed eyedrop solution	[15]
Thermoresponsive in situ gel of NEs	Acyclovir	Triacetin as oil Transcutol^®^ P Poloxamer 407 Poloxamer188	Low-energy method: Stirring	--	Ex vivo transcorneal permeation using bovine eye Ocular irritation test in NZW rabbit eye using Draize test HET-CAM test for ocular irritancy	Herpes simplex keratitis infection	The NEs demonstrated sustained drug release compared to the drug solution The in situ nanoemulgel showed 2.8-fold improved permeation compared to the drug solution The in situ gel showed excellent ocular tolerability without any irritation	[93]
Thermosensitive in situ gelling NEs	Besifloxacin	Triacetin Cremophor^®^ RH 40 Transcutol^®^ P Poloxamer 188 Poloxamer 407	Spontaneous emulsification method	--	HET-CAM test for ocular irritancy Ex vivo transcorneal permeation across bovine cornea Antibacterial efficacy against *P. aeruginosa* and *S. aureus* by agar diffusion test	Ocular bacterial infections	Non-irritancy of the optimum in situ gel NEs The NEs showed prolonged retention on the eye surface Showed remarkable efficacy against microbes and its possible use for ocular bacterial infections	[119]
In situ gel NEs	Brinzolamide	Triacetin, Transcutol^®^ P Tween^®^ 80 Poloxamer 188 Pluronic^®^ 407	Mixing and stirring	--	In vitro cytotoxicity by MTT assay on the human RPE cells HET-CAM study for ocular irritancy In vivo ocular irritation by modified Draize method in NZW rabbit eye In vivo therapeutic efficacy study in NZW rabbits in terms of maximum IOP decrease rate (E_max_%), area under the curve (AUC_0–6h_), and time to achieve maximum IOP reduction (T_max_)	Glaucoma	The HET-CAM results for in situ NE gel showed no sign of blood vessel injury in chorioallantoic membrane The NEs were well-tolerated by rabbit eyes without any ocular irritation Showed significantly improved pharmacodynamic results (increased E_max_%, shorter T_max_, and increased AUC_0–6h_) compared to drug suspension	[41]
Gel-in-Water NEs		Beeswax dissolved in castor oil Polyoxyethylene hydrogenated castor oil-60	Ultrasonication	--	In vitro biocompatibility against rat hepatocytes and human umbilical vein endothelial cells (HUVECs) In vivo ocular irritation on ICR mice using the Draize technique In vivo corneal permeability using Institute of Cancer Research (ICR) mice	--	The NEs showed improved stability and retinal permeability The gel in water NEs were found to be biocompatible with HUVECs and hepatocytes The NEs showed no sign of in vivo ocular irritation It showed improved ocular permeation compared to the control	[120]
Microemulsion ocular gel	Prednisolone acetate	Oleic acid IPM Tween^®^ 80 Propylene glycol Ethanol Carbopol 934 Benzalkonium chloride	--	--	Ex vivo ocular permeation across sheep cornea	--	The microemulgel was found to be non-irritant and compatible with the eye The formulation showed excellent drug–excipient compatibility, optimum viscosity, homogeneity, and spreadability The microemulgel showed a two-fold increase in corneal drug permeation compared to the marketed ointment	[121]
In situ emulgel	Levofloxacin	Sunflower oil Tween^®^ 80 Span^®^ 80 Chitosan, HPMC, poloxamers, gellan gum, sodium alginate	Stirring	--	Ex vivo drug permeation across goat cornea In vitro anti-microbial activity against *S. aureus* and *E. coli*	Ocular bacterial infections	Demonstrated prolonged drug release Showed enhanced residence time	[87]
Emulgel	Levofloxacin and betamethasone	Castor oil Poloxamer 188 Xanthan gum Carbopol 934 Methyl cellulose Glycerin	Stirring	--	In vitro antibacterial activity against *E. coli* and *S. aureus*	Bacterial eye infections	Emulgel showed a simultaneous and extended-release pattern of the two drugs The emulgel showed its potential for co-loading both hydrophilic and hydrophobic drugs in the same formulation without the need to use two drops, offering excellent patient compliance	[122]
Emulgel	Acetazolamide	Corn oil Tween^®^ 80 Span^®^ 80 Pectin Gellan gum	Homogenization	--	Ex vivo corneal permeability across the goat cornea In vivo glaucoma study in rabbits using 5% glucose Ocular irritation study using albino rabbits by Draize parameters	Glaucoma	The cumulative percentage drug release was found to be increased with increasing oil content Significant reduction in intraocular pressure after topical ophthalmic emulgel application All the emulgel formulations are non-irritant to the eye without any sign of conjunctival redness, chemosis, or corneal opacity	[123]

ARPE-19: adult RPE cells; CCK-8: Cell Counting Kit-8; CLSM: confocal laser scanning microscopy; DED: dry eye disease; DSPE-PEG 2000: Distearoylphosphatylethanolamine-polyethyleneglycol 2000; HCECs: human corneal epithelial cells; HCLE: human corneal limbal epithelial cell line; HET-CAM: hen’s egg test-chorioallantoic membrane; ICR: Institute of Cancer Research; IGF: insulin-like growth factor 1; IGF1 receptor: insulin-like growth factor 1 receptor; iNos: inducible nitric oxide synthase; MMP-14: matrix metalloproteinase expression; NF-κB: nuclear factor kappa B; NZW: New Zealand white; SIRC: Statens Seruminstitut rabbit cornea; TNF-α: tumor necrosis factor-alpha; VEGF: vascular endothelial growth factor; VEGFR1 and VEGFR2: vascular endothelial growth factor receptors 1 and 2.

**Table 2:** Key characterization parameters, acceptance criteria, and their relevance to ophthalmic performance of ophthalmic emulsions and emulgel.

Characterization Parameter	Acceptance Criteria	Relevance to Ophthalmic Performance	References
pH	Close to pH of tear fluid (pH 7.4) Acceptable range: pH 6.5–8.5	Minimizes irritation and maximizes patient compliance	[16,17]
Viscosity	Viscosity within 15–150 mPa·s	Optimum viscosity allows prolonged ocular retention and maximizes the absorption	[16]
Osmolarity	Should be close to the osmolarity of tear fluid: 270–310 mOsm/L	Minimizes irritation, retains ocular tissue integrity, and maximizes patient compliance	[16]
Surface tension	Within 40–50 mN/m	Influences drop size, amount of drug/dose, spreading behavior, tear film stability, and ocular comfort	[3,4,17]
Refractive index	A very close RI value to that of tear fluid (1.340 to 1.360) or a value less than 1.476	Affects the transparency/visual clarity, isotropy, and optical compatibility	[3,17]
Transmittance	A value greater than 98%	Minimizes blurring or haziness and offers clear vision and visual comfort after instillation	[17]
Globule size	Below 200 nm	Allows effective drug permeation across ocular barriers by receptor-mediated endocytosis	[84]
Polydispersity	Below 0.3	Homogeneity and narrow size distribution/particles of uniform size	[84]
Zeta potential	Greater than ±30 mV	Offers more repulsive force between globules, avoids aggregation/coalescence, and offers prolonged stability	[84]
Drug content and content uniformity	Content within 85–115% (±15%)	Offers dose accuracy and therapeutic consistency and maintains product efficacy	[16]
Entrapment efficiency	In general, high entrapment efficiency (>90%)	Offers improved ocular bioavailability and reduces dosing volume and dosing frequency	
Loading capacity	In general, high loading capacity	Necessary to load maximum drug in limited amount of excipients that ultimately reduce excipient-related issues (irritation or toxicity)	
Drug–excipient compatibility	No shifting or disappearance of characteristic spectra (vibrational or thermal peaks) of drug	Necessary to observe possible interactions between the selected excipients and the drug	[34,37,112,117]
Stability	Should remain stable in the prescribed storage conditions for prolonged time period	Ensures safety, efficacy, and quality throughout the product’s shelf life	[139]
Transcorneal/scleral permeability	Should permeate required quantity of drugs to achieve therapeutic activity	Improves ocular drug bioavailability and therapeutic efficacy	[95,99]
Ocular biocompatibility, toxicity, and irritancy	Should be non-toxic, non-irritant, and biocompatible with ocular tissue	Ensures safety, improved tolerability, and regulatory compliance	[3]
Ocular pharmacokinetics	Formulation should provide improved pharmacokinetic profile compared to drug solution or suspension	Provides understanding on drug absorption, distribution, and clearance in ocular tissues	[103,111,112]
Sterility	Should be completely free from microbial contaminants	Ensures the absence of microbial contamination	[16,117]
Presence of pyrogens	Should be free from pyrogens (metabolic byproducts of microorganisms)	Ensures safety and regulatory compliance	[16]

**Table 3:** Recent patents on ophthalmic emulsions and emulgels, 2015–2024.

Patent Title	Description	Patent No.	Publication Date	Type of Formulation	Therapeutic Use/Disease Target	Reference
Nanoemulsion ophthalmic composition comprising cyclosporine and menthol, and preparation method thereof	Provides a nanoemulsion ophthalmic composition comprising cyclosporine with improved stability, bioavailability, and eye irritation, as well as a method for preparing the same.	US20240041974A1	8 February 2024	o/w NEs	DED	[148]
Ophthalmic preparations	Ophthalmic formulations containing cyclosporine and methods for preparing the formulation.	US11173112B2	16 November 2021	o/w NEs	DED	[149]
Self-emulsifying drug delivery (SEDD) for ophthalmic drug delivery	Ophthalmic self-emulsifying systems with their methods of preparation and their use for delivering poorly water-soluble drugs.	US20240307299A1	19 September 2024	SEDDSs	Ocular drug delivery	[150]
Ophthalmic emulsion	The emulsion includes a mucoadhesive polymer (galactomannan polymer) that aids in delivering a lipid to the ocular surface.	US11690802B2	4 July 2023	o/w emulsion with mucoadhesive polymer	DED	[151]
Ophthalmic emulsion	Provides a process for producing a stable emulsion with a small mean droplet in the presence of a mucoadhesive polymer.	US11234929B2	1 February 2022	o/w emulsion	DED	[152]
Ophthalmic drug delivery system	Ophthalmic drug delivery system comprising microemulsion and liposome nanodroplet-laden contact lens.	US8273366B2	25 September 2012	o/w MEs	Ophthalmic drug delivery	[153]
Microemulsion for ophthalmic drug delivery	Formulation for ophthalmic delivery of a therapeutic agent and its use for the treatment of ocular conditions.	US20210085603A1	25 March 2021	o/w MEs	Ophthalmic drug delivery	[154]
Ophthalmic preparations	Ophthalmic formulations of cyclosporine, methods for formulation development, and their use	US20190076354A1	16 November 2021	o/w NEs and cationic emulsion	DED	[155]
Eye composition containing a cyclosporine and a method of preparing the same	Nanoemulsion ophthalmic formulation in which the solubility of cyclosporine is increased and the stability of the ophthalmic composition is improved.	KR102204221B1	18 January 2021	NEs	DED	[156]
Ophthalmic compositions and methods of use	Provides a method for using ophthalmic composition to treat an eye disorder.	US10555947B2	11 February 2020	o/w NEs	DED	[157]
Nanoemulsion compositions with enhanced permeability	Nanoemulsion compositions that are administered topically, mucosally (intranasal, ocular, oral, vaginal), intravaginally, or intranasally and have enhanced permeability.	JP7642536B2	10 March 2025	o/w NEs	DED	[158]
Compositions of nanoemulsion delivery systems	Enhanced drug delivery using topical, ocular, and transdermal routes.	KR102407735B1	10 June 2022	NEs	DED	[159]
Cyclosporine-containing, non-irritative nanoemulsion ophthalmic composition	Ophthalmic emulsion containing cyclosporine for dry eye disease.	AU2013255231B2	13 August 2015	NEs	DED	[160]
Methods and compositions for treating dry eye disease and other eye disorders	Ophthalmic formulations containing an alpha 2 adrenergic agonist for the treatment of ocular disorders, including dry eye syndrome and Meibomian gland dysfunction.	US9597328B2	25 August 2016	o/w NEs	DED	[161]
Emulsion formulation of multikinase inhibitor	The use of multikinase inhibitors, such as nintedanib, axitinib, or pazopanib, and the compositions are emulsions, such as NEs, for topical administration to the eye to treat diseases affecting the anterior eye segment.	JP7489965B2	24 May 2024	NEs	Diseases affecting the anterior segment	[162]
An ophthalmic flurbiprofen ester nanoemulsion in situ gel formulation and the preparation method thereof	Preparation method of the flurbiprofen axetil ophthalmic nanoemulsion—in situ gel preparation.	CN102159186B	20 May 2015	NE-based in situ-forming gel	Anterior segment inflammation	[163]
Self-emulsifying drug delivery systems (SEDDSs) for ophthalmic drug delivery	The self-emulsifying non-aqueous formulation and its use and preparation method are described.	JP2021107402A	22 July 2022	SNEDDSs	Ocular drug delivery	[164]
Ophthalmic compositions for the administration of liposoluble active ingredients	Ophthalmic microemulsions or self-emulsifying systems for the administration of lipophilic active ingredients on the ocular surface.	EP2579845B1	29 April 2020	MEs and self-emulsifying systems	Ocular drug delivery	[165]
Compounds and formulations for treating ophthalmic diseases	Formulation compositions and methods of use thereof in the treatment and prevention of ocular conditions, like cataracts and presbyopia.	US20180250313A1	6 September 2018	o/w NEs	Cataract and presbyopia	[166]
Pharmaceutical oil-in-water nanoemulsion	Pharmaceutical oil-in-water nanoemulsion compositions for enhancing the delivery of lipophilic drugs and a process for preparing said compositions.	JP2020037557A	12 March 2020	o/w NEs	Reducing intraocular pressure	[167]
Ophthalmic compositions comprising castor oil and medium-chain triglycerides	Ophthalmic compositions and their use for the treatment of eye diseases.	ES2706535T3	29 March 2019	o/w NEs	Ocular drug delivery	[168]
Sustained-release ophthalmic formulation and methods for using the same	Provides methods for treating dry eye syndrome using a sustained release o/w nanoemulsion and alpha 2 adrenergic agonists, pharmaceutically acceptable salt, or a mixture thereof.	US20180221278A1	9 August 2018	o/w NEs	DED	[169]
Compositions and methods for treating ophthalmic conditions	Provides methods of treating ophthalmic conditions in a patient, such as dry eye disease, inflammation, pain, or conjunctivitis.	US20230293557A1	21 September 2023	MEs	DED, inflammation, pain, or conjunctivitis	[170]

DED: dry eye disease.

**Table 4:** Recent clinical trials on ophthalmic emulsions and emulgels.

Clinical Trial No.	Study Design/Allocation	Total Number of Participants	Active Pharmaceutical Ingredient	Type of Formulation	Phase of Clinical Trial	Therapeutic Use/Disease Target
NCT03785340	Randomized	252	Brimonidine tartrate	NEs	Phase 3	DED
NCT04246801	Randomized	211	Clobetasol propionate	NEs	Phase 3	Inflammation and pain associated with cataract surgery
NCT04249076	Randomized	215	Clobetasol propionate	NEs	Phase 3	Inflammation and pain associated with cataract surgery
NCT04426240	Randomized	40	Cyclosporine	MEs	Phase 4	Prevention of post-cataract surgery dry eye syndrome
NCT04918823	N/A	10	Cyclosporine	Emulsion-based device	Phase 1 Phase 2	Application of prosthetic replacement of the ocular surface ecosystem (PROSE) lens reservoir for the management of patients with ocular surface disease
NCT00827255	Observational	35	Cyclosporine	Emulsion	Observational	DED
NCT06144918	Randomized	56	SBI-100	Emulsion	Phase 2	Elevated intraocular pressure
NCT01254370	Randomized	105	Latanoprost Travatan Z	Emulsion	Phase 2	Glaucoma and ocular surface disease
NCT04812951	Randomized	101	Cyclosporine	Emulsion	Early phase 1	Prophylactic treatment in cataract surgery
NCT01368198	Randomized	50	Systane balance lubricating eye drops	Emulsion	N/A	Dry eye disease
NCT00406887	Randomized	140	Difluprednate	Emulsion	Phase 3	Anterior uveitis (including panuveitis)
NCT01207752	Randomized	69	Systane balance	Emulsion	N/A	Meibomian gland dysfunction
NCT00407056	Non-randomized	20	Difluprednate	Emulsion	Phase 3	Severe uveitis
NCT01109056	Randomized	115	Cyclosporine	Emulsion	Phase 2	Pterygium
NCT03693989	Randomized	178	Difluprednate	Emulsion	Phase 3	Inflammation and pain after phacoemulsification
NCT06174181	Randomized	42	Preservative-free ophthalmic lubricant	Emulsion	N/A	Dry eye in patients receiving repeated intravitreal injections for age-related macular degeneration
NCT02139033	N/A	42	Retaine™	Emulsion	Phase 4	DED

**Table 5:** Marketed ophthalmic emulsions approved in USA, Europe, and/or Canada.

Name of Product	Manufacturer	Type of Formulation	Active Pharmaceutical Ingredient	Therapeutic Use/Disease Target	Year of Approval	Reference
Restasis^®^	AbbVie Corporation	Nanoemulsion	Cyclosporine A	Dry eye disease	2002	[1]
Durezol^®^	Novartis Pharmaceuticals	Nanoemulsion	Difluprednate	Postoperative ocular inflammation	2008	[1]
Cationorm^®^	Novagali Pharma	Cationic NE	Mineral oils, glycerol, tyloxapol, Poloxamer 188, tris hydrochloride, tromethamine, cetalkonium chloride	Hydrating and lubricating emulsion	2019	[3]
Ikervis^®^	Santen Pharmaceutical	Cationic NE	Cyclosporin A	Keratitis	2015	[3]
Xelpros^®^	Sun Pharma	Microemulsion	Latanoprost	Glaucoma	2018	[1]
Verkazia^®^	Santen Pharmaceutical	Nanoemulsion	Cyclosporine	Vernal keratoconjunctivitis	2021	[1]

## Data Availability

No new data were created or analyzed in this study. Data sharing is not applicable to this article.

## Abbreviations

The following abbreviations are used in this manuscript:

%T	Percentage transmittance
AFM	Atomic force microscopy
AMD	Age-related macular degeneration
API	Active pharmaceutical ingredient
ARPE-19	Adult retinal pigment epithelial-19 cells
ATR-FTIR	Attenuated total reflectance–Fourier transform infrared spectroscopy
AUC	Area under the curve
BAB	Blood–aqueous barrier
BBD	Box–Behnken designs
BC	Bruch’s–choroid
BCOP	Bovine corneal opacity and permeability
BHT	Butylated hydroxytoluene
BRB	Blood–retinal barrier
BVB	Blood–vitreous barrier
CCD	Central composite design
CCK-8	Cell Counting Kit-8
CLSM	Confocal laser scanning microscopy
CMAs	Critical material attributes
C_max_	Peak plasma concentration
CPPs	Critical processing parameters
CQAs	Critical quality attributes
CTAB	Cetrimonium bromide
CTAB	Cetyl trimethyl ammonium bromide
DAPI	4′,6-diamidino-2-phenylindole
DED	Dry eye disease
DHA	Docosahexaenoic acid
DLS	Dynamic light scattering
DoE	Design of Experiment
DOPE	1,2- di-(9Z-octadecenoyl)-sn-glycero-3-phosphoethanolamine
DOTAP	1,2-dioleoyl-3-trimethylammonium propane
DR	Diabetic retinopathy
DSC	Differential scanning calorimetry
DSPE-PEG 2000	Distearoylphosphatylethanolamine-polyethyleneglycol 2000
EDX	Energy-dispersive X-ray spectroscopy
Er	Enhancement ratio
FTIR	Fourier transform infrared spectroscopy
GHS	Global Harmonized System
GRAS	Generally recognized as safe
HCE-2	Human corneal epithelial cells
HCECs	Human corneal epithelial cells
HCLEs	Human corneal limbal epithelial cells
HET-CAM	Hen’s egg test–chorioallantoic membrane
HLB	Hydrophilic–lipophilic balance
HPLC	High-performance liquid chromatography
HPMC	Hydroxypropyl methylcellulose
HRSEM	High-resolution scanning electron microscopy
HRTEM	High-resolution transmission electron microscopy
HUVECs	Human umbilical vein endothelial cells
ICE	Isolated chicken eye
ICH	International Conference on Harmonization
ICR	Institute of Cancer Research
IGF	Insulin-like growth factor 1
IL-6	Interleukin-6
iNOS	Inducible nitric oxide synthase
IOP	Intraocular pressure
IPM	Isopropyl myristate
J_max_	Maximum flux
J_ss_	Steady-state flux
k_el_	Elimination rate constant
kp	Permeability coefficient
LAL	Limulus amebocyte lysate
LC	Loading capacity
MC-MS	Liquid chromatography–mass spectrometry
MCTs	Medium-chain triglycerides
MEs	Microemulsions
MMP-14	Matrix metalloproteinase
MNV	Macular neovascularization
MRP	Multidrug resistance protein
MTT	3-(4,5-dimethylthiazol-2-yl)-2,5-diphenyltetrazolium bromide
nAMD	Neovascular age-related macular degeneration
NDDSs	Novel drug delivery systems
NEs	Nanoemulsions
NF-κB	Nuclear factor kappa B
NMR	Nuclear magnetic resonance
NPDR	Non-proliferative diabetic retinopathy
NZW	New Zealand white
OECD	Organization for Economic Cooperation and Development
P-gp	Permeability glycoprotein
PBS	Phosphate-buffered saline
PDI	Polydispersity index
PDR	Proliferative diabetic retinopathy
PDT	Photodynamic therapy
PEG	Polyethylene glycol
PRP	Panretinal photocoagulation
QbD	Quality by Design
RBCs	Red blood cells
RGCs	Retinal ganglion cells
RPE	Retinal pigment epithelium
RSM	Response surface methodology
SAED	Selected area electron diffraction
SEDDSs	Self-emulsifying drug delivery systems
SIRC	Statens Seruminstitut rabbit cornea
SMEDDSs	Self-microemulsifying drug delivery systems
S_mix_	Surfactant–cosurfactant mixture
SNEDDSs	Self-nanoemulsifying drug delivery systems
SPM	Scanning probe microscopy
STF	Simulated tear fluid
t_1/2_	Half-life
TEER	Transepithelial electrical resistance
TEM	Transmission electron microscopy
TGA	Thermo-gravimetric analysis
T_gel_	Gelation temperature
T_max_	Time to achieve maximum drug concentration
TNF-α	Tumor necrosis factor-α
TPGS	D-α-tocopherol polyethylene glycol succinate
VEGF	Vascular endothelial growth factor
VEGFR1	Vascular endothelial growth factor receptor 1
VEGFR2	Vascular endothelial growth factor receptor 2
Za_vg_	Globule size
ZP	Zeta potential
